# Supplementary material for: Novel 1,3,4-Oxadiazole Derivatives Containing a Cinnamic Acid Moiety as Potential Bactericide for Rice Bacterial Diseases
Source: Int J Mol Sci. 2019 Feb 26;20(5):1020. doi: 10.3390/ijms20051020 (PMC6429232; doi:10.3390/ijms20051020)
Supplement: Supplementary file 1 [file ijms-20-01020-s001.pdf]

# Supplementary

## 1. Synthesis of the target compound

### *1.1 Chemistry*

All reagents and solvents were analytical grade or chemically pure. Melting points (mp.) of the products were determined using an XT-4 binocular microscope (Beijing Tech. Instruments Co., Beijing, China) and are uncorrected. Nuclear magnetic resonance ($^1$H NMR and $^{13}$C NMR) spectra were recorded on a 400 MHz (Bruker biospin AG, Magnet system 400 / 54 ascend) or a 500 MHz (JEOL-ECX 500 NMR spectrometer Tokyo, Japan) in $CDCl_3$ or DMSO-$d_6$ using tetramethylsilane (TMS) as the internal standard. The following abbreviations were used to designate chemical shift multiplicities: s = singlet, d = doublet, t = triplet, q = quartet, m = multiplet. All first−order splitting patterns were assigned based on the appearance of the multiplet. Splitting patterns that could not be easily interpreted were designated as multiplet (m). HRMS were obtained on a Thermo Scientific Q Exactive (Thermo Scientific, Missouri, MO). Reaction progress was observed by thin-layer chromatography (TLC) analysis on silica gel GF254. Column chromatographic purification was carried out using silica gel (200–300 mesh). Optical densitometry was measured on a Model 680 microplate reader (BIO-RAD, Hercules, CA, USA). The physical characteristics, $^1$H NMR, $^{13}$C NMR, and HRMS data for all the target compounds **5a** – **5ae** are shown below.

### *1.2 General procedure for the synthesis of intermediates **2-3***

Intermediate **2** was prepared according to the known method. Intermediate **3** was prepared according to the following method. Carbon disulfide (0.75 mol) was slowly added dropwise to a three-necked flask containing KOH (0.6 mol) of ethanol (500 mL) at room temperature to form potassium ethylxanthate. Then, intermediate **2** (0.5 mol) was added, and the reaction was heated to 40–50 °C and heated to reflux. After the reaction was completed, the reaction mixture was spin-dried to remove the solvent ethanol to give a dark brown powdery solid in 85–90% yields.

### *1.3 General procedure for the synthesis of intermediates **4***

Intermediate **3** (1.0 mmol) and potassium carbonate (3.0 mmol) were added to a three-necked flask containing DMF (8 mL) and stirred at room temperature, and $R_2X$ (1.1 mmol) was slowly added dropwise. The mixture was raised to 70–80 °C and stirred continuously for 3–5 h. After the reaction was completed and cooled to 20–25 °C, the reaction solution was dropped into water, stirred and dispersed, and filtered to obtain a yellow solid powder Intermediates **4**.

### *1.4 General procedure for the title compounds **5a-5ae***

The intermediate **4** (1 mmol) dissolved in ethanol (10 mL), after which a solution of ammonium molybdate (0.2 mmol) in 30% hydrogen peroxide (20 mmol) was added and stirred for 5 hours (as monitored by TLC). The solvent was removed under vacuum, a saturated aqueous solution of sodium bicarbonate was added to adjust pH to 8–9, then the mixture was extracted with $CH_2Cl_2$, drying and evaporating the solvent under reduced pressure to obtain a yellow solid. Also, the crude product was recrystallized from ethanol to give the pure target compounds **5a-5ae** in 48.9–89.3% yields.

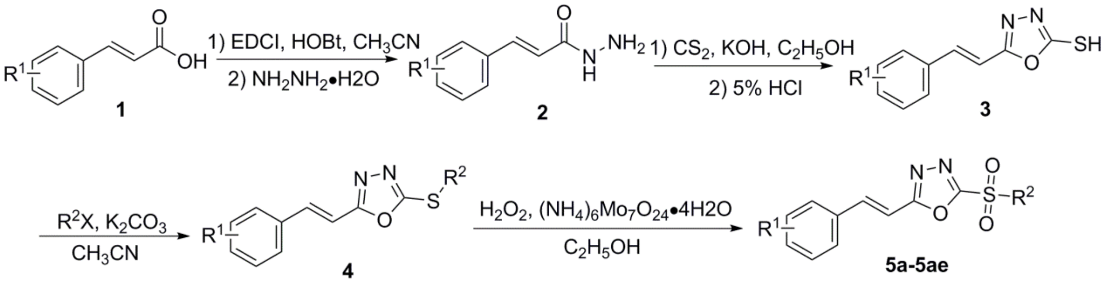

**5a**: $R^1$= H, $R^2$= $CH_3$
**5b**: $R^1$= H, $R^2$= $C_2H_5$
**5c**: $R^1$= H, $R^2$= $n$-$C_3H_7$
**5d**: $R^1$= H, $R^2$= $i$-$C_3H_7$
**5e**: $R^1$= H, $R^2$= $n$-$C_4H_9$
**5f**: $R^1$= H, $R^2$= $i$-$C_4H_9$
**5g**: $R^1$= H, $R^2$= $s$-$C_4H_9$
**5h**: $R^1$= H, $R^2$= $n$-$C_5H_{11}$
**5i**: $R^1$= 3-F, $R^2$= $CH_3$
**5j**: $R^1$= 3-F, $R^2$= $C_2H_5$
**5k**: $R^1$= 3-F, $R^2$= $i$-$C_3H_7$
**5l**: $R^1$= 3-F, $R^2$= $n$-$C_5H_{11}$
**5m**: $R^1$= 4-F, $R^2$= $CH_3$
**5n**: $R^1$= 4-F, $R^2$= $C_2H_5$
**5o**: $R^1$= 4-F, $R^2$= $i$-$C_3H_7$
**5p**: $R^1$= 4-F, $R^2$= $n$-$C_5H_{11}$
**5q**: $R^1$= 4-Cl, $R^2$= $CH_3$
**5r**: $R^1$= 4-Cl, $R^2$= $C_2H_5$
**5s** : $R^1$= 4-Br, $R^2$= $CH_3$
**5t**: $R^1$= 4-Br, $R^2$= $C_2H_5$
**5u**: $R^1$= 4-$OCH_3$, $R^2$= $CH_3$
**5v**: $R^1$= 4-$OCH_3$, $R^2$= $C_2H_5$
**5w**: $R^1$= H, $R^2$= benzyl
**5x**: $R^1$= 4-F, $R^2$= benzyl
**5y**: $R^1$= H, $R^2$=3-$CH_3$-benzyl
**5z**: $R^1$= H, $R^2$= 4-F-benzyl
**5aa**: $R^1$= 4-$OCH_3$, $R^2$= 4-F-benzyl
**5ab**: $R^1$= H, $R^2$= 4-Cl-benzyl
**5ac**: $R^1$= H, $R^2$= 2,4-di-Cl-benzyl
**5ad**: $R^1$= 4-F, $R^2$= 2,4-di-Cl-benzyl
**5ae**: $R^1$= H, $R^2$= 4-$NO_2$-benzyl

**Scheme 1.** Synthesis of the target compounds **5a-5ae**

## 2. The characterization data of the target compound

The characterization and physical and analytical data of compounds were shown as below.

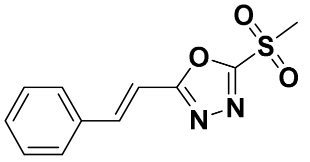

**5a**

Data for (*E*)-2-(methylsulfonyl)-5-styryl-1,3,4-oxadiazole (**5a**). White solid; yield 83.3%; m.p. 127–128°C; $^1$H NMR (400 MHz, $CDCl_3$, ppm) $\delta$ 7.74 (d, $J$ = 16.5 Hz, 1H, Ph-CH=C), 7.60–7.55 (m, 2H, Ph-2,6-H), 7.44 (dd, $J$ = 6.6, 3.5 Hz, 3H, Ph-3,4,5-H), 7.04 (d, $J$ = 16.5 Hz, 1H, Ph-C=CH), 3.51 (s, 3H, -S-$CH_3$). $^{13}$C NMR (100 MHz, $CDCl_3$, ppm) $\delta$ 166.22, 161.42, 143.12, 133.89, 131.02, 129.21, 128.03, 108.01, 42.98. HRMS (ESI) $[M+H]^+$ calcd for $C_{11}H_{10}N_2O_3S$: 251.0478, found: 251.0485.

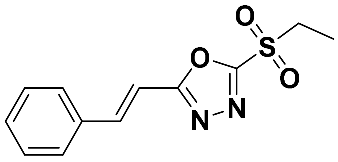

**5b**

Data for (*E*)-2-(ethylsulfonyl)-5-styryl-1,3,4-oxadiazole (**5b**). White solid; yield 64.6%; m.p. 124–125°C; $^1$H NMR (400 MHz, $CDCl_3$, ppm) $\delta$ 7.76 (d, $J$ = 16.5 Hz, 1H, Ph-CH=C), 7.60–7.55 (m, 2H, Ph-2,6-H), 7.44 (dd, $J$ = 6.6, 3.5 Hz, 3H, Ph-3,4,5-H), 7.06 (d, $J$ = 16.5 Hz, 1H, Ph-C=CH), 3.60 (q, $J$ = 7.4 Hz, 2H, -S-$CH_2$-), 1.54 (t, $J$ = 7.4 Hz, 3H, -$CH_3$). $^{13}$C NMR (100 MHz, $CDCl_3$, ppm) $\delta$ 166.26, 160.59, 143.10, 133.92, 131.01, 129.22, 128.01, 108.07, 50.05, 6.87. HRMS (ESI) $[M+H]^+$ calcd for $C_{12}H_{12}N_2O_3S$: 265.0637,

found: 265.0641.

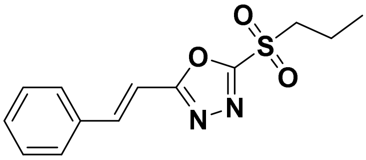

**5c**

Data for (*E*)-2-(propylsulfonyl)-5-styryl-1,3,4-oxadiazole (**5c**). White solid; yield, 49.1%; m.p. 97.8–98.5 °C; $^1$H NMR (400 MHz, $CDCl_3$, ppm) *δ* 7.76 (d, *J* = 16.5 Hz, 1H, Ph-CH=C), 7.61–7.56 (m, 2H, Ph-2,6-H), 7.47–7.41 (m, 3H, Ph-3,4,5-H), 7.06 (d, *J* = 16.5 Hz, 1H, Ph-C=CH), 3.57–3.51 (m, 2H, $-S-CH_2-$), 2.01 (m, *J* = 15.1, 7.5 Hz, 2H, $-S-CH_2\underline{CH_2}CH_3$), 1.14 (t, *J* = 7.5 Hz, 3H, $-CH_3$). $^{13}$C NMR (100 MHz, $CDCl_3$, ppm) *δ* 166.20, 161.03, 143.04, 133.96, 130.97, 129.20, 127.98, 108.12, 56.97, 15.97, 12.84. HRMS (ESI) $[M+H]^+$ calcd for $C_{13}H_{14}N_2O_3S$: 279.0794, found: 279.0798.

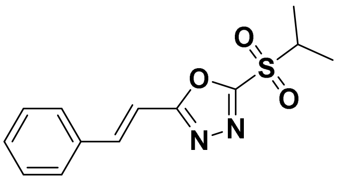

**5d**

Data for (*E*)-2-(isopropylsulfonyl)-5-styryl-1,3,4-oxadiazole (**5d**). White solid; yield, 45.2%; m.p. 99.2–100.9 °C; $^1$H NMR (400 MHz, $CDCl_3$, ppm) *δ* 7.76 (d, *J* = 16.5 Hz, 1H, Ph-CH=C), 7.61–7.56 (m, 2H, Ph-2,6-H), 7.47–7.42 (m, 3H, Ph-3,4,5-H), 7.07 (d, *J* = 16.5 Hz, 1H, Ph-C=CH), 3.75–3.64 (m, 1H, -S-CH-), 1.53 (d, *J* = 6.9 Hz, 6H,$\underline{CH_3}CH\underline{CH_3}$). $^{13}$C NMR (100 MHz, $CDCl_3$, ppm) *δ* 166.35, 160.10, 143.02, 133.94, 130.98, 129.21, 127.99, 108.15, 56.37, 15.03. HRMS (ESI) $[M+H]^+$ calcd for $C_{13}H_{14}N_2O_3S$: 279.0794, found: 279.0798.

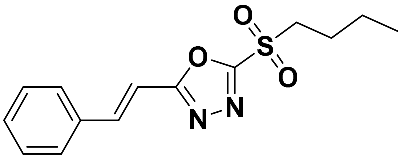

**5e**

Data for (*E*)-2-(butylsulfonyl)-5-styryl-1,3,4-oxadiazole (**5e**). White solid; yield 89.3%; m.p. 73.0–75.0 °C; $^1$H NMR (500 MHz, $CDCl_3$, ppm) *δ* 7.76 (d, *J* = 16.5 Hz, 1H, Ph-CH=C), 7.61–7.55 (m, 2H, Ph-2,6-H), 7.46–7.41 (m, 3H, Ph-3,4,5-H), 7.06 (d, *J* = 16.4 Hz, 1H, Ph-C=CH), 3.58–3.52 (m, 2H, $-S\underline{CH_2}CH_2CH_2CH_3$), 1.94 (dt, *J* = 15.2, 7.7 Hz, 2H, $-SCH_2\underline{CH_2}CH_2CH_3$), 1.52 (dd, *J* = 14.8, 7.4 Hz, 2H, $-SCH_2CH_2\underline{CH_2}CH_3$), 0.99 (t, *J* = 8.1 Hz, 3H, $-SCH_2CH_2CH_2\underline{CH_3}$). $^{13}$C NMR (125 MHz, $CDCl_3$, ppm) *δ* 166.26, 161.10, 143.12, 134.03, 131.05, 129.27, 128.06, 108.19, 55.20, 23.94, 21.54, 13.49. HRMS (ESI) $[M+H]^+$ calcd for $C_{14}H_{16}N_2O_3S$: 293.0949, found: 293.0954.

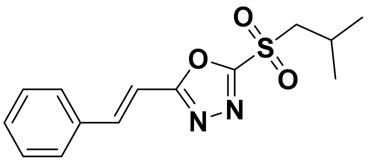

**5f**

Data for (*E*)-2-(isobutylsulfonyl)-5-styryl-1,3,4-oxadiazole (**5f**). White solid; yield 88.3%; m.p. 101.6–102.3°C; $^{1}$H NMR (400 MHz, $CDCl_3$, ppm) $\delta$ 7.76 (d, *J* = 16.5 Hz, 1H, Ph-CH=C), 7.50–7.40 (m, 2H, Ph-2,6-H), 7.48–7.42 (m, 3H, Ph-3,4,5-H), 7.06 (d, *J* = 16.4 Hz, 1H, Ph-C=CH), 3.49 (d, *J* = 6.6 Hz, 2H, -S$CH_2$CH$(CH_3)_2$), 2.57–2.43 (m, 1H, -S$CH_2$CH$(CH_3)_2$), 1.19 (d, *J* = 6.7 Hz, 6H, -S$CH_2$CH$(CH_3)_2$). $^{13}$C NMR (100 MHz, $CDCl_3$, ppm) $\delta$ 166.16, 161.47, 143.00, 133.92, 131.00, 129.22, 128.00, 108.10, 62.68, 23.98, 22.57. HRMS (ESI) $[M+H]^+$ calcd for $C_{14}H_{16}N_2O_3S$: 293.0951, found: 293.0954.

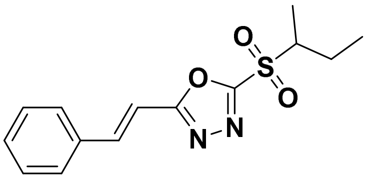

**5g**

Data for(*E*)-2-(*sec*-butylsulfonyl)-5-styryl-1,3,4-oxadiazole (**5g**). White solid; yield 48.9%; m.p. 110–111 °C; $^{1}$H NMR (400 MHz, $CDCl_3$, ppm) $\delta$ 7.76 (d, *J* = 16.5 Hz, 1H, Ph-CH=C), 7.62–7.56 (m, 2H, Ph-2,6-H), 7.49–7.40 (m, 3H, Ph-3,4,5-H), 7.07 (d, *J* = 16.5 Hz, 1H, Ph-C=CH), 3.48 (dqd, *J* = 13.8, 6.9, 3.9 Hz, 1H, -SCH$(CH_3)CH_2CH_3$), 2.18 (dqd, *J* = 15.1, 7.6, 3.9 Hz, 1H, -SCH$(CH_3)CH_2CH_3$), 1.70 (ddq, *J* = 14.7, 9.6 ,7.4 Hz, 1H, -SCH$(CH_3)CH_2CH_3$), 1.51 (d, *J* = 6.9 Hz, 3H, -SCH$(CH_3)CH_2CH_3$), 1.11 (t, *J* = 7.5 Hz, 3H, -SCH$(CH_3)CH_2CH_3$). $^{13}$C NMR (100 MHz, $CDCl_3$, ppm) $\delta$ 166.35, 160.31, 142.98, 133.92, 130.99, 129.21, 128.00, 108.15, 62.06, 22.03, 12.15, 10.95. HRMS (ESI) $[M+H]^+$ calcd for $C_{14}H_{16}N_2O_3S$: 293.0951, found: 293.0954.

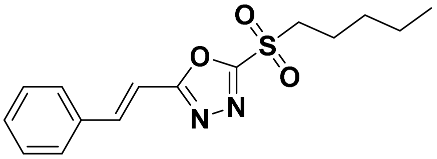

**5h**

Data for (*E*)-2-(pentylsulfonyl)-5-styryl-1,3,4-oxadiazole (**5h**). White solid; yield 89.2%; m.p. 86.5–87.2 °C; $^{1}$H NMR (400 MHz, $CDCl_3$, ppm) $\delta$ 7.76 (d, *J* = 16.5 Hz, 1H, Ph-CH=C), 7.62–7.56 (m, 2H, Ph-2,6-H), 7.48–7.40 (m, 3H, Ph-3,4,5-H), 7.06 (d, *J* = 16.5 Hz, 1H, Ph-C=CH), 3.59–3.52 (m, 2H, -S$CH_2CH_2CH_2CH_2CH_3$), 1.96 (dt, *J* = 15.7, 7.7 Hz, 2H, -S$CH_2CH_2CH_2CH_2CH_3$), 1.53–1.44 (m, 2H, -S$CH_2CH_2CH_2CH_2CH_3$), 1.38 (dq, *J* = 14.3, 7.0 Hz, 2H, -S$CH_2CH_2CH_2CH_2CH_3$), 0.93 (t, *J* = 7.2 Hz, 3H, -S$CH_2CH_2CH_2CH_2CH_3$). $^{13}$C NMR (100 MHz, $CDCl_3$, ppm) $\delta$ 166.20, 161.06, 143.06, 133.98, 130.98, 129.22, 128.00, 108.13, 55.36, 30.21, 22.03, 21.64, 13.64. HRMS (ESI) $[M+H]^+$ calcd for $C_{15}H_{18}N_2O_3S$: 307.1105, found: 307.1110.

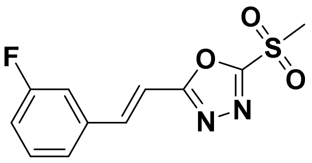


**5i**

Data for (*E*)-2-(3-fluorostyryl)-5-(methylsulfonyl)-1,3,4-oxadiazole (**5i**). White solid; yield, 63.5%; m.p. 129-131 °C; $^1$H NMR (400 MHz, $CDCl_3$, ppm) *δ* 7.77 (d, *J* = 16.5 Hz, 1H, Ph-CH=C), 7.51–7.48 (m, *J* = 7.9, 5.7 Hz, 1H, Ar-H), 7.36 (d, *J* = 7.8 Hz, 1H, Ar-H), 7.31–7.27 (m, 1H, Ar-H), 7.22–7.18 (m, *J* = 8.3, 2.5, 1.0 Hz, 1H, Ar-H), 7.06 (d, *J* = 16.4 Hz, 1H, Ph-C=CH), 3.52 (s, 3H, S-$CH_3$). $^{13}$C NMR (101 MHz, $CDCl_3$, ppm) *δ* 165.78, 163.11 (d, *J* = 247.7 Hz), 161.59, 141.71 (d, *J* = 2.8 Hz), 136.04 (d, *J* = 7.7 Hz), 130.84 (d, *J* = 8.2 Hz), 124.05 (d, *J* = 2.9 Hz), 117.94 (d, *J* = 21.4 Hz), 114.25 (d, *J* = 22.2 Hz), 109.41, 42.95. HRMS (ESI) $[M+H]^+$ calcd for $C_{11}H_9FN_2O_3S$: 269.0388, found: 269.0391.

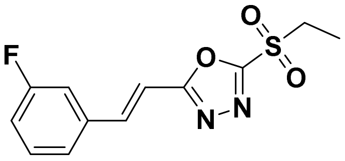


**5j**

Data for (*E*)-2-(ethylsulfonyl)-5-(3-fluorostyryl)-1,3,4-oxadiazole (**5j**). White solid; yield 60.0%; m.p. 74–75 °C; $^1$H NMR (400 MHz, $CDCl_3$, ppm) *δ* 7.72 (d, *J* = 16.5 Hz, 1H, Ph-CH=C), 7.45–7.40(m, 1H, Ar-H), 7.36 (d, *J* = 7.8 Hz, 1H, Ar-H), 7.29 (dd, *J* = 10.1, 2.5 Hz, 1H, Ar-H), 7.15 (dt, *J* = 8.3, 2.5 Hz, 1H, Ar-H), 7.07 (d, *J* = 16.4 Hz, 1H, Ph-C=CH), 3.61 (q, *J* = 7.4 Hz, 2H, S-$\underline{CH_2}$), 1.55 (t, *J* = 7.4 Hz, 3H, -$CH_2\underline{CH_3}$). $^{13}$C NMR (100 MHz, $CDCl_3$, ppm) *δ* 165.82, 163.10 (d, *J* = 247.6 Hz), 160.77, 141.63 (d, *J* = 2.8 Hz), 136.08 (d, *J* = 7.7 Hz), 130.83 (d, *J* = 8.3 Hz), 124.04 (d, *J* = 2.9 Hz), 117.89 (d, *J* = 21.4 Hz), 114.24 (d, *J* = 22.2 Hz), 109.50, 50.05, 6.86. HRMS (ESI) $[M+H]^+$ calcd for $C_{12}H_{11}FN_2O_3S$: 283.0542, found: 283.0547.

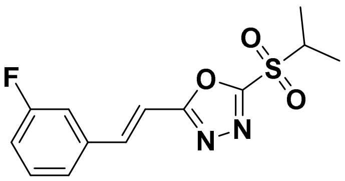


**5k**

Data for (*E*)-2-(3-fluorostyryl)-5-(isopropylsulfonyl)-1,3,4-oxadiazole (**5k**). White solid; yield 86.5%; m.p. 100.8–102.3 °C; $^1$H NMR (400 MHz, $CDCl_3$, ppm) *δ* 7.72 (d, *J* = 16.5 Hz, 1H, Ph-CH=C), 7.43 (td, *J* = 7.9, 5.7Hz, 1H, Ar-H), 7.36 (d, *J* = 7.8 Hz, 1H, Ar-H), 7.31–7.26 (m, 1H, Ar-H), 7.15 (td, *J* = 8.2Hz, 1H, Ar-H), 7.07 (d, *J* = 16.4 Hz, 1H, Ph-C=CH), 3.77–3.65 (m, 1H, S-CH-), 1.53 (d, *J* = 6.9 Hz, 6H, $\underline{CH_3}CH\underline{CH_3}$). $^{13}$C NMR (100 MHz, $CDCl_3$, ppm) *δ* 165.90, 163.11 (d, *J* = 247.7 Hz), 160.29, 141.54, 136.10 (d, *J* = 7.7 Hz), 130.83 (d, *J* = 8.3 Hz), 123.99, 117.87 (d, *J* = 21.5 Hz), 114.23 (d, *J* = 22.2 Hz), 109.57, 56.40, 15.02. HRMS (ESI) $[M+H]^+$ calcd for $C_{13}H_{13}FN_2O_3S$: 297.0700, found: 297.0704.

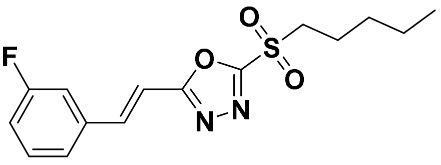

**5l**

Data for (*E*)-2-(3-fluorostyryl)-5-(pentylsulfonyl)-1,3,4-oxadiazole (**5l**). White solid; yield 74.0%; m.p. 97.1-98.3 °C; $^1$H NMR (400 MHz, $CDCl_3$, ppm) *δ* 7.72 (d, *J* = 16.5 Hz, 1H, Ph-CH=C), 7.46–7.39 (m, 1H, Ar-H), 7.36 (d, *J* = 7.8 Hz, 1H, Ar-H), 7.28 (d, *J* = 8.6 Hz, 1H, Ar-H), 7.14 (td, *J* = 8.7, 2.8 Hz, 1H, Ar-H), 7.06 (d, *J* = 16.4 Hz, 1H, Ph-C=CH), 3.63–3.51 (m, 2H, -S$\underline{CH_2}$$CH_2CH_2CH_2CH_3$), 1.96 (m, *J* = 15.5 Hz, 2H, -$SCH_2$$\underline{CH_2}$$CH_2CH_2CH_3$), 1.52–1.44 (m, 2H, -$SCH_2CH_2$$\underline{CH_2}$$CH_2CH_3$), 1.38 (m, *J* = 14.1 Hz, 2H, -$SCH_2CH_2CH_2$$\underline{CH_2}$$CH_3$), 0.93 (t, *J* = 7.2 Hz, 3H, -$SCH_2CH_2CH_2CH_2$$\underline{CH_3}$). $^{13}$C NMR (100 MHz, $CDCl_3$, ppm) *δ* 165.75, 163.10 (d, *J* = 247.6 Hz), 161.18, 141.54 (d, *J* = 2.8 Hz), 136.11 (d, *J* = 7.7 Hz), 130.82 (d, *J* = 8.3 Hz), 124.02 (d, *J* = 2.9 Hz), 117.85 (d, *J* = 21.4 Hz), 114.22 (d, *J* = 22.2 Hz), 109.54, 55.32, 30.17, 22.02, 21.61, 13.65. HRMS (ESI) $[M+H]^+$ calcd for $C_{15}H_{17}FN_2O_3S$: 325.1011, found: 325.1017.

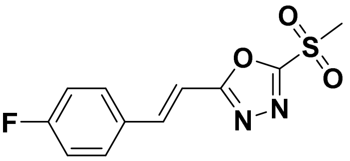

**5m**

Data for (*E*)-2-(4-fluorostyryl)-5-(methylsulfonyl)-1,3,4-oxadiazole (**5m**). White solid; yield 55.8%; m.p. 146–147 °C; $^1$H NMR (400 MHz, DMSO-$d_6$, ppm) *δ* 7.72 (d, *J* = 16.5 Hz, 1H, Ph-CH=C), 7.61–7.56 (m, 2H, Ph-2,6-H), 7.18–7.11 (m, 2H, Ph-3,5-H), 6.98 (d, *J* = 16.4 Hz, 1H, Ph-C=CH ), 3.52 (s, 3H, S-$CH_3$). $^{13}$C NMR (100 MHz, $CDCl_3$, ppm) *δ* 166.08, 164.28 (d, *J* = 253.0 Hz), 161.42, 141.80, 130.18 (d, *J* = 3.4 Hz), 129.98 (d, *J* = 8.6 Hz), 116.47 (d, *J* = 22.1 Hz), 107.74 (d, *J* = 2.5 Hz), 42.94. HRMS (ESI) $[M+H]^+$ calcd for $C_{11}H_9FN_2O_3S$: 269.0389, found: 269.0391.

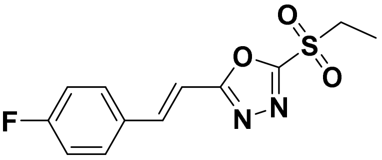

**5n**

Data for (*E*)-2-(ethylsulfonyl)-5-(4-fluorostyryl)-1,3,4-oxadiazole (**5n**). White solid; yield 62.37%; m.p. 179–180 °C; $^1$H NMR (500 MHz, DMSO-$d_6$, ppm) δ 7.93–7.87 (m, 2H, Ph-2,6-H), 7.78 (d, *J* = 16.5 Hz, 1H, Ph-CH=C), 7.42 (d, *J* = 16.5 Hz, 1H, Ph-C=CH), 7.31–7.25 (m, 2H, Ph-3,5-H), 3.75 (q, *J* = 7.3 Hz, 2H, S-$CH_2$-), 1.30 (t, *J* = 7.4 Hz, 3H, -$CH_3$). $^{13}$C NMR (125 MHz, DMSO-$d_6$, ppm) *δ* 166.60, 163.93 (d, *J* = 249.2 Hz), 160.85, 141.41, 131.45 (d, *J* = 2.4 Hz), 131.25 (d, *J* = 8.6 Hz), 116.61 (d, *J* = 21.9 Hz), 109.39, 49.98, 7.15. HRMS (ESI) $[M+H]^+$ calcd for $C_{12}H_{11}FN_2O_3S$: 283.0543, found: 283.0547.

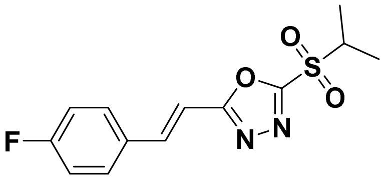

**5o**

Data for (*E*)-2-(4-fluorostyryl)-5-(isopropylsulfonyl)-1,3,4-oxadiazole (**5o**). White solid; yield 86.7%; m.p. 103.9–104.9 °C; $^1$H NMR (400 MHz, $CDCl_3$, ppm) *δ* 7.73 (d, *J* = 16.5 Hz, 1H, Ph-CH=C), 7.62–7.55 (m, 2H, Ph-2,6-H), 7.14 (t, *J* = 8.6 Hz, 2H, Ph-3,5-H), 6.99 (d, *J* = 16.4 Hz, 1H, Ph-C=CH), 3.75–3.65 (m, 1H, S-CH-), 1.53 (d, *J* = 6.9 Hz, 6H, $\underline{CH_3}CH\underline{CH_3}$). $^{13}$C NMR (100 MHz, $CDCl_3$, ppm) *δ* 166.19, 164.26 (d, *J* = 252.9 Hz), 160.11, 141.65, 130.24 (d, *J* = 3.4 Hz), 129.94 (d, *J* = 8.6 Hz), 116.46 (d, *J* = 22.1 Hz), 107.91 (d, *J* = 2.5 Hz), 56.38, 15.02. HRMS (ESI) $[M+H]^+$ calcd for $C_{13}H_{13}FN_2O_3S$: 297.0699, found: 297.0704.

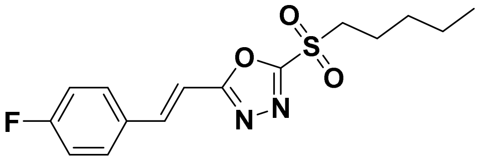

**5p**

Data for (*E*)-2-(4-fluorostyryl)-5-(pentylsulfonyl)-1,3,4-oxadiazole (**5p**). White solid; yield 63.2%; m.p. 83–84 °C; $^1$H NMR (400 MHz, $CDCl_3$, ppm) *δ* 7.72 (d, *J* = 16.5 Hz, 1H, Ph-CH=C), 7.59 (d, *J* = 8.2 Hz, 2H, Ph-2,6-H), 7.14 (t, *J* = 8.5 Hz, 2H, Ph-3,5-H), 6.98 (d, *J* = 16.4 Hz, 1H, Ph-C=CH), 3.59–3.52 (m, 2H, $-S\underline{CH_2}CH_2CH_2CH_2CH_3$), 2.00–1.91 (m, 2H, $-SCH_2\underline{CH_2}CH_2CH_2CH_3$), 1.47 (dd, *J* = 15.2, 7.6 Hz, 2H, $-SCH_2CH_2\underline{CH_2}CH_2CH_3$), 1.38 (dq, *J* = 14.3, 7.2 Hz, 2H, $-SCH_2CH_2CH_2\underline{CH_2}CH_3$), 0.93 (t, *J* = 7.2 Hz, 3H, $-SCH_2CH_2CH_2CH_2\underline{CH_3}$). $^{13}$C NMR (100 MHz, $CDCl_3$, ppm) *δ* 166.05, 164.24 (d, *J* = 252.8 Hz), 161.01, 141.64, 130.24 (d, *J* = 3.4 Hz), 129.95 (d, *J* = 8.7 Hz), 116.44 (d, *J* = 22.1 Hz), 107.88 (d, *J* = 2.5 Hz), 55.31, 30.17, 22.02, 21.61, 13.64. HRMS (ESI) $[M+H]^+$ calcd for $C_{15}H_{17}FN_2O_3S$: 325.1012, found: 325.1017.

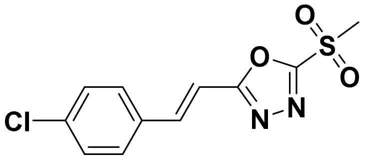

**5q**

Data for (*E*)-2-(4-chlorostyryl)-5-(methylsulfonyl)-1,3,4-oxadiazole (**5q**). White solid; yield 73.5%; m.p. 157–158 °C; $^1$H NMR (500 MHz, DMSO-$d_6$, ppm) *δ* 7.85 (dt, *J* = 8.2, 4.1 Hz, 2H, Ph-2,6-H), 7.78 (d, *J* = 15.4 Hz, 1H, Ph-CH=C), 7.51 (dd, *J* = 6.7, 1.7 Hz, 2H, Ph-3,5-H), 7.47 (d, 1H, Ph-C=CH), 3.66 (s, 3H, $S-CH_3$). $^{13}$C NMR (125 MHz, DMSO-$d_6$, ppm) *δ* 166.17, 161.99, 141.21, 135.72, 133.71, 130.55, 129.62, 110.28, 43.44. HRMS (ESI) $[M+H]^+$ calcd for $C_{11}H_9ClN_2O_3S$: 285.0091, found: 285.0095.

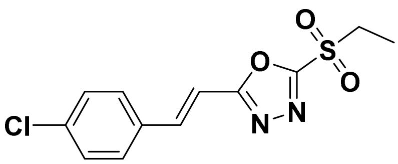

**5r**

Data for (*E*)-2-(4-chlorostyryl)-5-(ethylsulfonyl)-1,3,4-oxadiazole (**5r**). White solid; yield 73.1%; m.p.124–125 °C; $^1$H NMR (500 MHz, DMSO-$d_6$, ppm) $\delta$ 7.88–7.84 (m, 2H, Ph-2,6-H), 7.77 (d, $J$ = 16.5 Hz, 1H, Ph-CH=C), 7.53–7.48 (m, 3H, Ph-C=CH, Ph-3,5-H ), 3.75 (q, $J$ = 7.3 Hz, 2H, S-CH$_2$-), 1.29 (t, $J$ = 7.3 Hz, 3H, -CH$_3$). $^{13}$C NMR (125 MHz, DMSO-$d_6$, ppm) $\delta$ 166.49, 160.92, 141.20, 135.72, 133.71, 130.56, 129.60 110.34, 49.99 , 7.16 . HRMS (ESI) [M+H]$^+$ calcd for $C_{12}H_{11}ClN_2O_3S$: 299.0248, found: 299.0252.

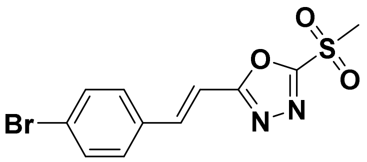

**5s**

Data for (*E*)-2-(4-bromostyryl)-5-(methylsulfonyl)-1,3,4-oxadiazole (**5s**). White solid; yield 85.2%; m.p. 158–159 °C; $^1$H NMR (500 MHz, DMSO-$d_6$, ppm) $\delta$ 7.79-7.75 (m, 3H, Ph-CH=C, Ph-2,6-H), 7.64 (d, $J$ = 8.3 Hz, 2H, Ph-3,5-H), 7.51 (d, $J$ = 16.5 Hz, 1H, Ph-C=CH), 3.66 (s, 3H, S-CH$_3$). $^{13}$C NMR (125 MHz, DMSO-$d_6$, ppm) $\delta$ 166.18, 161.96, 141.30, 134.03, 132.55, 130.77, 124.61, 110.34, 43.41. HRMS (ESI) [M+H]$^+$ calcd for $C_{11}H_9BrN_2O_3S$: 328.9581, found: 328.9590.

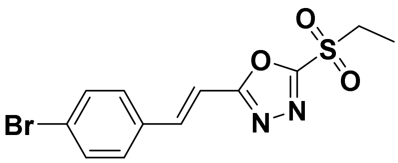

**5t**

Data for (*E*)-2-(4-bromostyryl)-5-(ethylsulfonyl)-1,3,4-oxadiazole (**5t**). White solid; yield 83.9%; m.p. 127–128 °C; $^1$H NMR (400 MHz, DMSO-$d_6$, ppm) $\delta$ 7.84–7.76 (m, 3H, Ph-CH=C, Ph-2,6-H ), 7.67 (dd, $J$ = 6.8, 1.4 Hz, 2H, Ph-3,5-H), 7.53 (d, 1H, $J$ = 16.5 Hz, Ph-C=CH), 3.78 (q, $J$ = 7.3 Hz, 2H, S-CH$_2$-), 1.34 (t, $J$ = 7.3 Hz, 3H, -CH$_3$). $^{13}$C NMR (100 MHz, DMSO-$d_6$, ppm) $\delta$ 166.43, 160.85, 141.25, 133.97, 132.47, 130.70, 124.54, 110.34, 49.91, 7.09. HRMS (ESI) [M+H]$^+$ calcd for $C_{12}H_{11}BrN_2O_3S$: 342.9740, found: 342.9747.

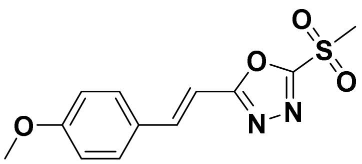

**5u**

Data for (*E*)-2-(4-methoxystyryl)-5-(methylsulfonyl)-1,3,4-oxadiazole (**5u**). White solid; yield 70.8%; m.p. 103–104 °C; $^1$H NMR (500 MHz, CDCl$_3$, ppm) $\delta$ 7.69 (d, $J$ = 16.4 Hz, 1H, Ph-CH=C), 7.53 (d, $J$ = 8.7 Hz, 2H, Ph-2,6-H), 6.95 (d, $J$ = 8.7 Hz, 2H, Ph-3,5-H), 6.89 (d, $J$ = 16.4 Hz, 1H, Ph-C=CH), 3.86 (s, 3H,-OCH$_3$), 3.49 (s, 3H, S-CH$_3$). $^{13}$C NMR (125 MHz, CDCl$_3$, ppm) $\delta$ 166.71, 161.85, 161.63, 142.41, 130.71, 127.39, 115.05, 106.63, 55.94, 43.38. HRMS (ESI) [M+H]$^+$ calcd for $C_{12}H_{12}N_2O_4S$: 281.0584, found: 281.0591.

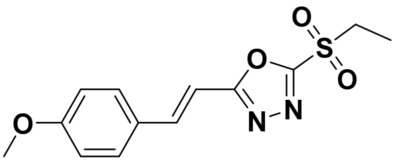

**5v**

Data for (*E*)-2-(ethylsulfonyl)-5-(4-methoxystyryl)-1,3,4-oxadiazole (**5v**). White solid; yield 74.7%; m.p. 109–110 °C; $^{1}$H NMR (500 MHz, $CDCl_3$, ppm) $\delta$ 7.70 (d, $J$ = 16.4 Hz, 1H, Ph-CH=C), 7.53 (d, $J$ = 8.7 Hz, 2H, Ph-2,6-H), 6.95 (dd, $J$ = 7.0, 4.4 Hz, 2H, Ph-3,5-H), 6.90 (d, $J$ = 16.4 Hz, 1H, Ph-C=CH), 3.86 (s, 3H, $-OCH_3$), 3.58 (q, $J$ = 7.4 Hz, 2H, $S-CH_2-$), 1.53 (t, $J$ = 7.4 Hz, 3H, $-CH_3$). $^{13}$C NMR (125 MHz, $CDCl_3$, ppm) $\delta$ 167.03, 161.85, 160.55, 142.43, 130.73, 127.40, 115.05, 106.71, 55.94, 49.91, 7.18. HRMS (ESI) $[M+H]^+$ calcd for $C_{13}H_{14}N_2O_4S$: 295.0743, found: 295.0747.

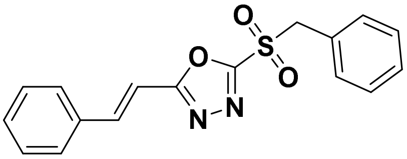

**5w**

Data for (*E*)-2-(benzylsulfonyl)-5-styryl-1,3,4-oxadiazole (**5w**). White solid; yield 76.6%; m.p. 164–165 °C; $^{1}$H NMR (400 MHz, DMSO-$d_6$, ppm) $\delta$ 7.83 (dd, $J$ = 6.5, 3.1 Hz, 2H, Ar-H), 7.69 (d, $J$ = 16.5 Hz, 1H, Ph-CH=C), 7.50–7.36 (m, 9H, Ar-H, Ph-C=CH), 5.22 (s, 2H, $-SCH_2$). $^{13}$C NMR (100 MHz, DMSO-$d_6$, ppm) $\delta$ 166.61, 160.88, 142.69, 134.56, 131.88, 131.21, 129.73, 129.63, 129.25.128.80, 126.47, 109.26, 61.11. HRMS (ESI) $[M+H]^+$ calcd for $C_{17}H_{14}N_2O_3S$: 327.0794, found:327.0798.

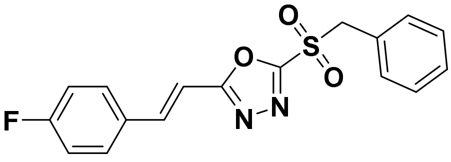

**5x**

Data for (*E*)-2-(benzylsulfonyl)-5-(4-fluorostyryl)-1,3,4-oxadiazole (**5x**). White solid; yield 57.3%; m.p. 150–151 °C; $^{1}$H NMR (500 MHz, DMSO-$d_6$, ppm) $\delta$ 7.88 (dd, $J$ = 8.4, 5.7 Hz, 2H, Ar-H), 7.66 (d, 1H, Ph-CH=C), 7.46 (d, $J$ = 8.3 Hz, 2H, Ar-H), 7.42–7.31 (m, 4H, Ar-H, Ph-C=CH), 7.28 (t, $J$ = 8.6 Hz, 2H, Ar-H), 5.22 (s, 2H, $-SCH_2$). $^{13}$C NMR (100 MHz, DMSO-$d_6$, ppm) $\delta$ 166.68, 163.97 (d, $J$ = 249.4 Hz), 160.75, 141.57, 134.85, 133.73, 131.31, 131.24, 129.38, 125.74, 116.64 (d, $J$ = 21.9 Hz), 109.19, 60.30. HRMS (ESI) $[M+H]^+$ calcd for $C_{17}H_{13}FN_2O_3S$: 345.0698, found: 345.0704.

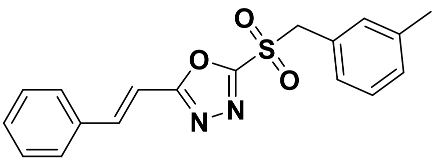

**5y**

Data for (*E*)-2-((3-methylbenzyl)sulfonyl)-5-styryl-1,3,4-oxadiazole (**5y**). White solid; yield 61.6%; m.p. 117–118 °C; $^{1}$H NMR (400 MHz, DMSO-$d_6$, ppm) $\delta$ 7.83 (dd, $J$ = 6.3, 2.8 Hz, 2H, Ar-H), 7.69 (d, $J$ =

16.5 Hz, 1H, Ph-CH=C), 7.51–7.43 (m, 4H, Ar-H), 7.29 (t, *J* = 7.7 Hz, 1H, Ar-H), 7.23 (d, *J* = 7.6 Hz, 1H, Ph-C=CH), 7.15 (d, *J* = 7.4 Hz, 2H, Ar-H), 5.16 (s, 2H, $-SCH_2-$), 2.28 (s, 3H, $Ar-CH_3$). $^{13}C$ NMR (100 MHz, DMSO-$d_6$, ppm) $\delta$ 166.58, 160.92, 142.67, 138.47, 134.57, 132.33, 131.22, 130.36, 129.55, 129.16, 128.95, 128.79,126.30, 109.26, 61.18, 21.30. HRMS (ESI) $[M+H]^+$ calcd for $C_{18}H_{16}N_2O_3S$: 341.0948, found: 341.0954.

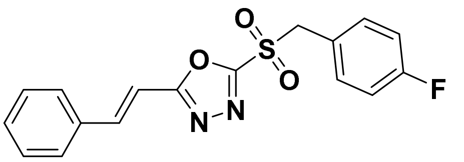

**5z**

Data for (*E*)-2-((4-fluorobenzyl)sulfonyl)-5-styryl-1,3,4-oxadiazole (**5z**). White solid; yield, 70.5%; m.p. 137–138 °C; $^1H$ NMR (400 MHz, DMSO-$d_6$, ppm) $\delta$ 7.83 (m, *J* = 6.5, 3.1 Hz, 2H, Ar-H), 7.70 (d, *J* = 16.5 Hz, 1H, Ph-CH=C), 7.52–7.41 (m, 6H, Ar-H), 7.32–7.24 (m, 2H, Ar-H, Ph-C=CH), 5.24 (s, 2H, $-SCH_2-$).$^{13}C$ NMR (100 MHz, DMSO-$d_6$, ppm) $\delta$ 166.65, 163.18 (d, *J* = 246.4 Hz), 160.76, 142.71, 134.56, 134.10 (d, *J* = 8.7 Hz), 131.23, 129.53, 128.81, 122.91, 116.26 (d, *J* = 21.8 Hz), 109.29 , 60.15. HRMS (ESI) $[M+H]^+$ calcd for $C_{17}H_{13}FN_2O_3S$: 345.0697, found: 345.0704.

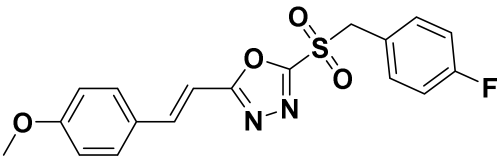

**5aa**

Data for (*E*)-2-((4-fluorobenzyl)sulfonyl)-5-(4-methoxystyryl)-1,3,4-oxadiazole (**5aa**). White solid; yield 77.4%; m.p. 169–170 °C; $^1H$ NMR (400 MHz, DMSO-$d_6$, ppm) $\delta$ 7.79 (d, *J* = 8.8 Hz, 2H, Ar-H), 7.64 (d, *J* = 16.4 Hz, 1H, Ph-CH=C), 7.48–7.40 (m, 2H, Ar-H), 7.31–7.23 (m, 3H, Ar-H), 7.04 (d, *J* = 8.8 Hz, 2H, Ar-H, Ph-C=CH), 5.23 (s, 2H, $-SCH_2-$), 3.83 (s, 3H,$-OCH_3$). $^{13}C$ NMR (100 MHz, DMSO-$d_6$, ppm) $\delta$ 167.03, 163.17 (d, *J* = 246.4 Hz), 161.87, 160.48, 142.51, 134.07 (d, *J* = 8.7 Hz), 130.67, 127.24, 122.93 (d, *J* = 3.1 Hz), 116.25 (d, *J* = 21.8 Hz), 115.01, 106.43, 60.14, 55.87. HRMS (ESI) $[M+H]^+$ calcd for $C_{18}H_{15}FN_2O_4S$: 375.0804, found: 375.0809.

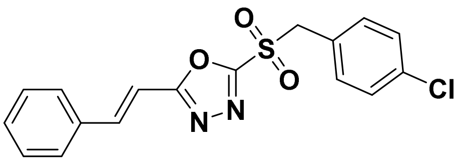

**5ab**

Data for (*E*)-2-((4-chlorobenzyl)sulfonyl)-5-styryl-1,3,4-oxadiazole (**5ab**). White solid; yield, 63.8%; m.p. 141–142 °C; $^1H$ NMR (400 MHz, DMSO-$d_6$, ppm) $\delta$ 7.87–7.81 (m, 2H, Ar-H), 7.69 (d, *J* = 16.5 Hz, 1H, Ph-CH=C), 7.54–7.44 (m, 6H, Ar-H), 7.41 (d, *J* = 8.6 Hz, 2H, Ar-H, Ph-C=CH), 5.27 (s, 2H, $-SCH_2-$). $^{13}C$ NMR (101 MHz, DMSO-$d_6$, ppm) $\delta$ 166.66, 160.69, 142.74, 134.78, 134.55, 133.67, 131.23, 129.53, 129.32, 128.82, 125.71, 109.28, 60.21. HRMS (ESI) $[M+H]^+$ calcd for $C_{17}H_{13}ClN_2O_3S$: 361.0400, found: 361.0408.

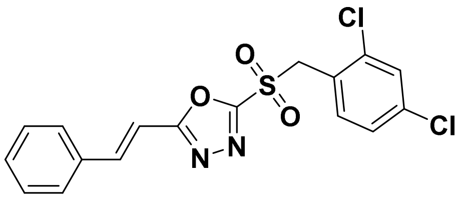

**5ac**

Data for (*E*)-2-((2,4-dichlorobenzyl)sulfonyl)-5-styryl-1,3,4-oxadiazole (**5ac**). White solid; yield 53.2%; m.p. 136–137 °C; $^1$H NMR (400 MHz, DMSO-$d_6$, ppm) $\delta$ 7.84 (dd, $J$ = 6.6, 2.8 Hz, 2H, Ar-H), 7.72 (dd, $J$ = 21.3, 3.7 Hz, 2H, Ar-H, Ph-CH=C), 7.62–7.55 (m, 2H, Ar-H), 7.52–7.46 (m, 4H, Ar-H, Ph-C=CH), 5.33 (s, 2H, -SCH$_2$-). $^{13}$C NMR (100 MHz, DMSO-$d_6$, ppm) $\delta$ 166.97, 160.44, 142.84, 136.19, 135.81, 135.68, 134.55, 131.26, 129.80, 129.52, 128.87, 128.47, 124.35, 109.30, 58.24. HRMS (ESI) [M+H]$^+$ calcd for $C_{17}H_{12}Cl_2N_2O_3S$: 395.0013, found: 395.0018.

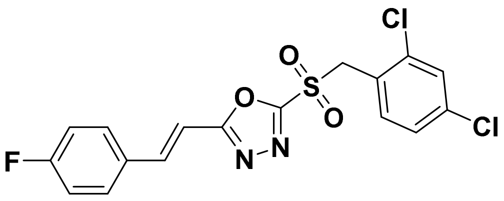

**5ad**

Data for (*E*)-2-((2,4-dichlorobenzyl)sulfonyl)-5-(4-fluorostyryl)-1,3,4-oxadiazole (**5ad**). White solid; yield 50.9%; m.p. 145–146 °C; $^1$H NMR (400 MHz, DMSO-$d_6$, ppm) $\delta$ 7.98–7.90 (m, 2H, Ar-H), 7.74 (dd, $J$ = 9.1, 7.4 Hz, 2H, Ar-H, Ph-CH=C), 7.62–7.55 (m, 2H, Ar-H), 7.47 (d, $J$ = 16.5 Hz, 1H, Ph-C=CH), 7.37–7.29 (m, 2H, Ar-H), 5.33 (s, 2H, -SCH$_2$-). $^{13}$C NMR (100 MHz, DMSO-$d_6$, ppm) $\delta$ 166.93, 163.93 (d, $J$ = 249.4 Hz), 160.40, 141.59, 136.17, 135.80, 135.62, 131.32, 131.26 (d, $J$ = 4.1 Hz), 129.80, 128.47, 124.33, 116.58 (d, $J$ = 21.9 Hz), 109.15 (d, $J$ = 2.3 Hz), 58.22. HRMS (ESI) [M+H]$^+$ calcd for $C_{17}H_{11}Cl_2FN_2O_3S$: 412.9918, found: 412.9924.

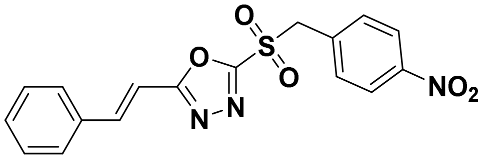

**5ae**

Data for (*E*)-2-((4-nitrobenzyl)sulfonyl)-5-styryl-1,3,4-oxadiazole (**5ae**). White solid; yield 63.7%; m.p. 156–157 °C; $^1$H NMR (400 MHz, DMSO-$d_6$, ppm) $\delta$ 8.26 (d, $J$ = 7.9 Hz, 2H, Ar-H), 7.79 (m, $J$ = 3.0 Hz, 2H, Ar-H), 7.71–7.60 (m, 3H, Ar-H, Ph-CH=C), 7.45 (d, $J$ = 9.6 Hz, 4H, Ar-H, Ph-C=CH), 5.43 (s, 2H, -SCH$_2$-). $^{13}$C NMR (100 MHz, DMSO-$d_6$, ppm) $\delta$ 166.79, 160.63, 148.58, 142.87, 134.61, 134.30, 133.40, 131.33, 129.60, 128.90, 124.29, 109.36, 60.16. HRMS (ESI) [M+H]$^+$ calcd for $C_{17}H_{13}N_3O_5S$: 372.0648, found: 372.0649.

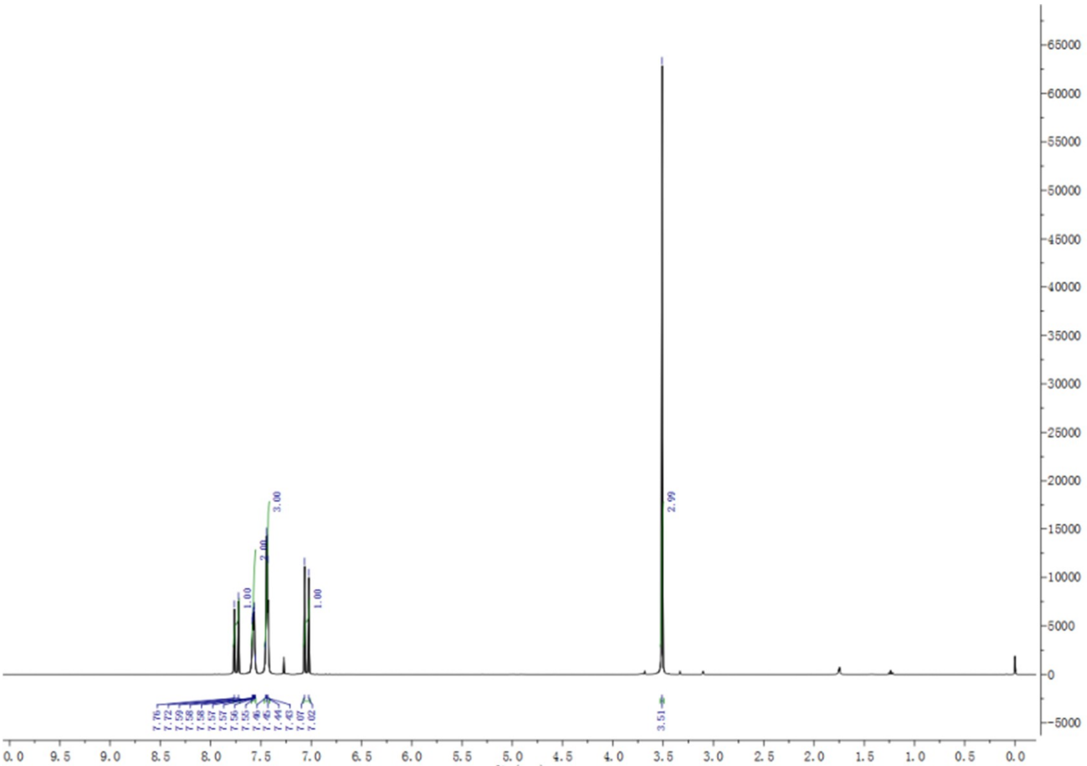


**¹H NMR for compound 5a**

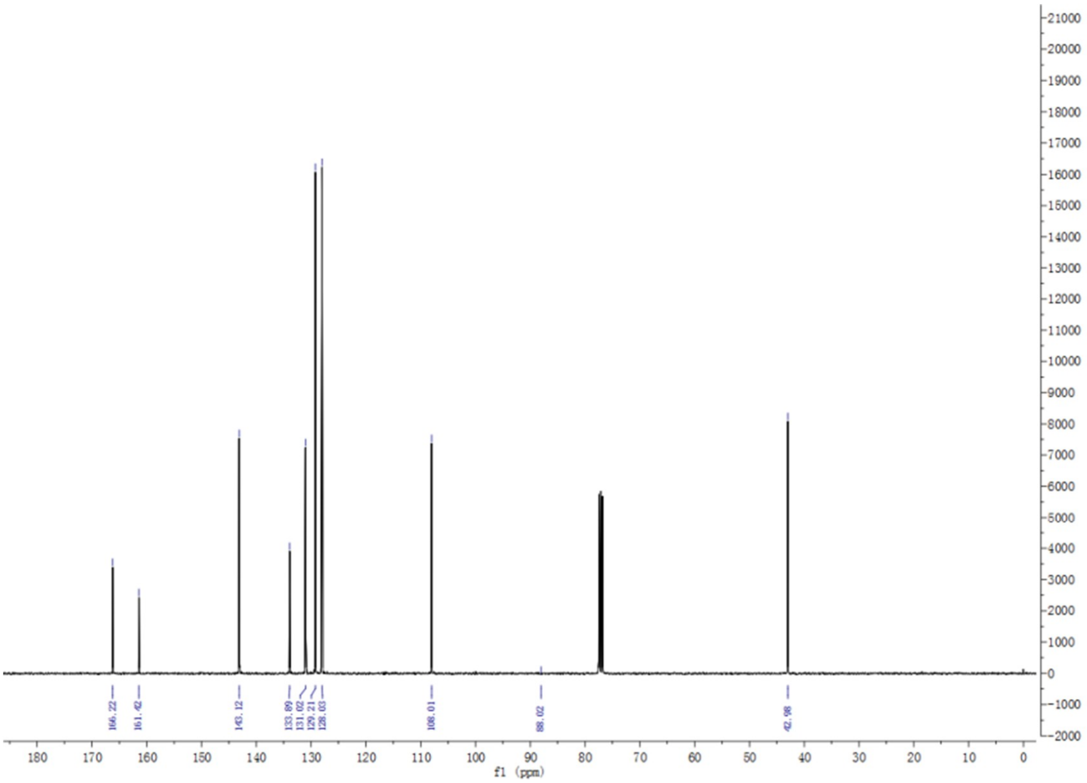


**¹³C NMR of compound 5a**

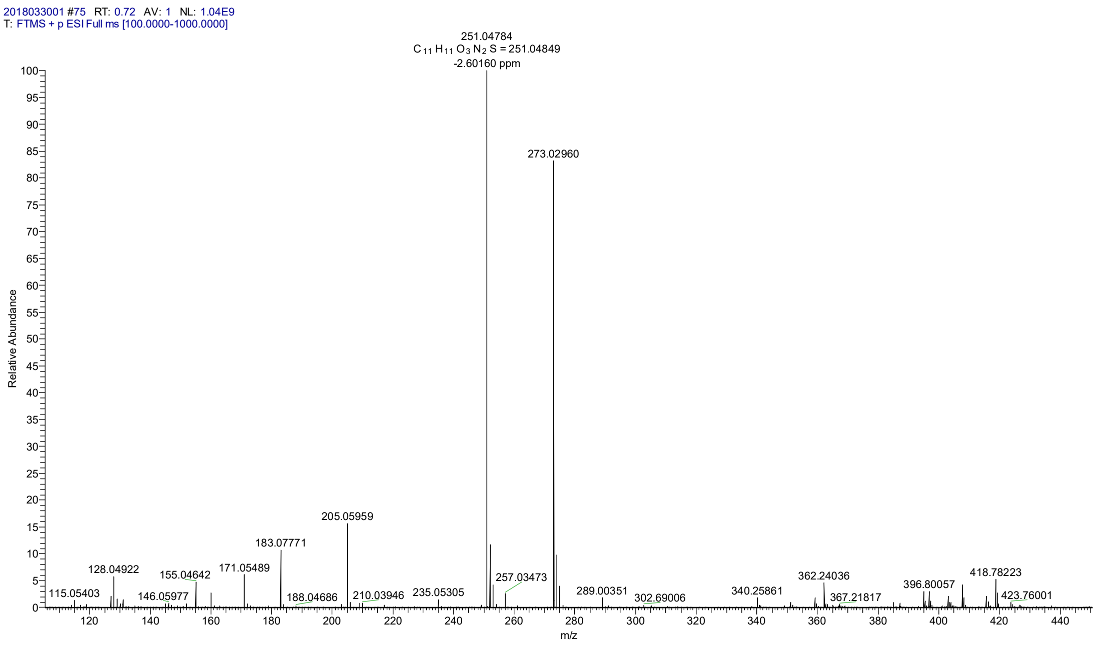


**HRMS (ESI) for compound 5a**

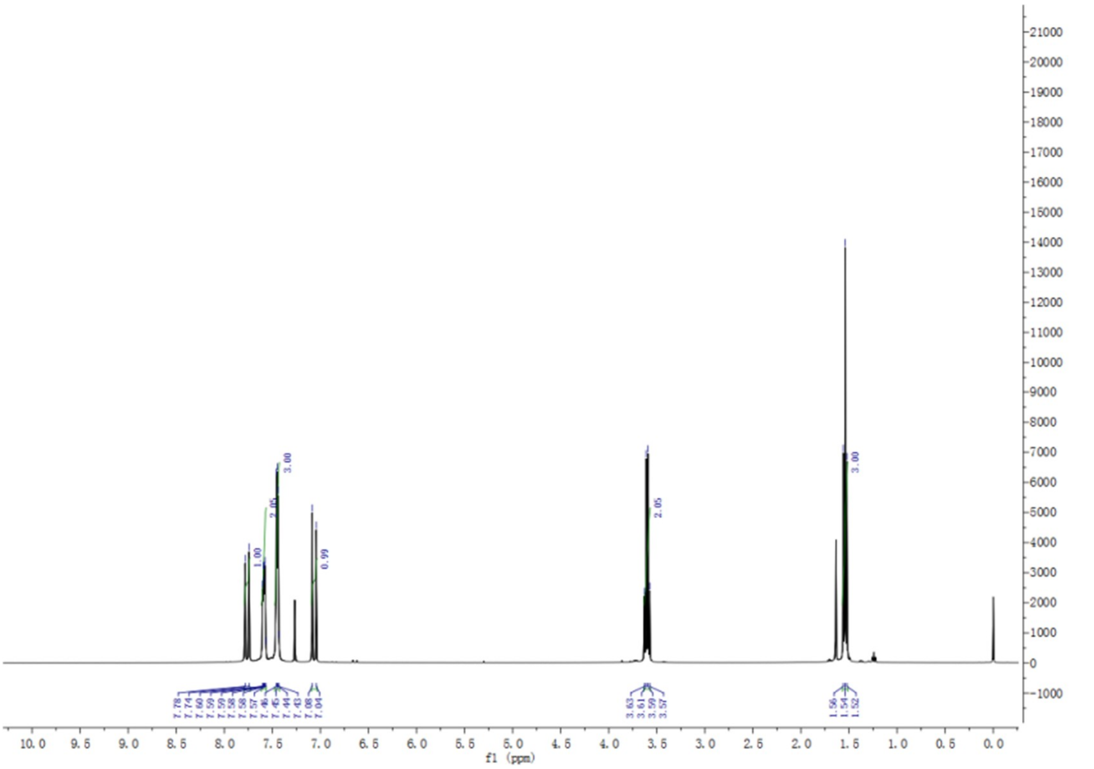


**$^{1}$H NMR for compound 5b**

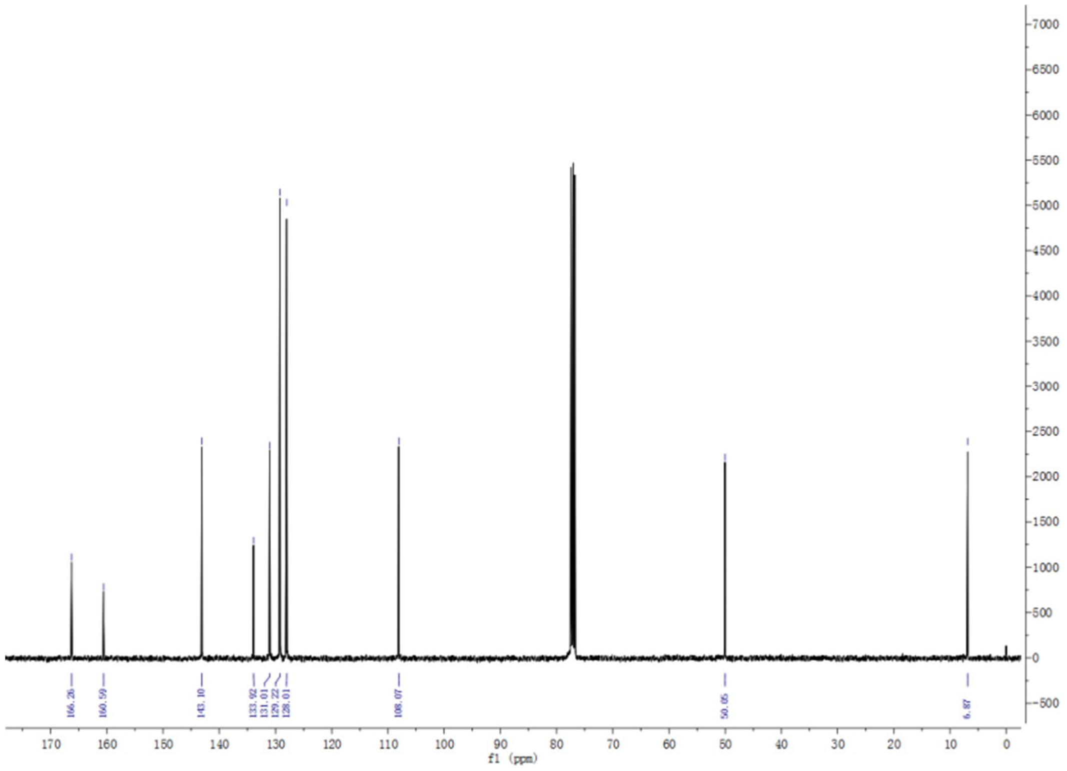


**$^{13}C$ NMR of compound 5b**

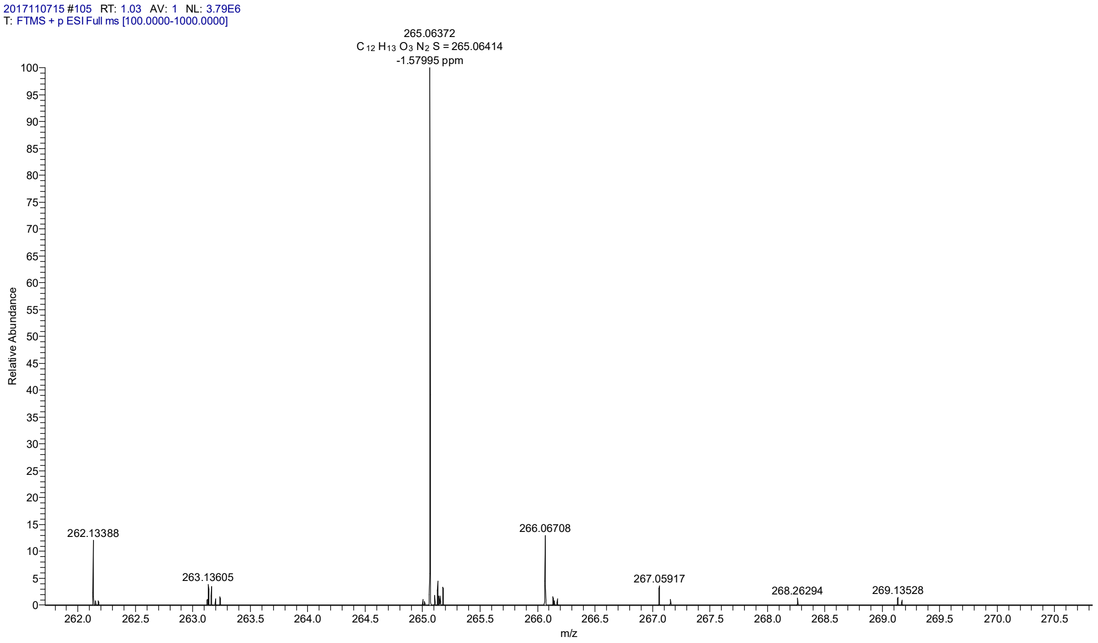


**HRMS (ESI) for compound 5b**

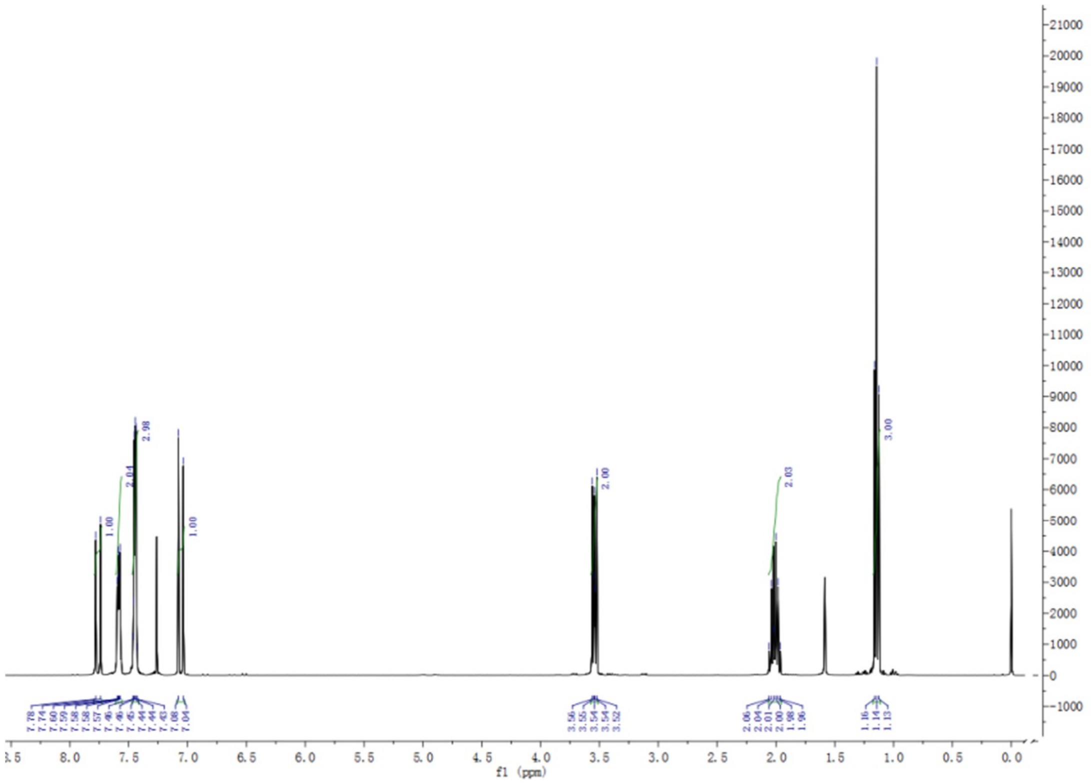


¹H NMR for compound 5c

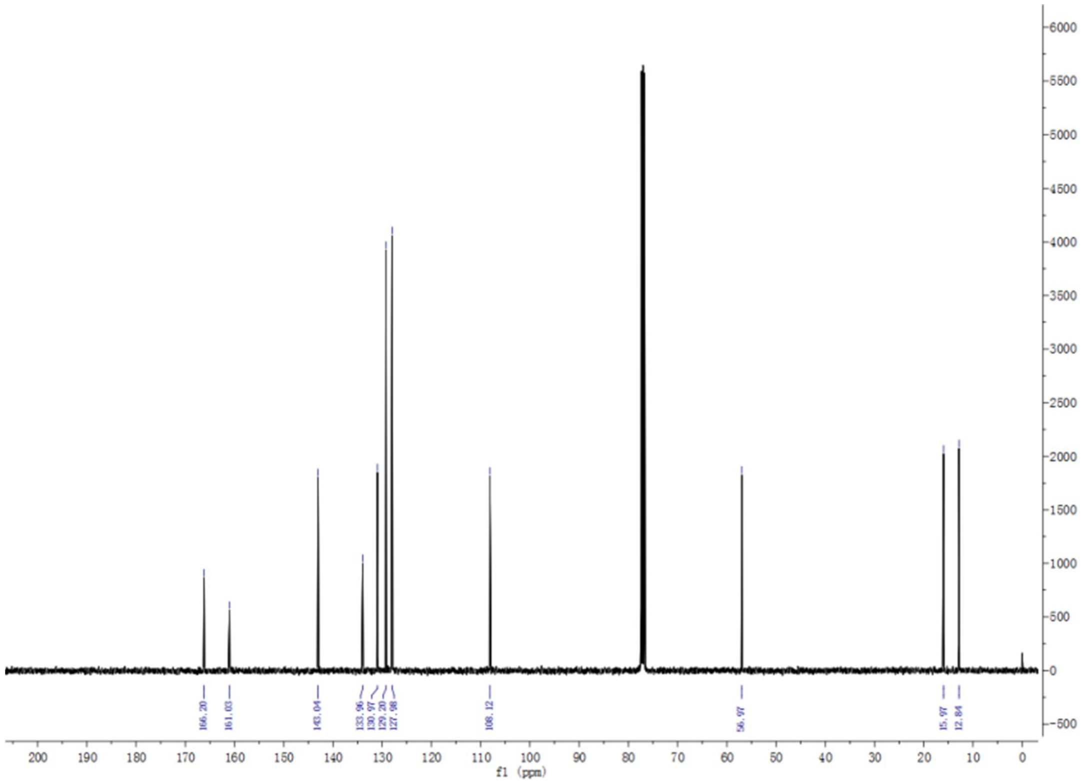


¹³C NMR of compound 5c

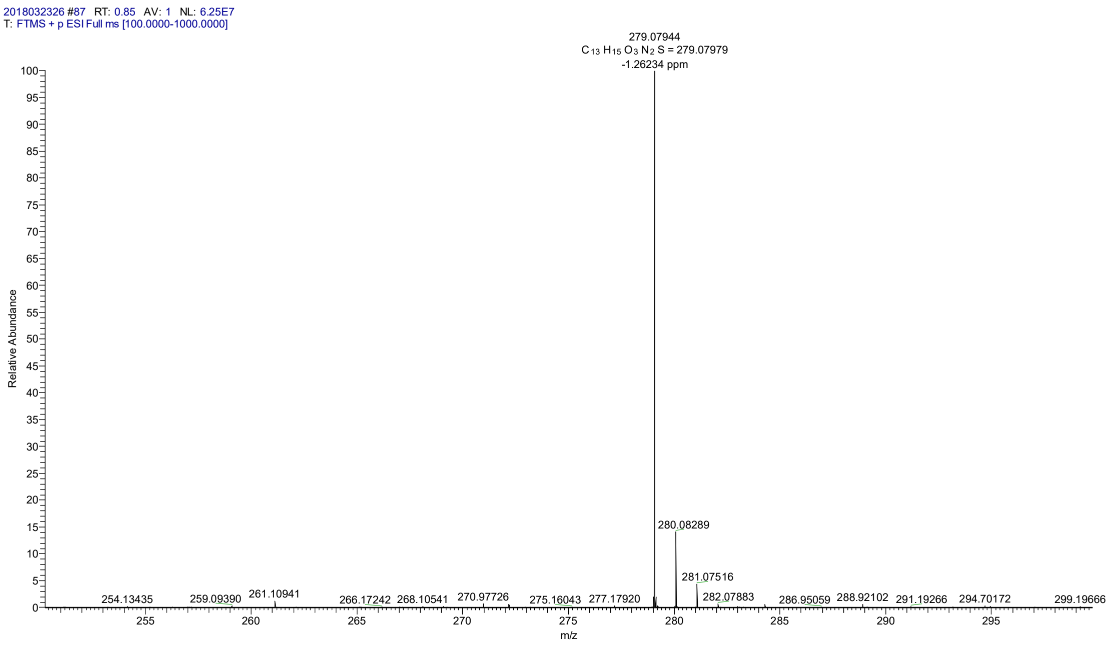


**HRMS (ESI) for compound 5c**

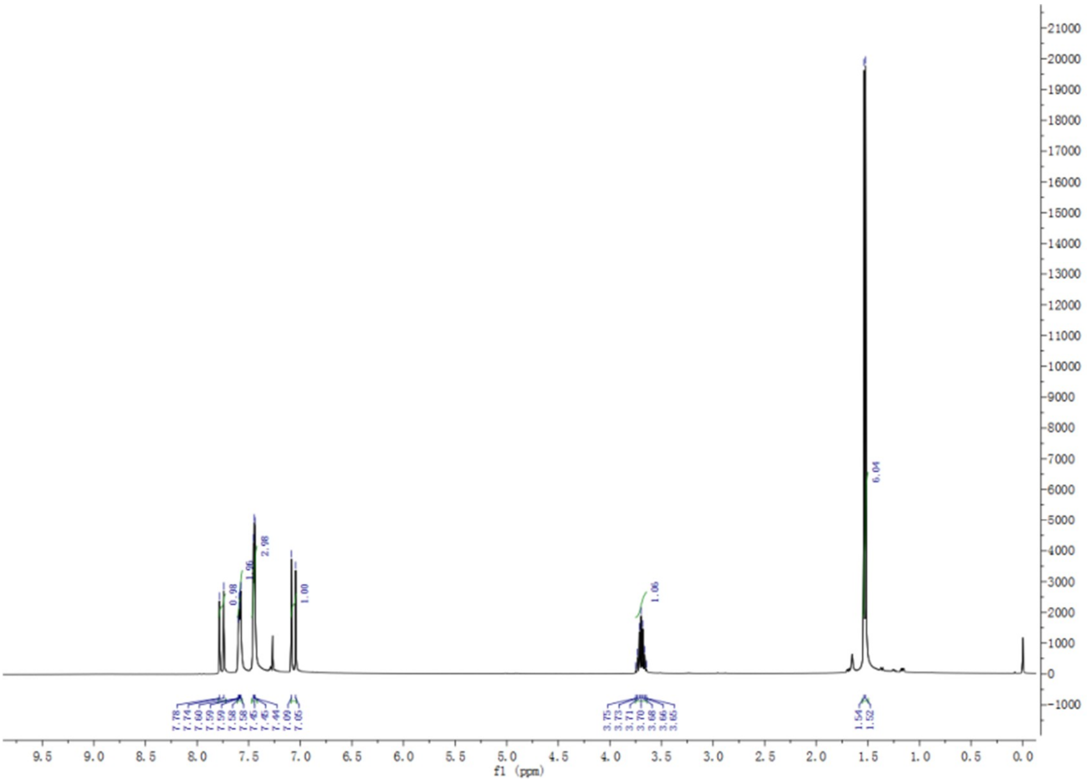


**$^1$H NMR for compound 5d**

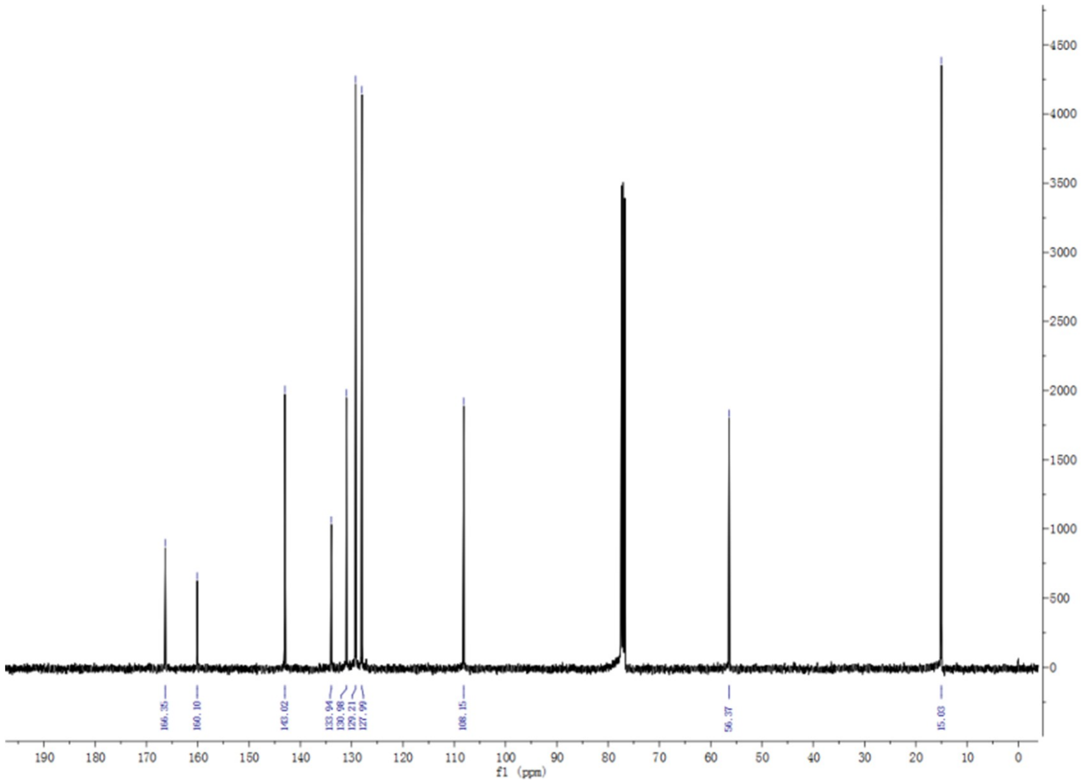


**$^{13}C$ NMR of compound 5d**

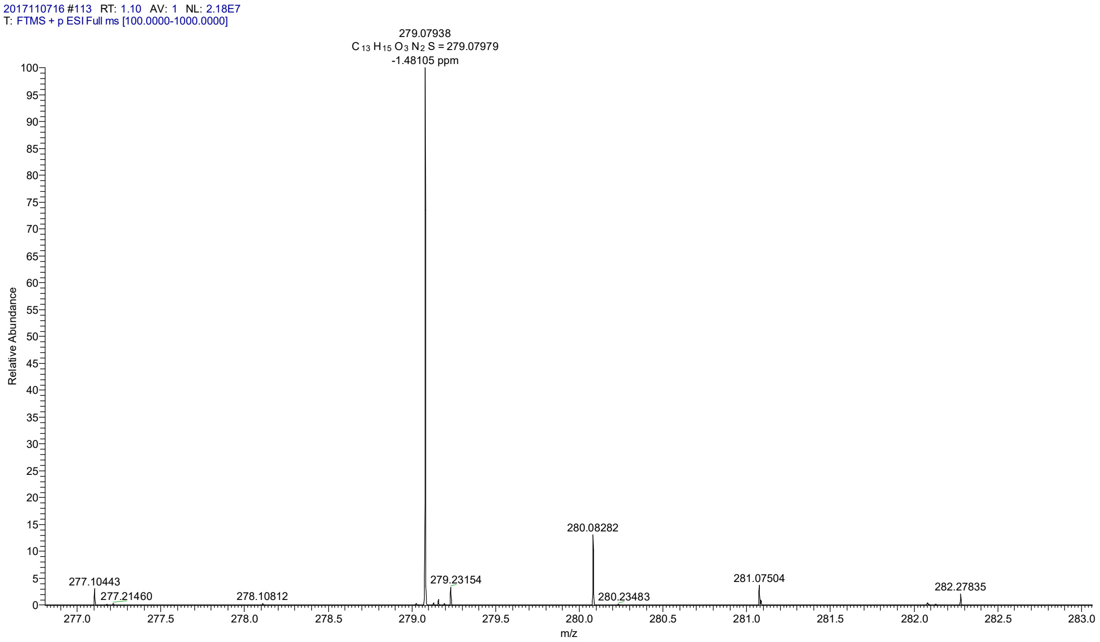


**HRMS (ESI) for compound 5d**

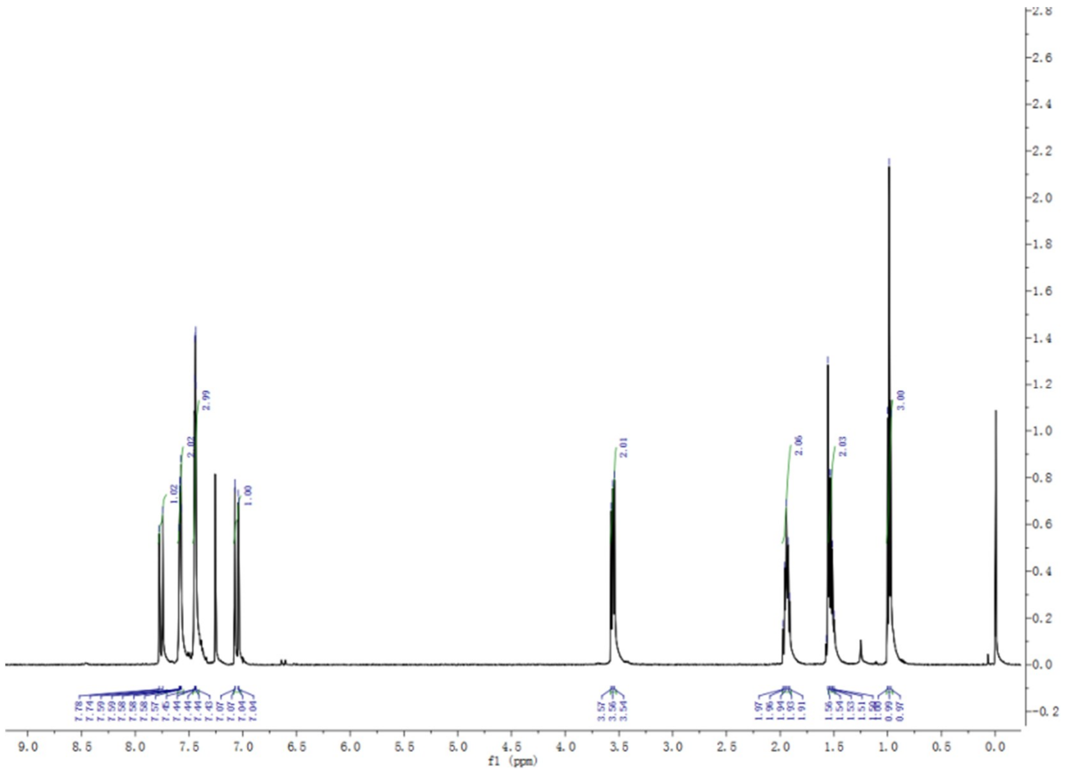


**$^{1}$H NMR for compound 5e**

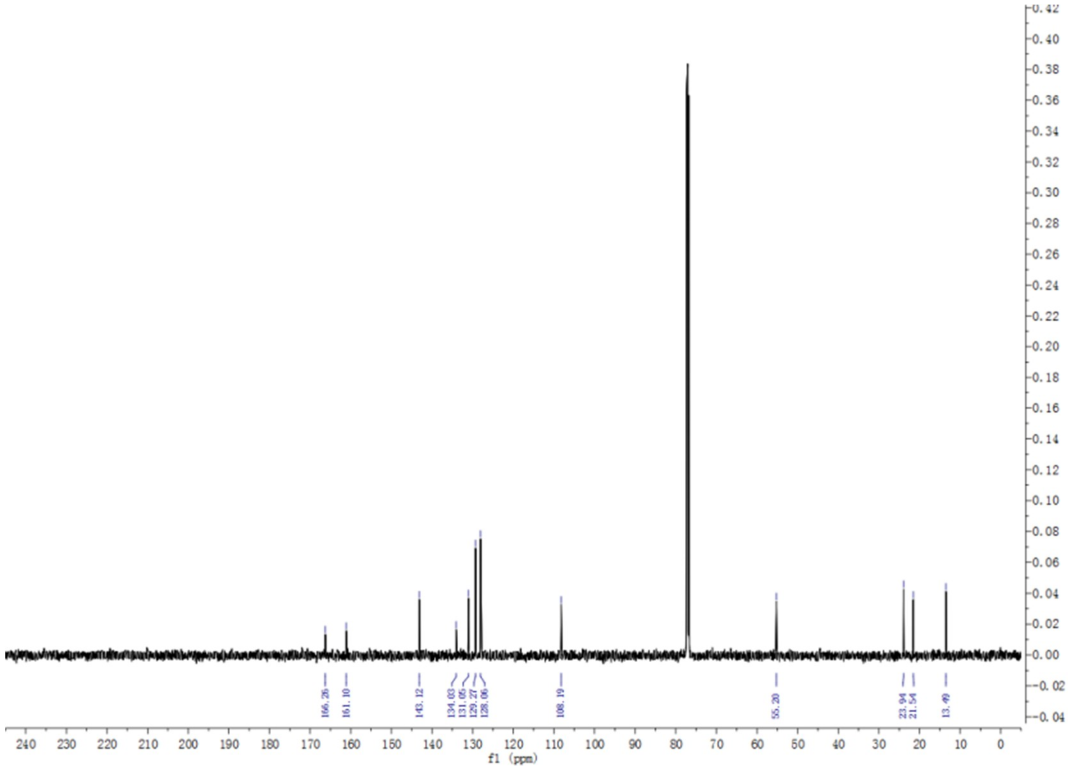


**$^{13}$C NMR of compound 5e**

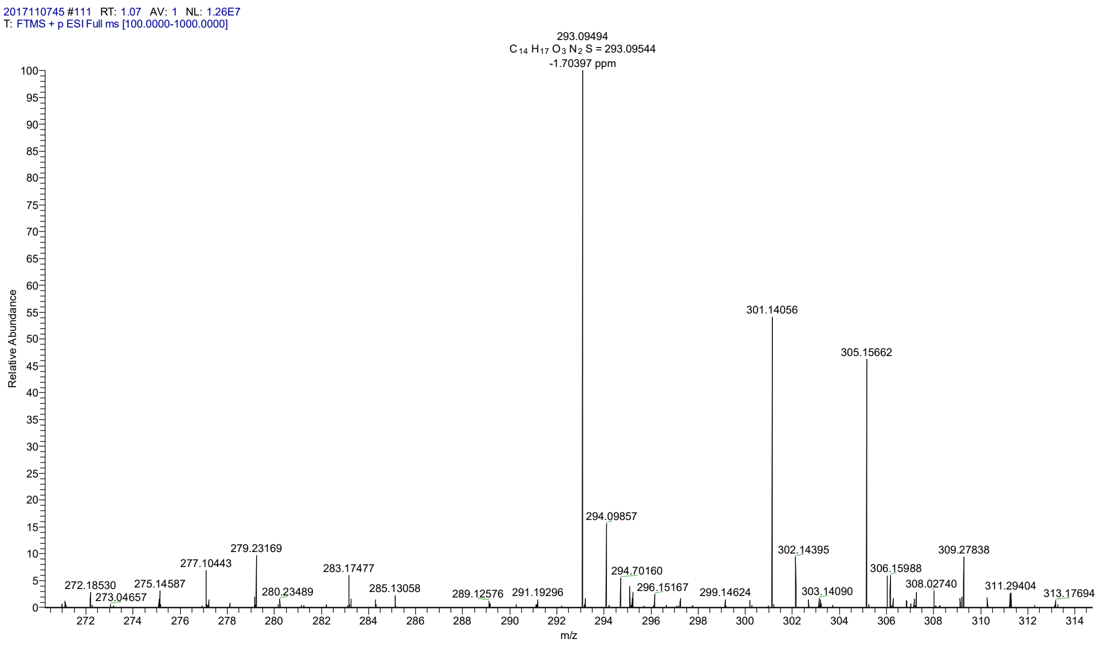


**HRMS (ESI) for compound 5e**

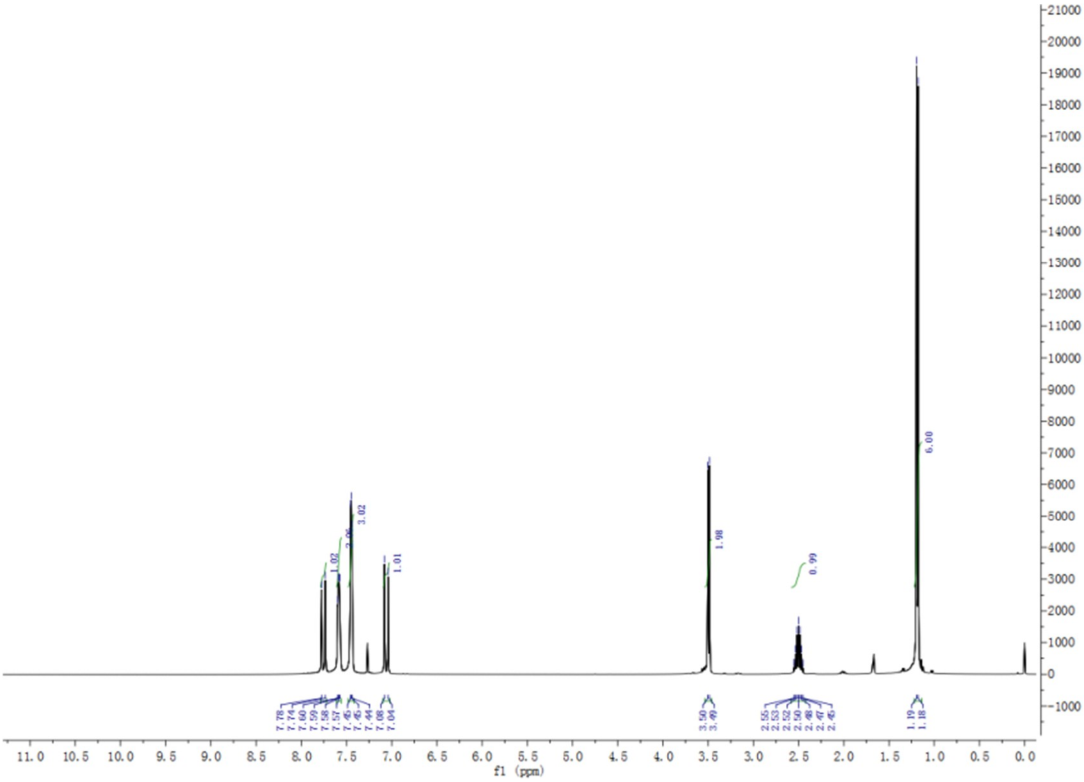


**$^1$H NMR for compound 5f**

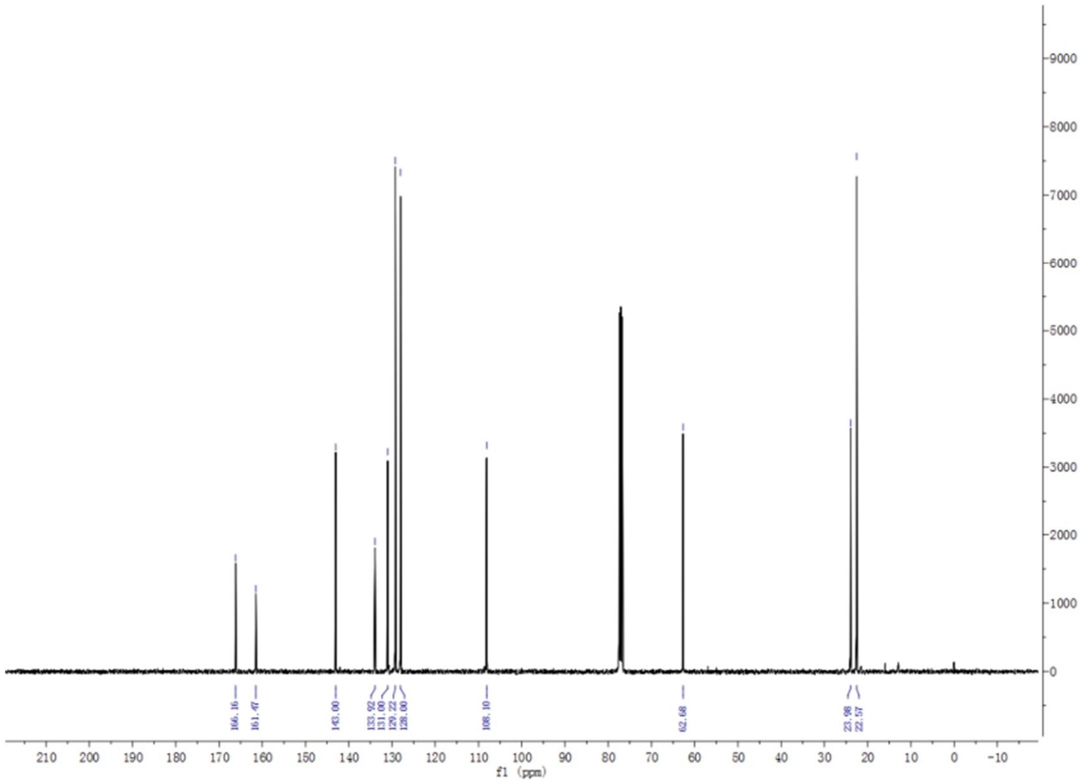


**$^{13}$C NMR of compound 5f**

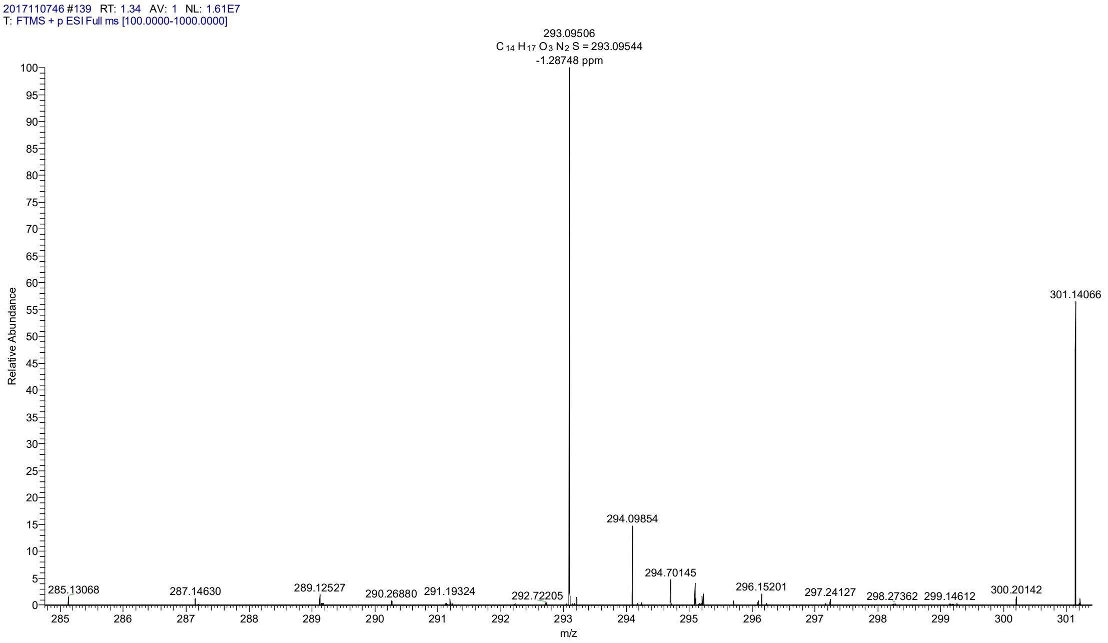


**HRMS (ESI) for compound 5f**

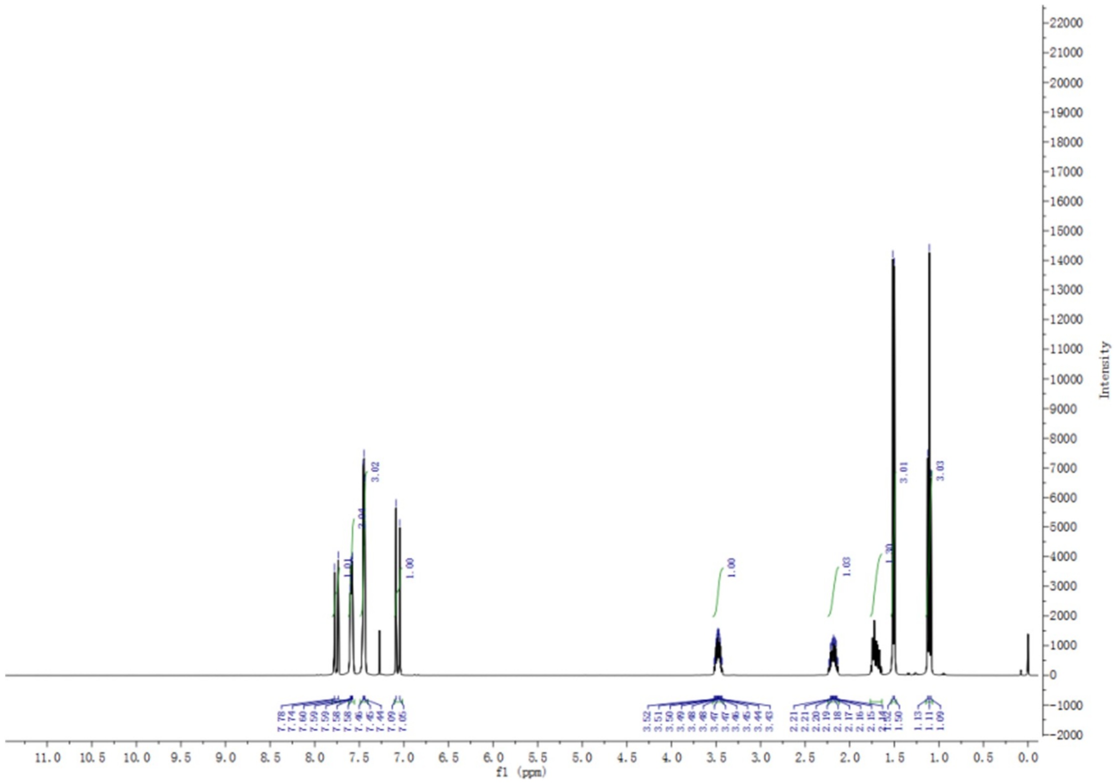


<sup></sup>

$^{1}$H NMR for compound 5g

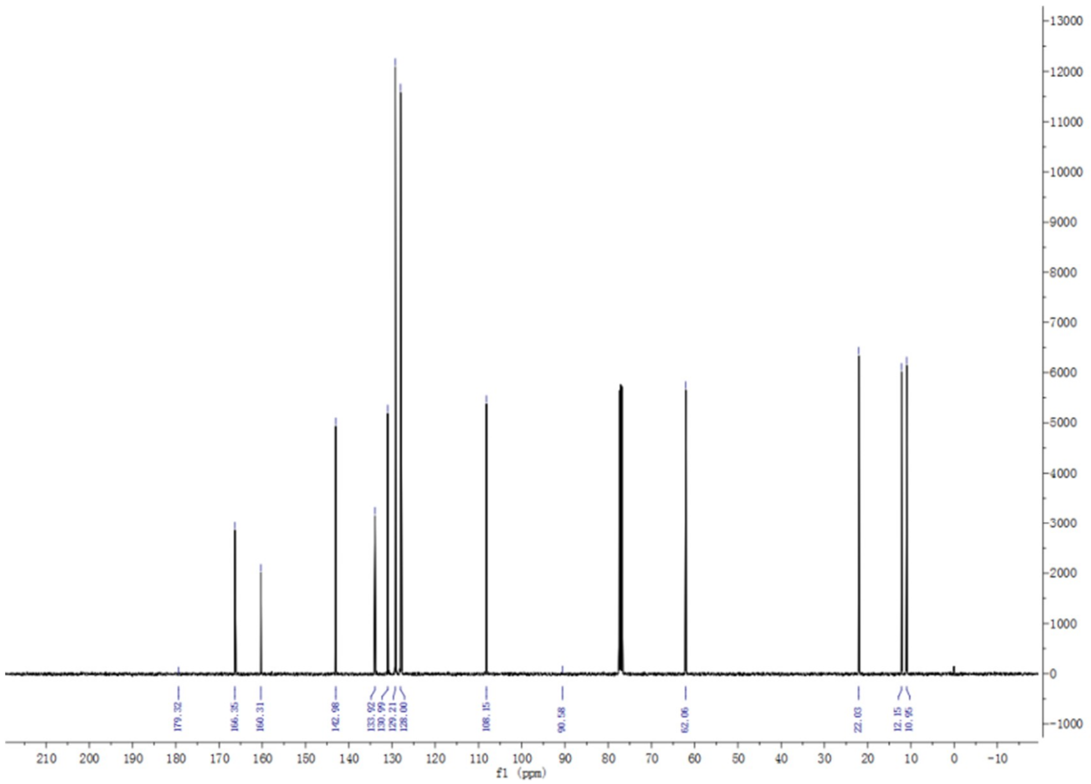


$^{13}$C NMR of compound 5g

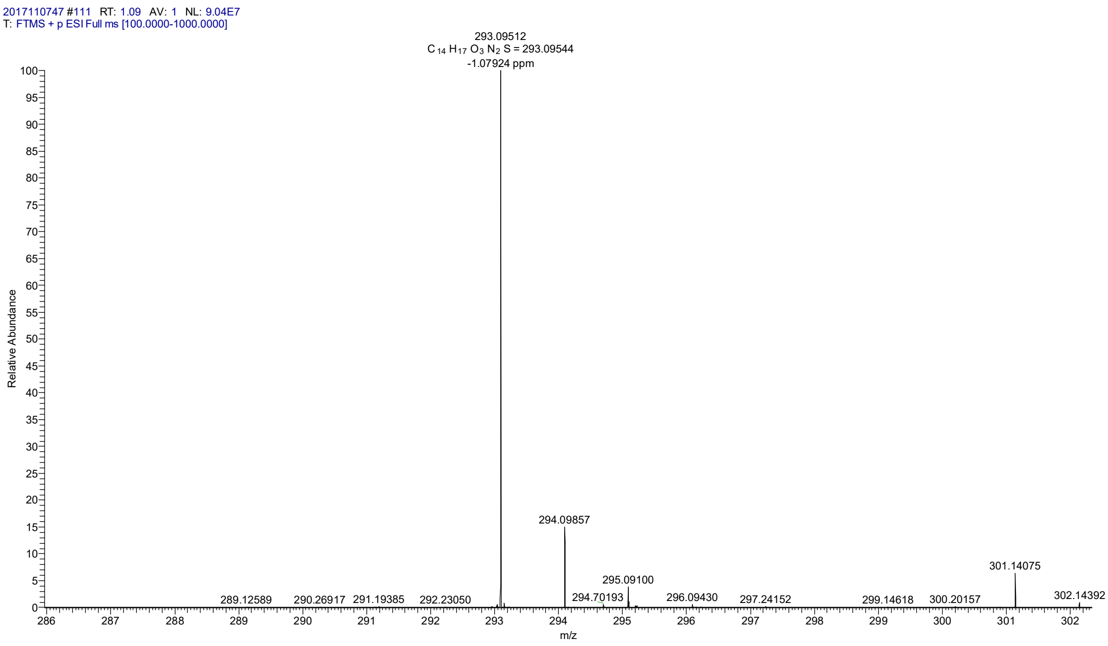


**HRMS (ESI) for compound 5g**

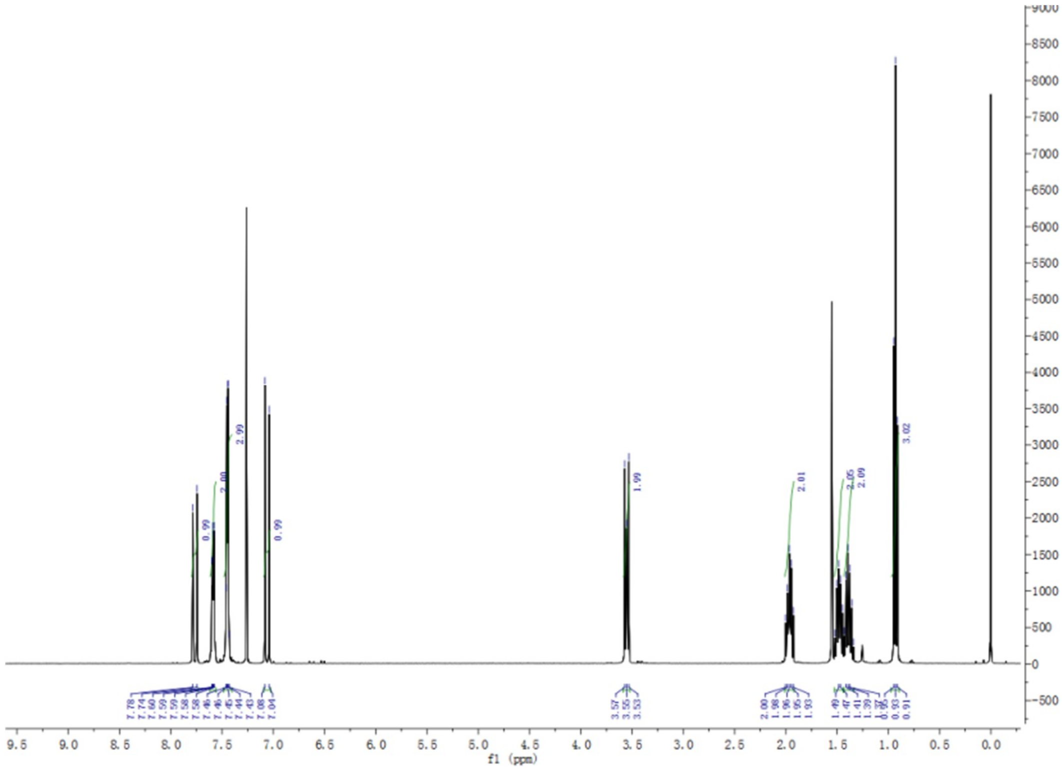


**$^{1}$H NMR for compound 5h**

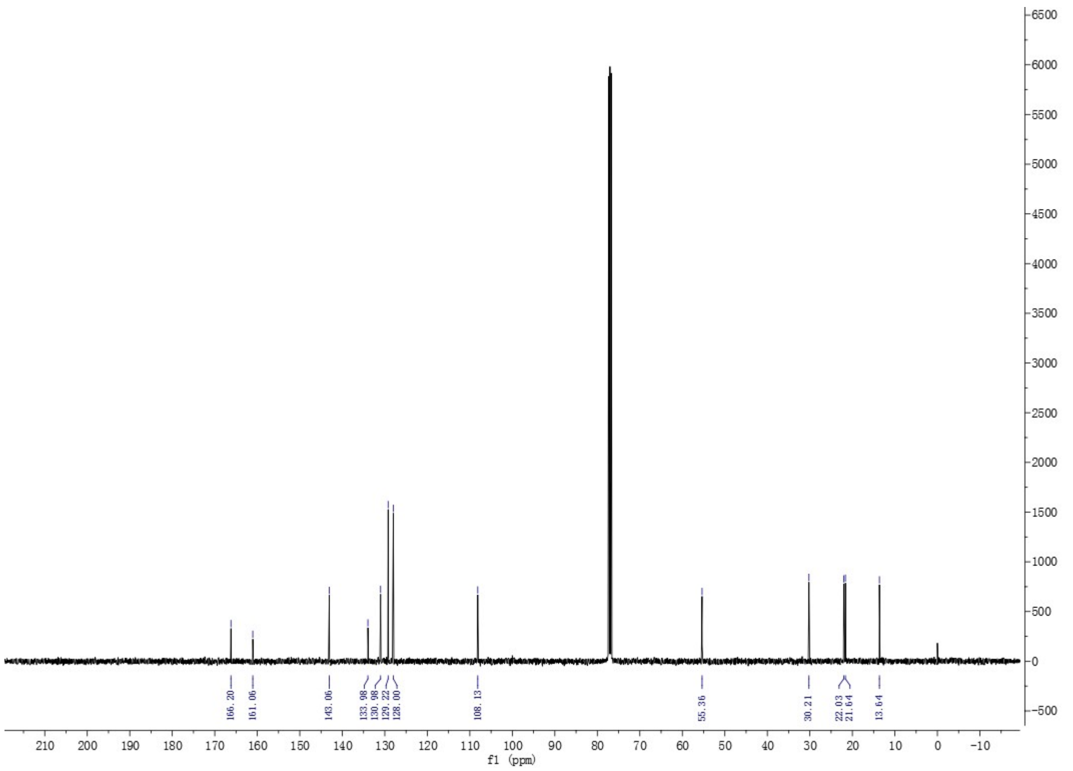


**¹³C NMR of compound 5h**

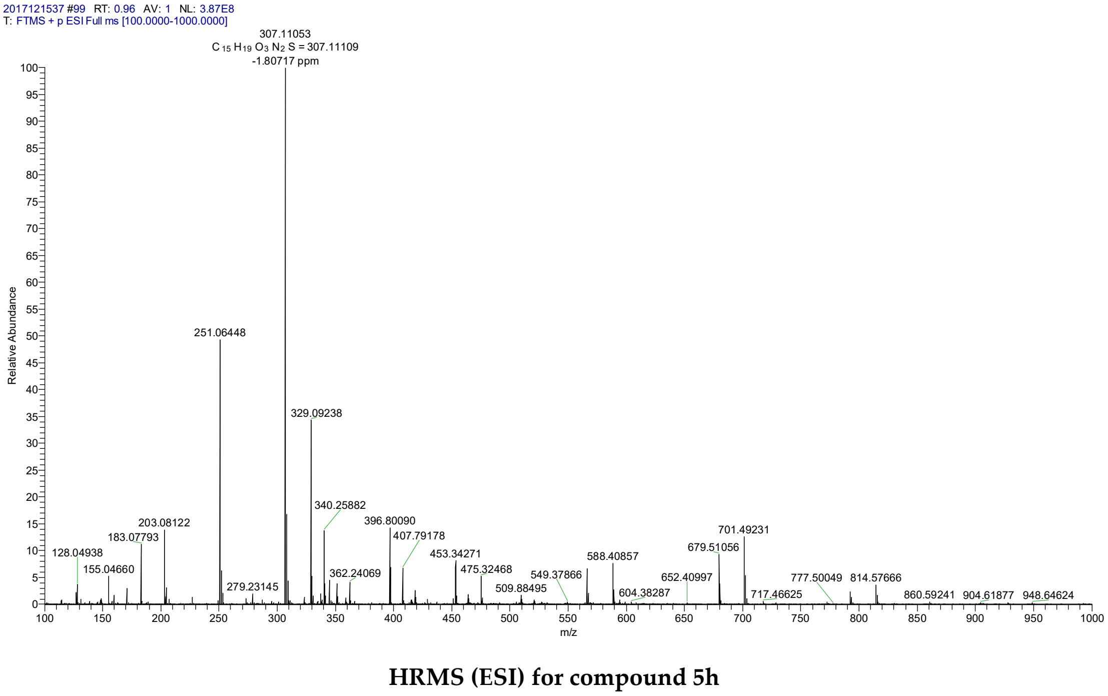


**HRMS (ESI) for compound 5h**

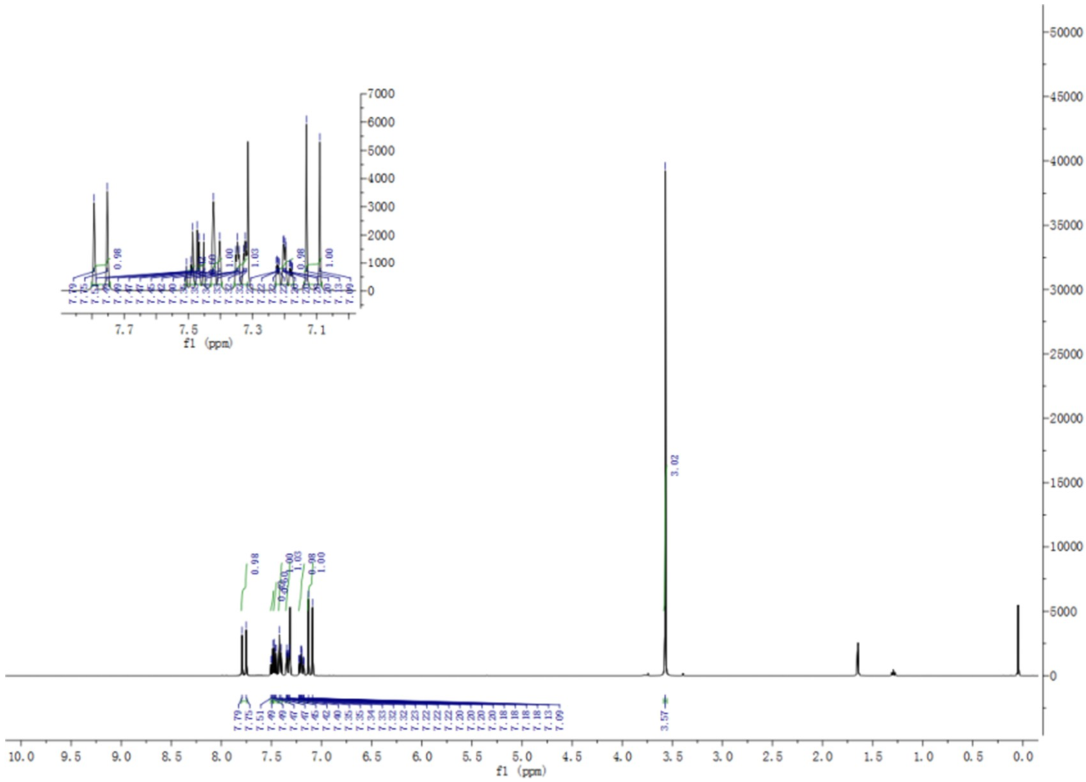


**$^{1}$H NMR for compound 5i**

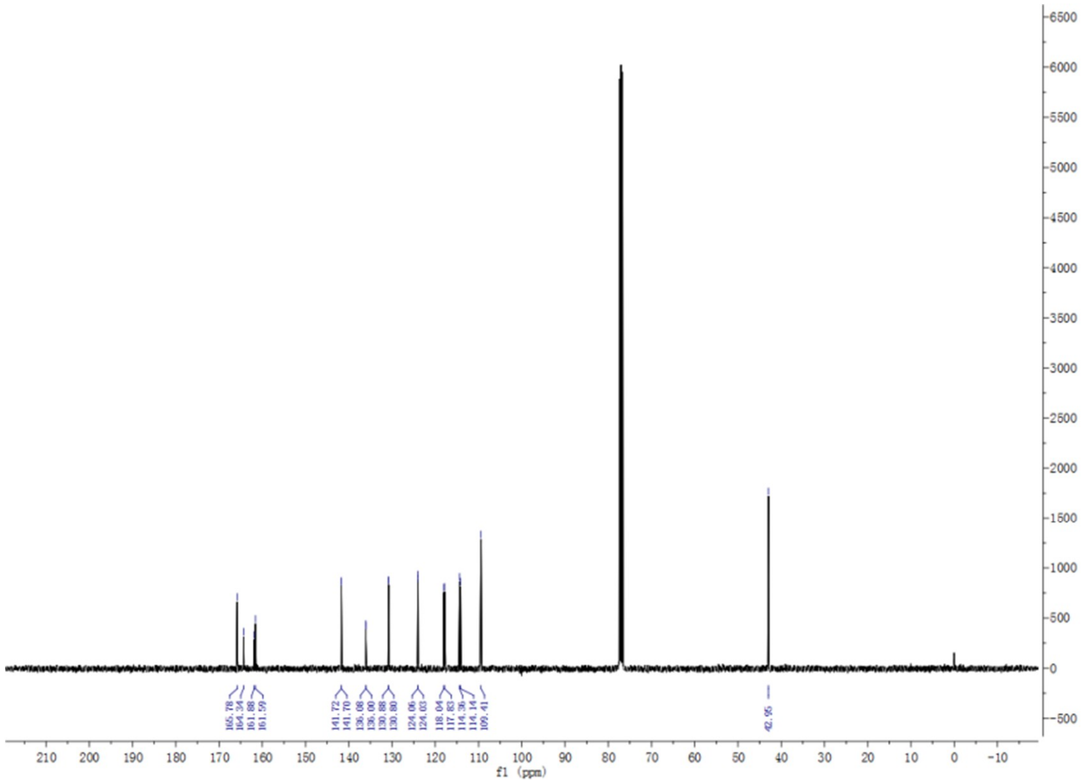


**$^{13}$C NMR of compound 5i**

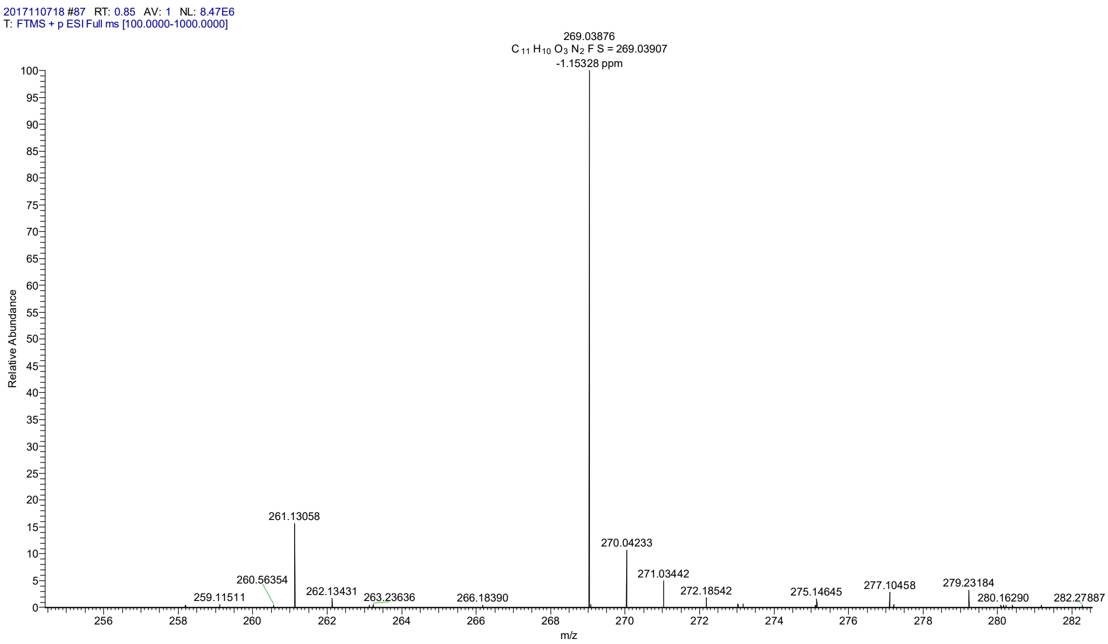


**HRMS (ESI) for compound 5i**

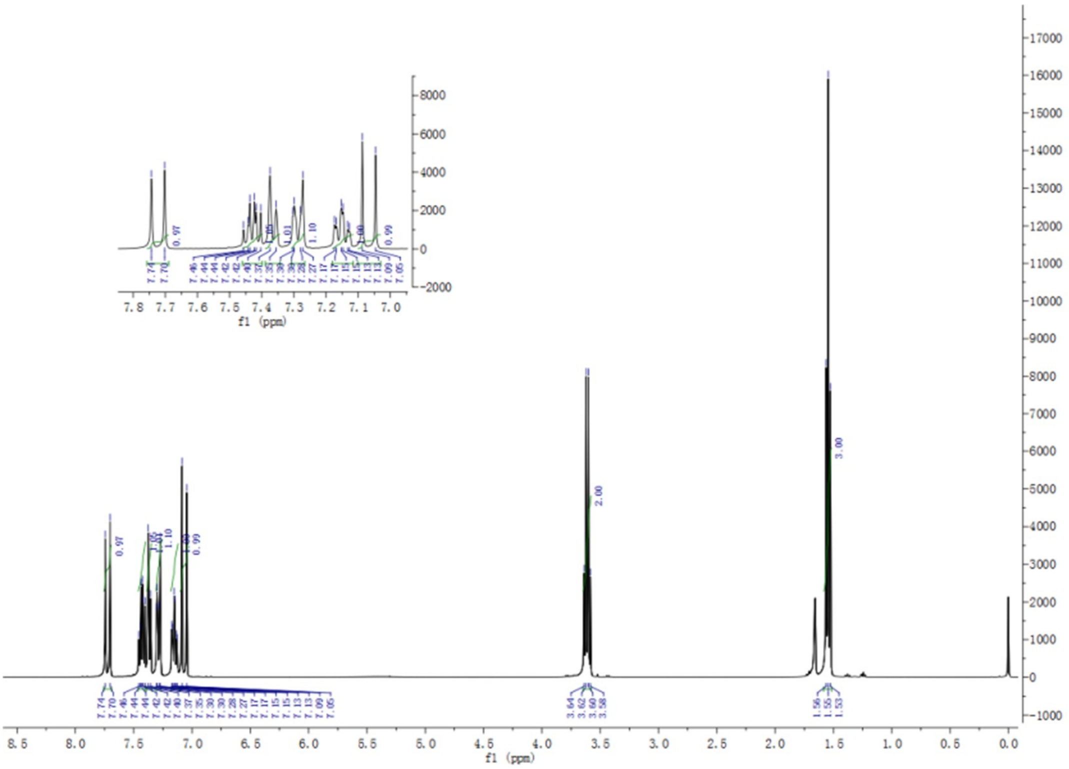


**¹H NMR for compound 5j**

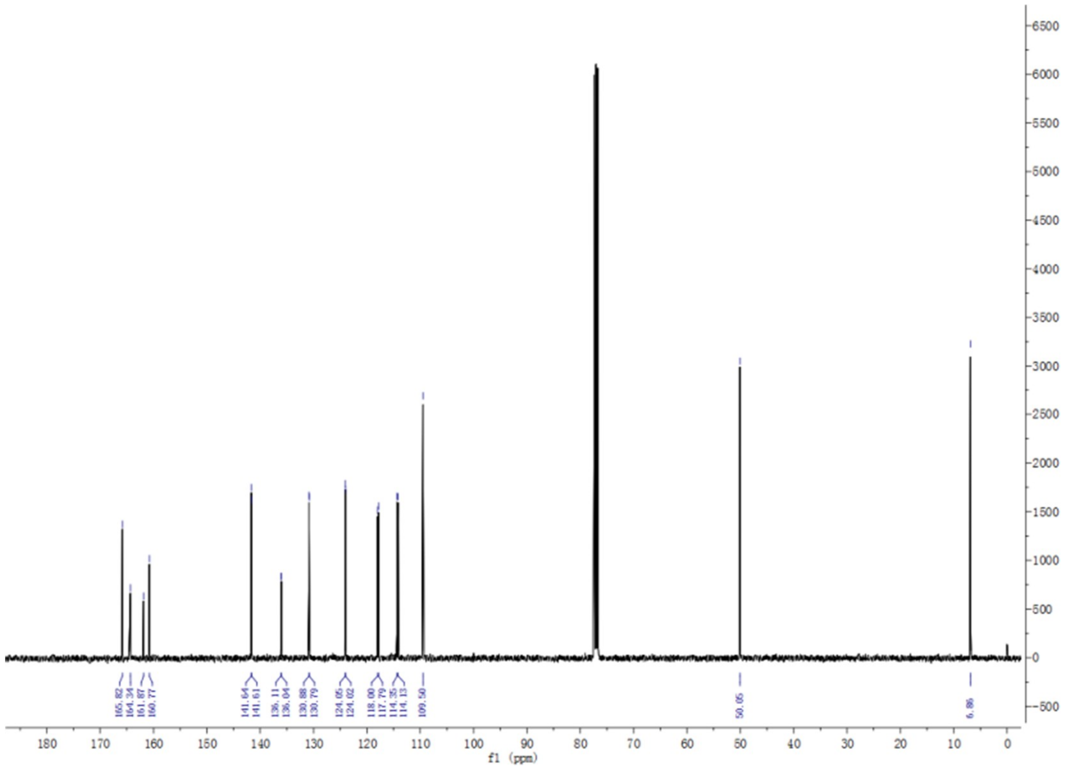


**$^{13}C$ NMR of compound 5j**

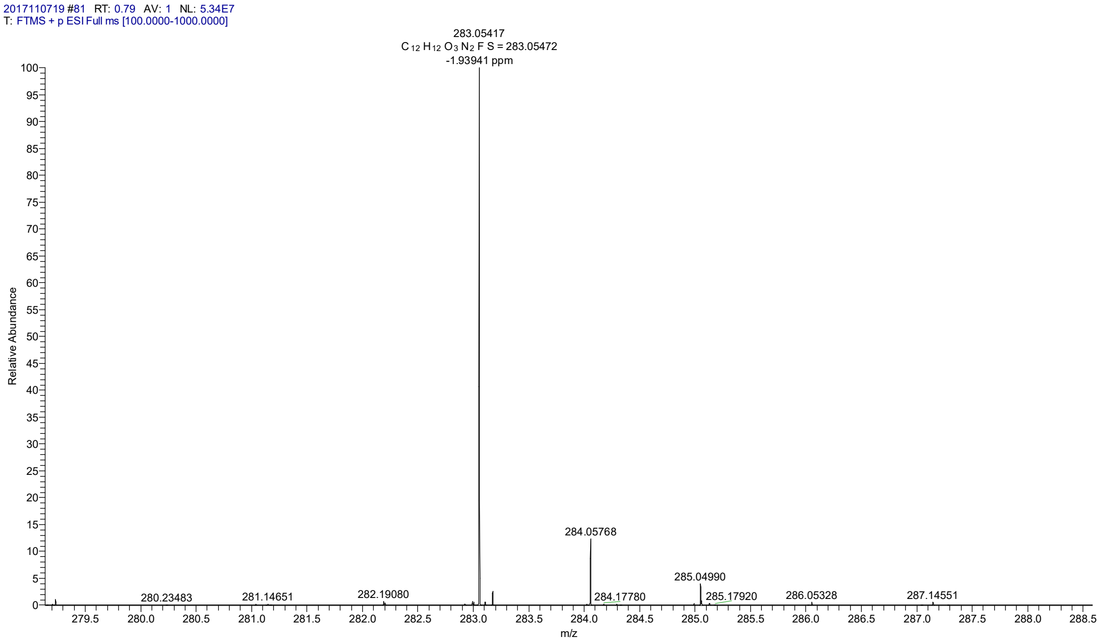


**HRMS (ESI) for compound 5j**

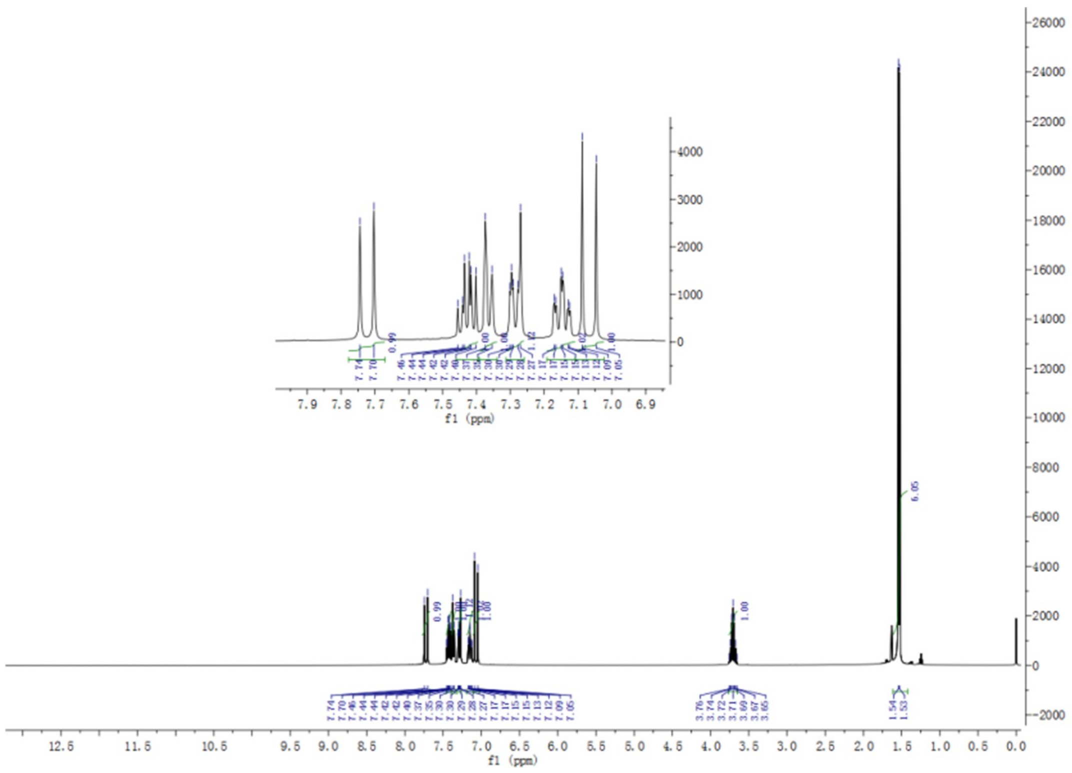

**$^1$H NMR for compound 5k**

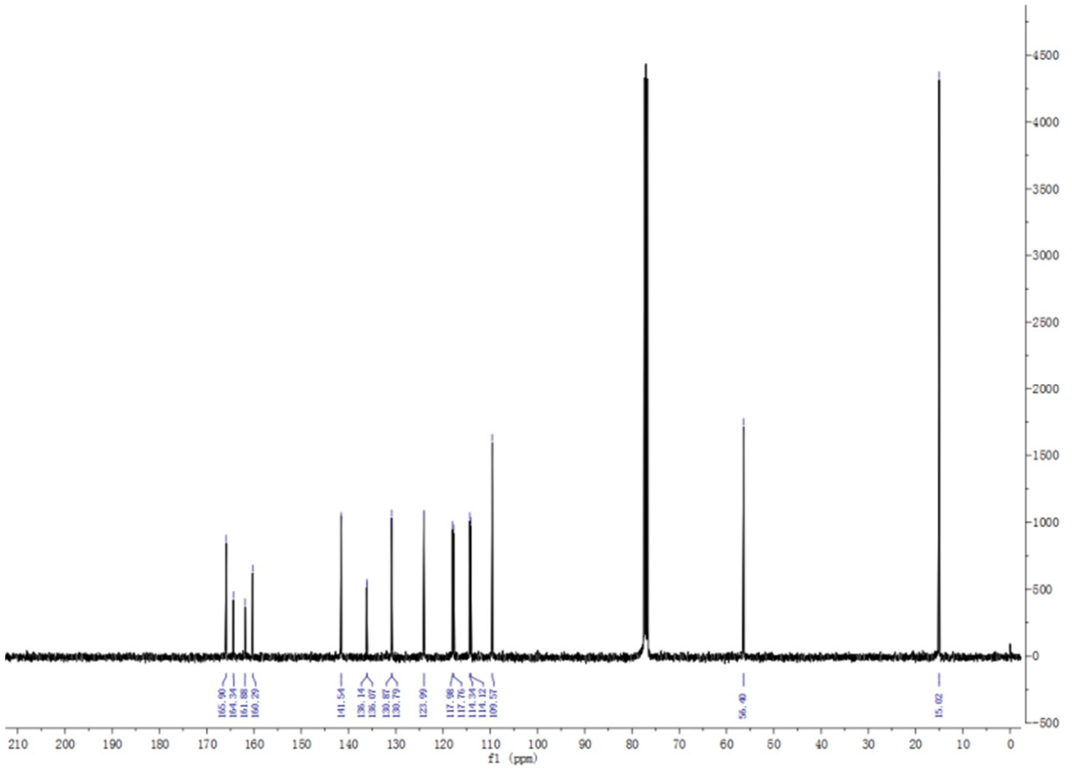

**$^{13}$C NMR of compound 5k**

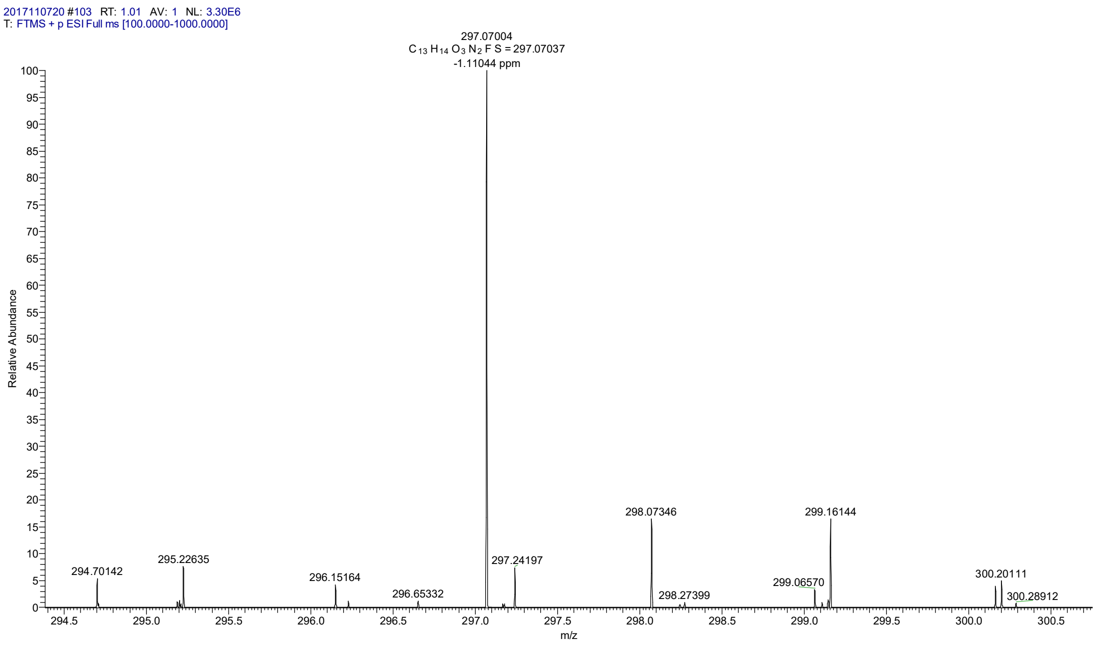


**HRMS (ESI) for compound 5k**

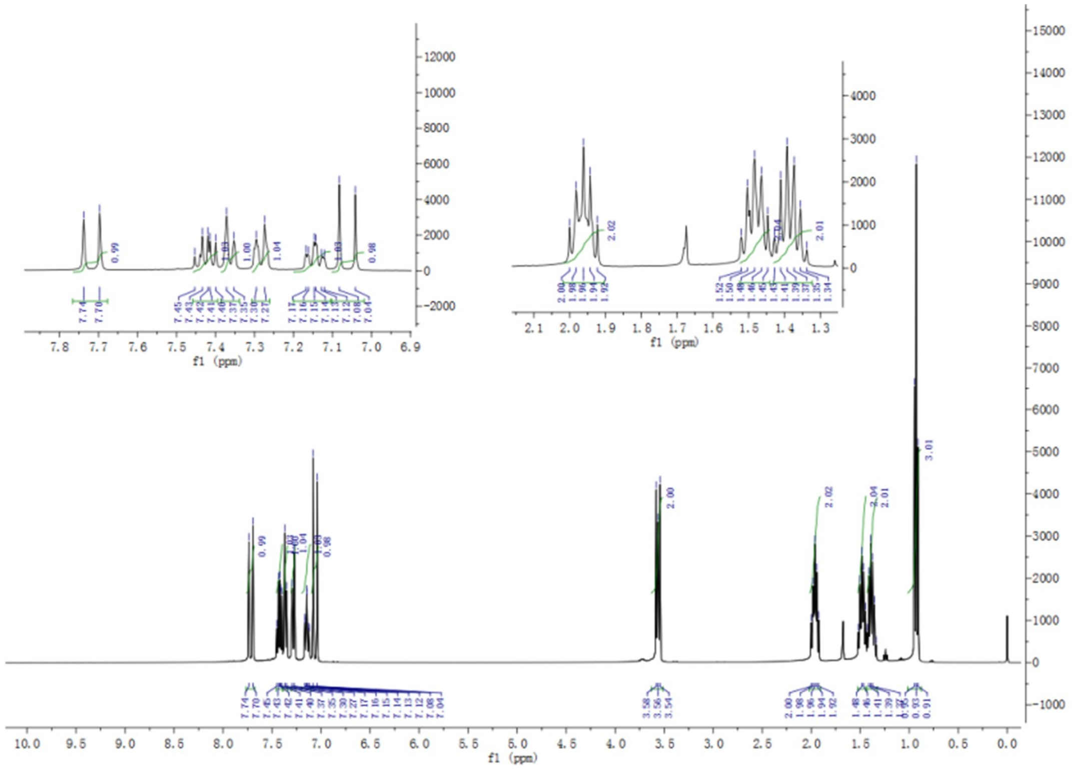


**$^{1}$H NMR for compound 5l**

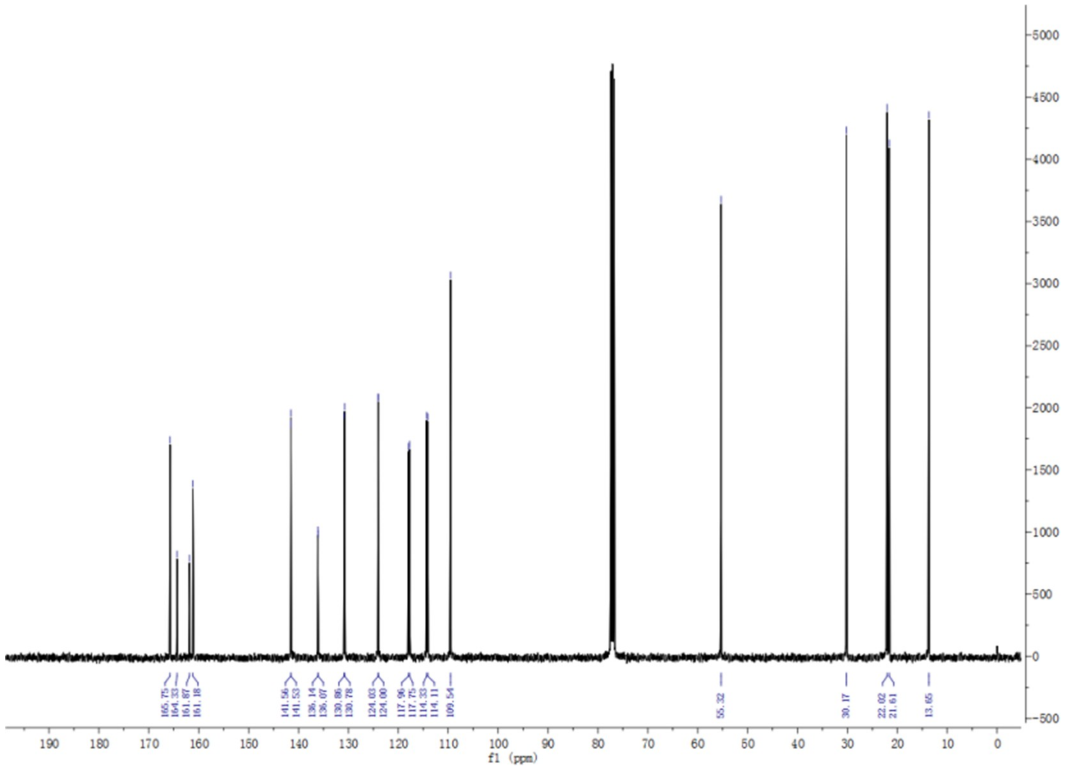


**$^{13}$C NMR of compound 5l**

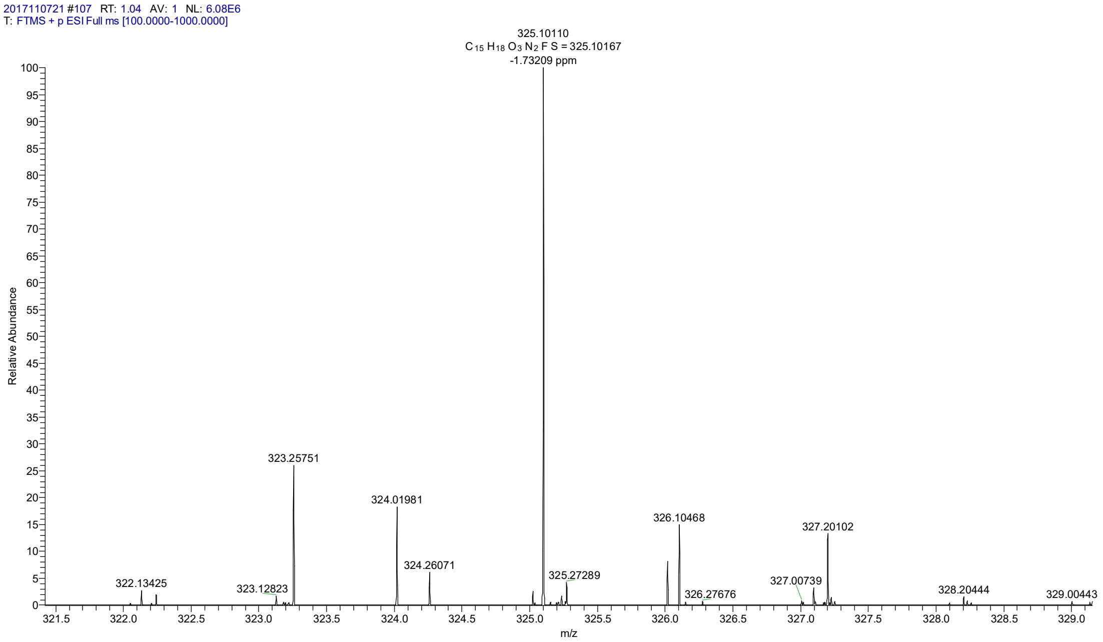


**HRMS (ESI) for compound 5l**

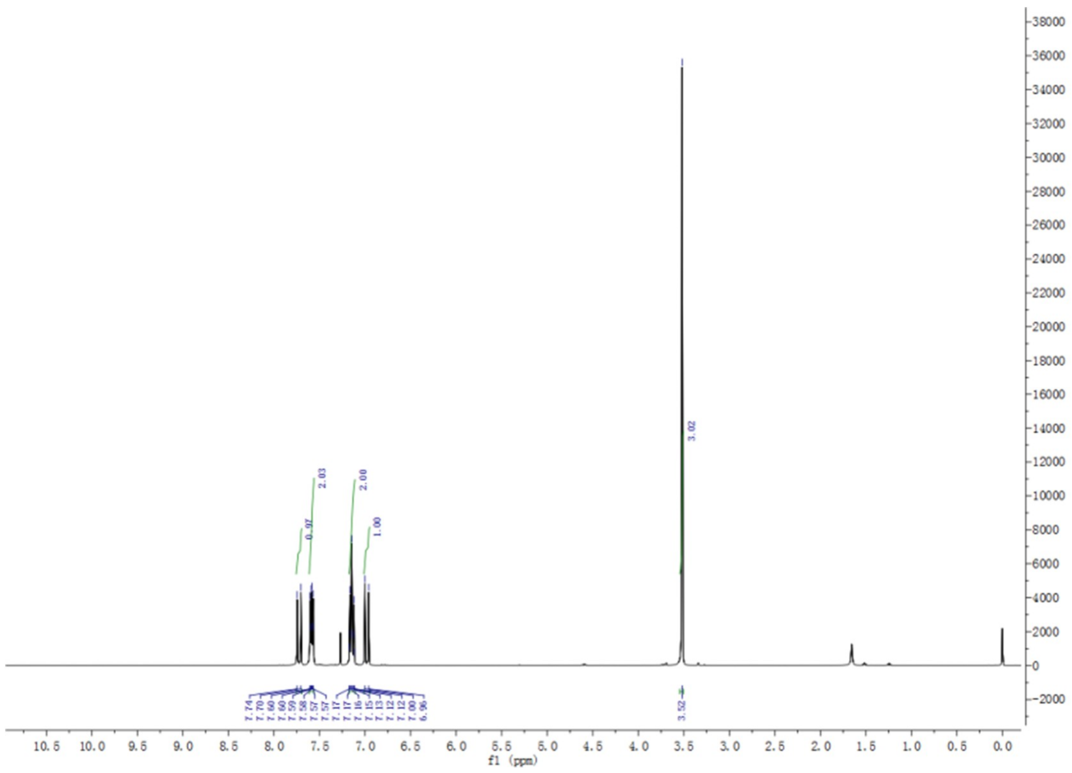

¹H NMR for compound 5m

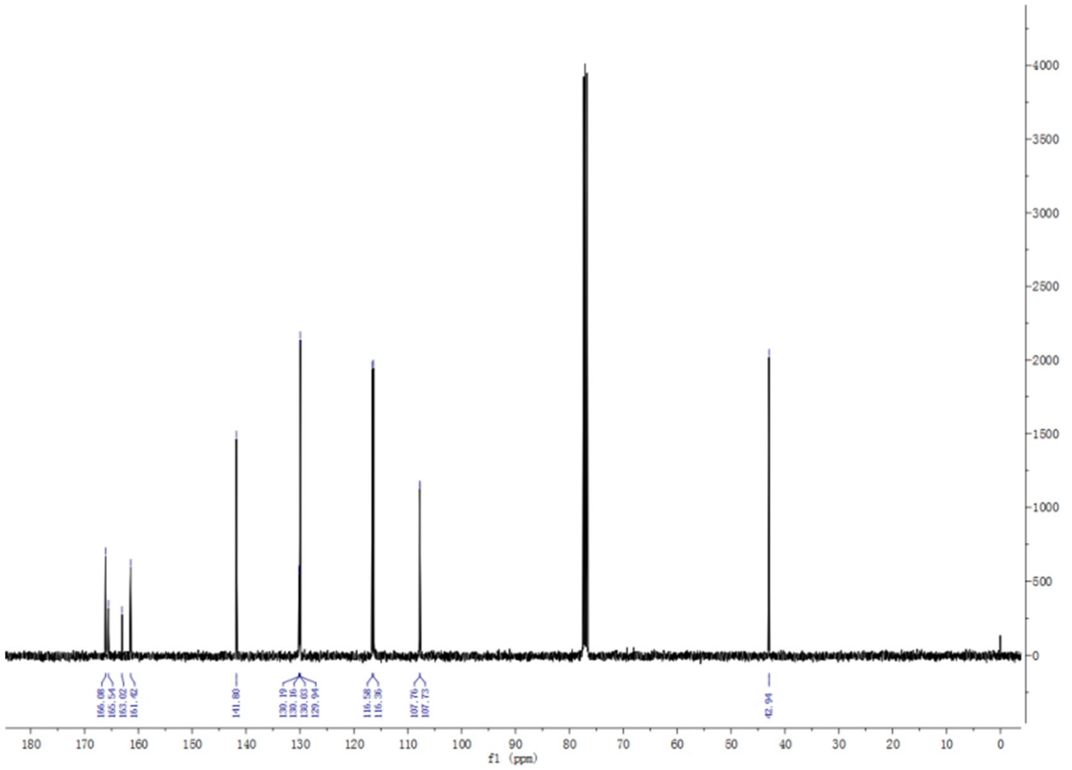

¹³C NMR of compound 5m

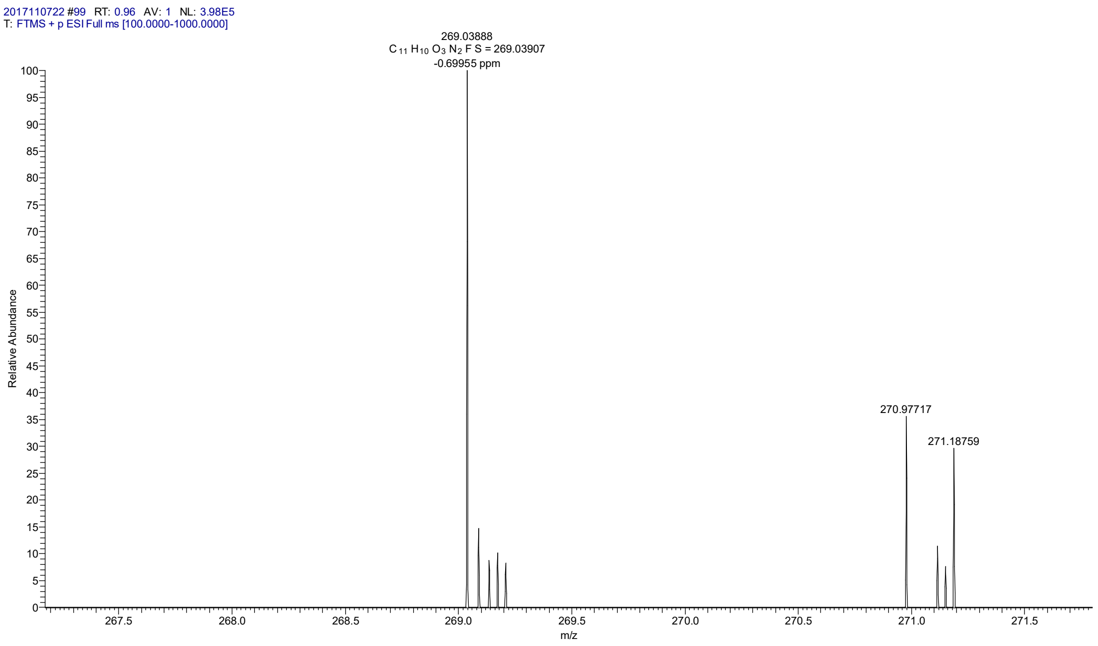


**HRMS (ESI) for compound 5m**

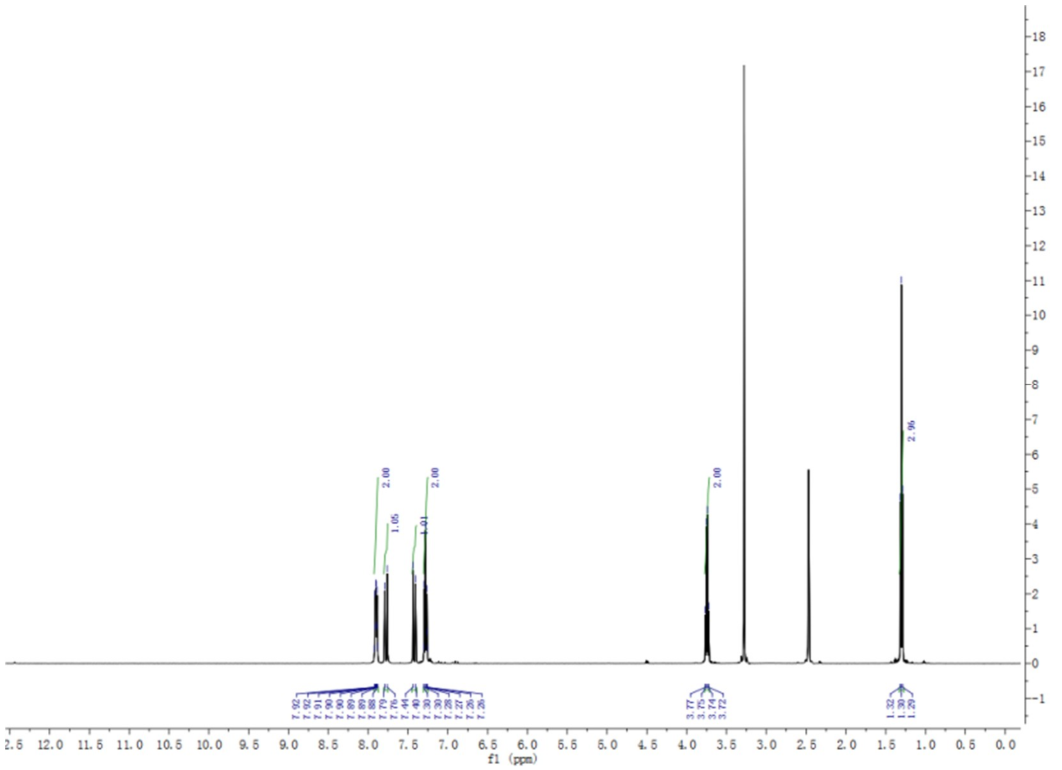


**¹H NMR for compound 5n**

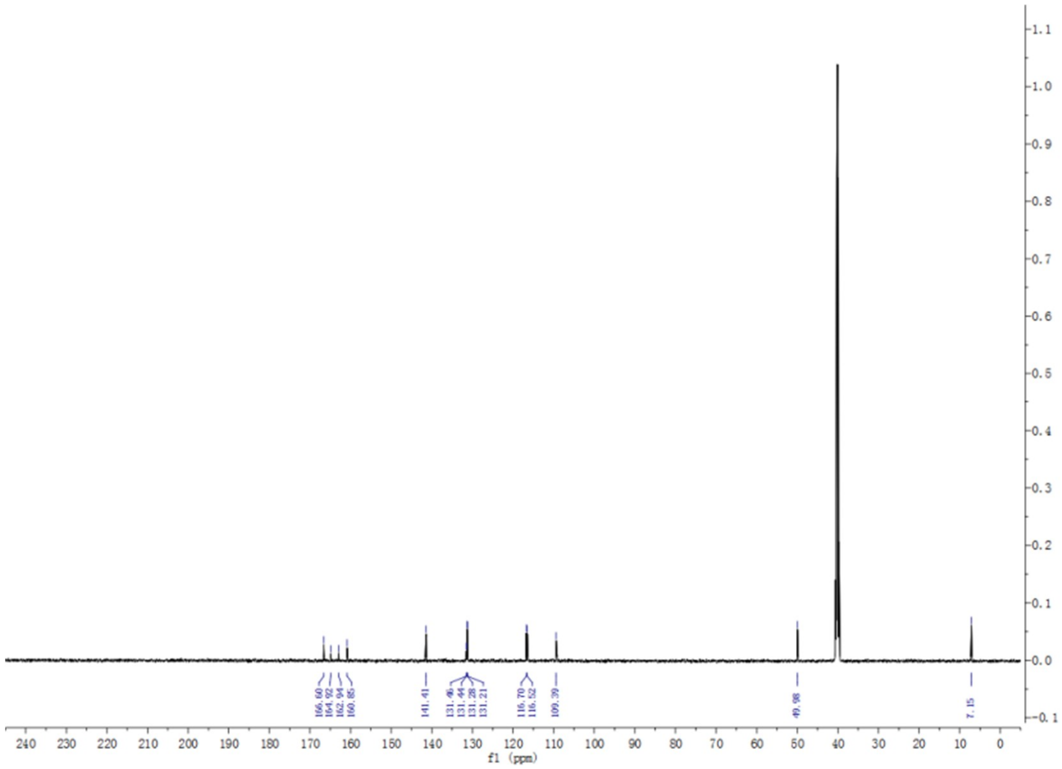



**¹³C NMR of compound 5n**

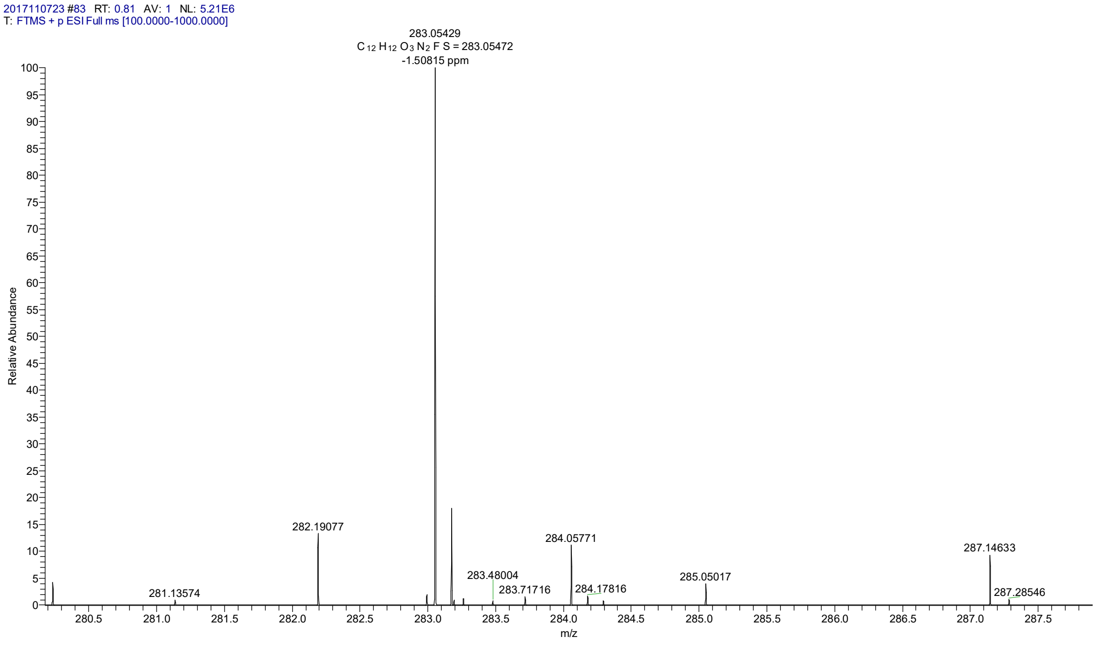



**HRMS (ESI) for compound 5n**

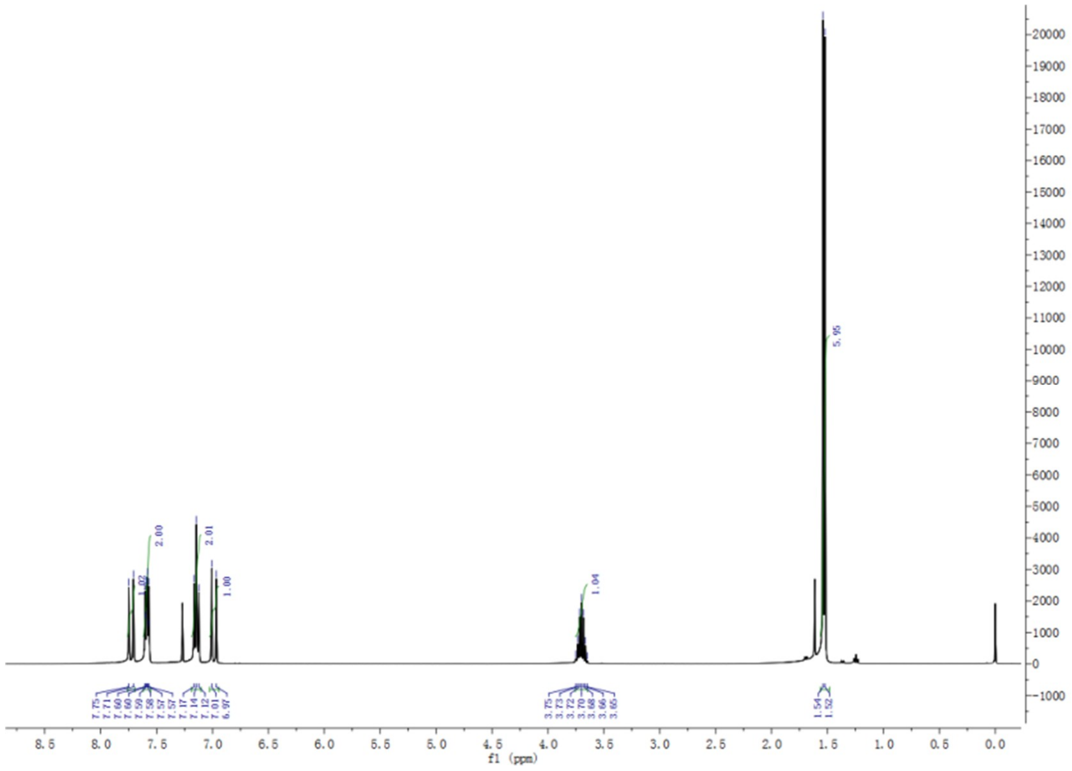

¹H NMR for compound 5o

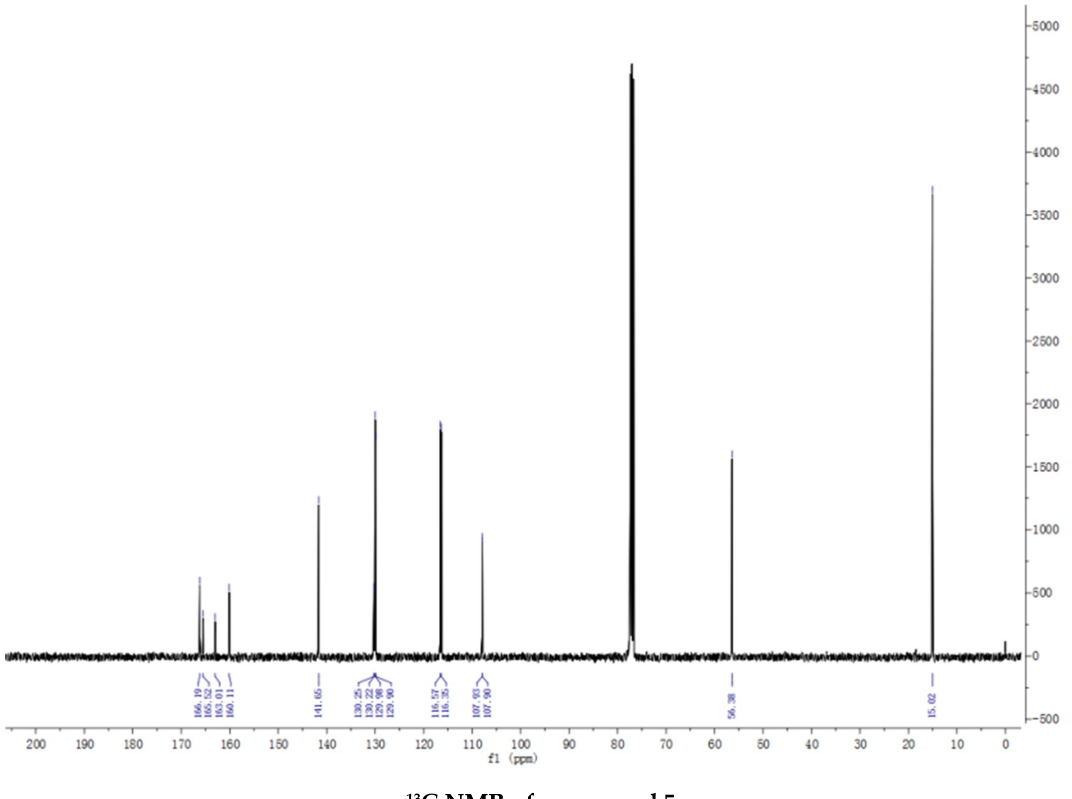

¹³C NMR of compound 5o

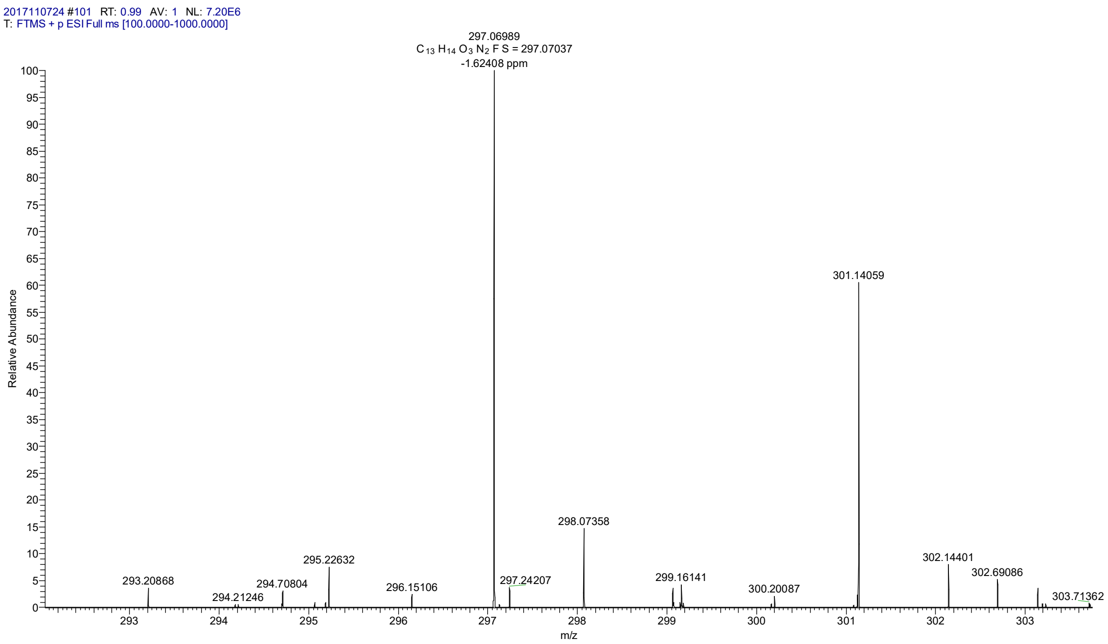


**HRMS (ESI) for compound 5o**

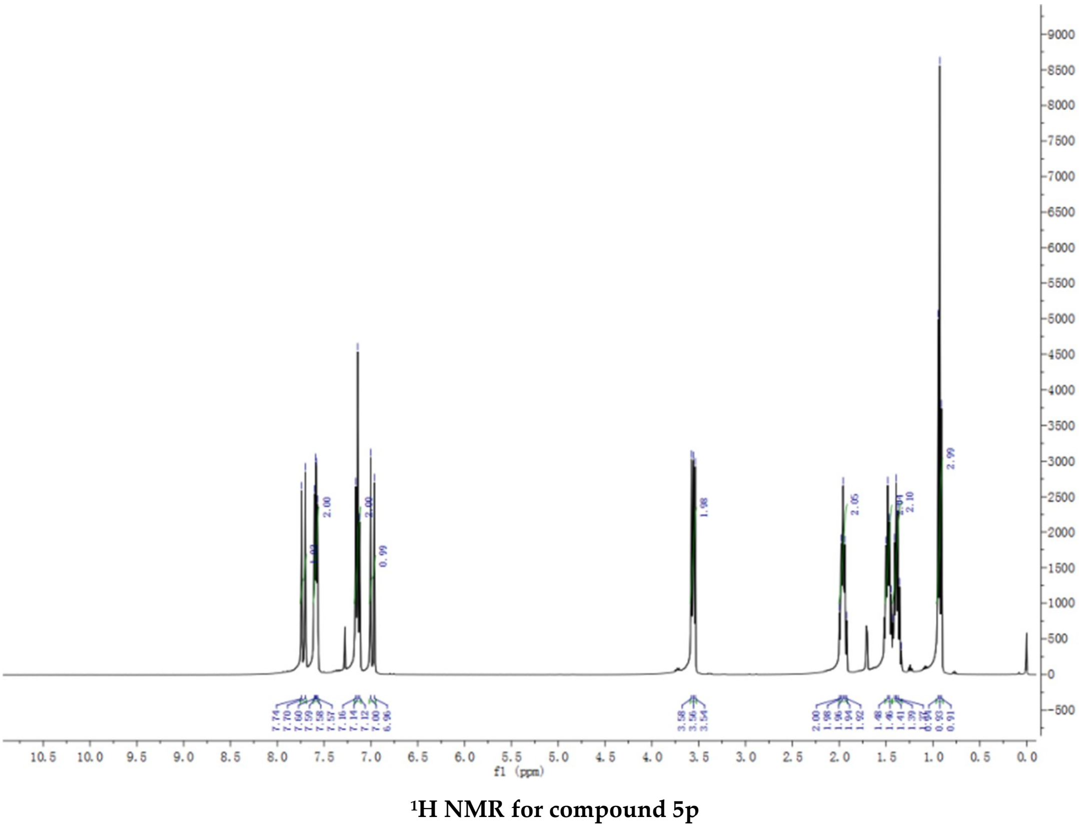


**$^{1}$H NMR for compound 5p**

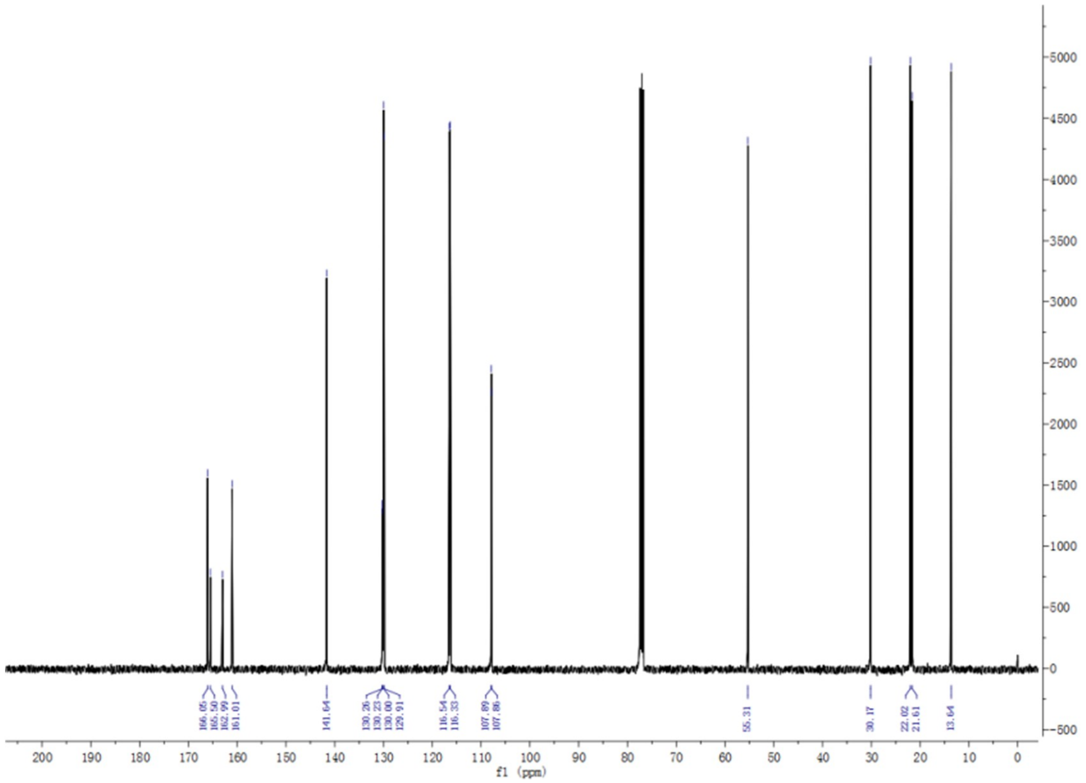

**$^{13}$C NMR of compound 5p**

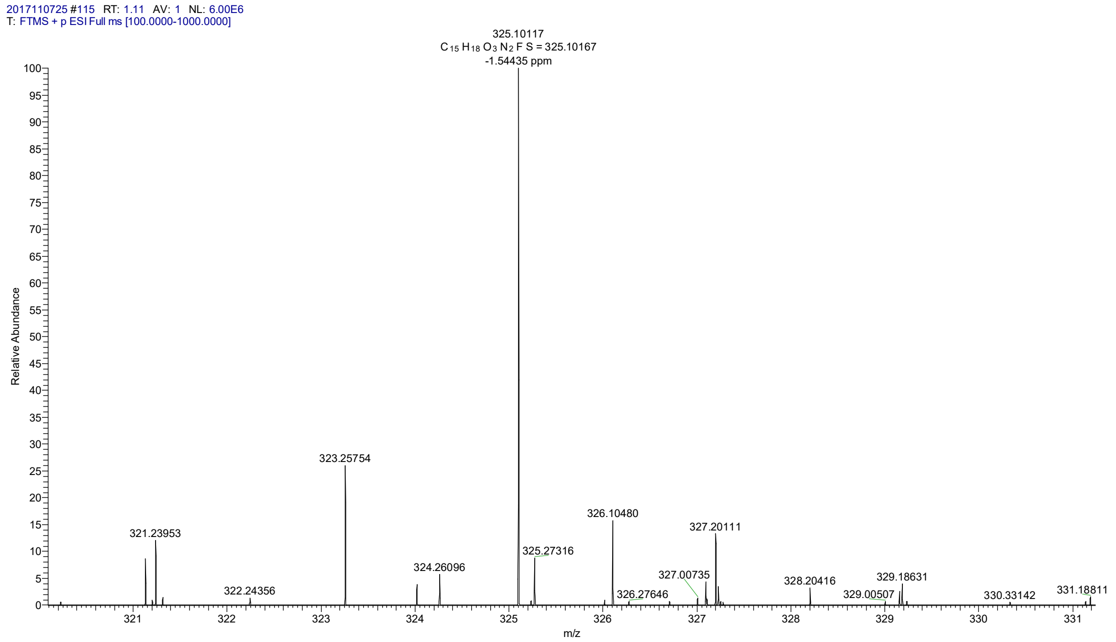

**HRMS (ESI) for compound 5p**

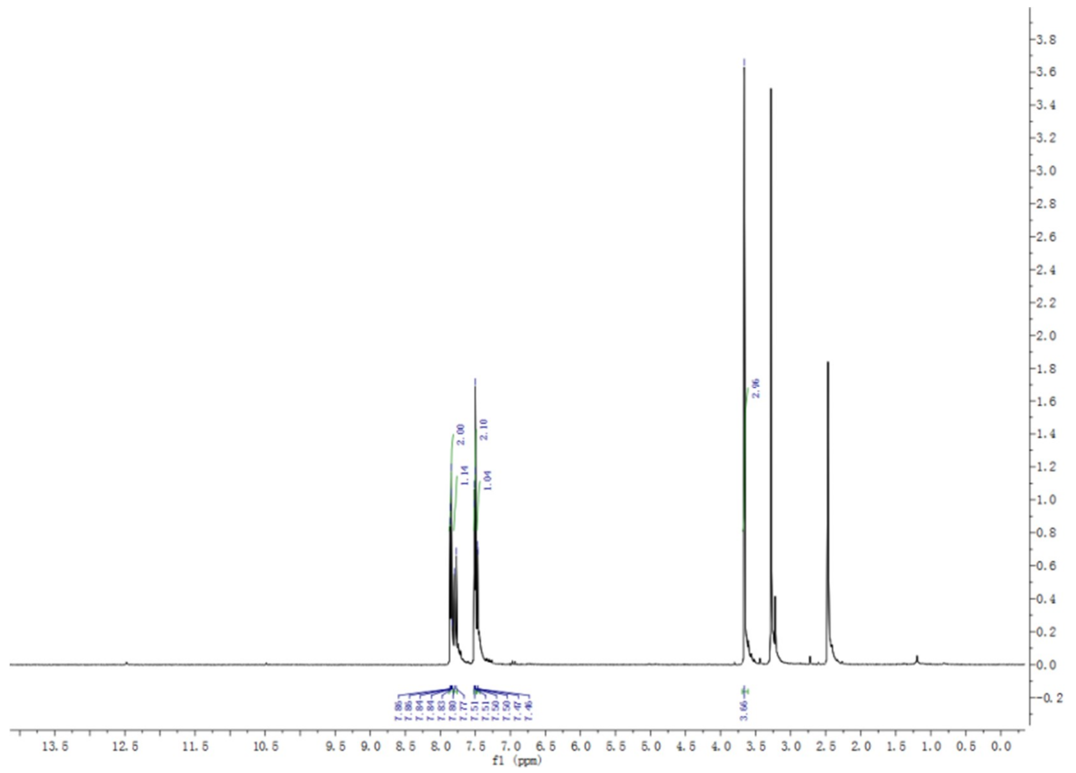

¹H NMR for compound 5q

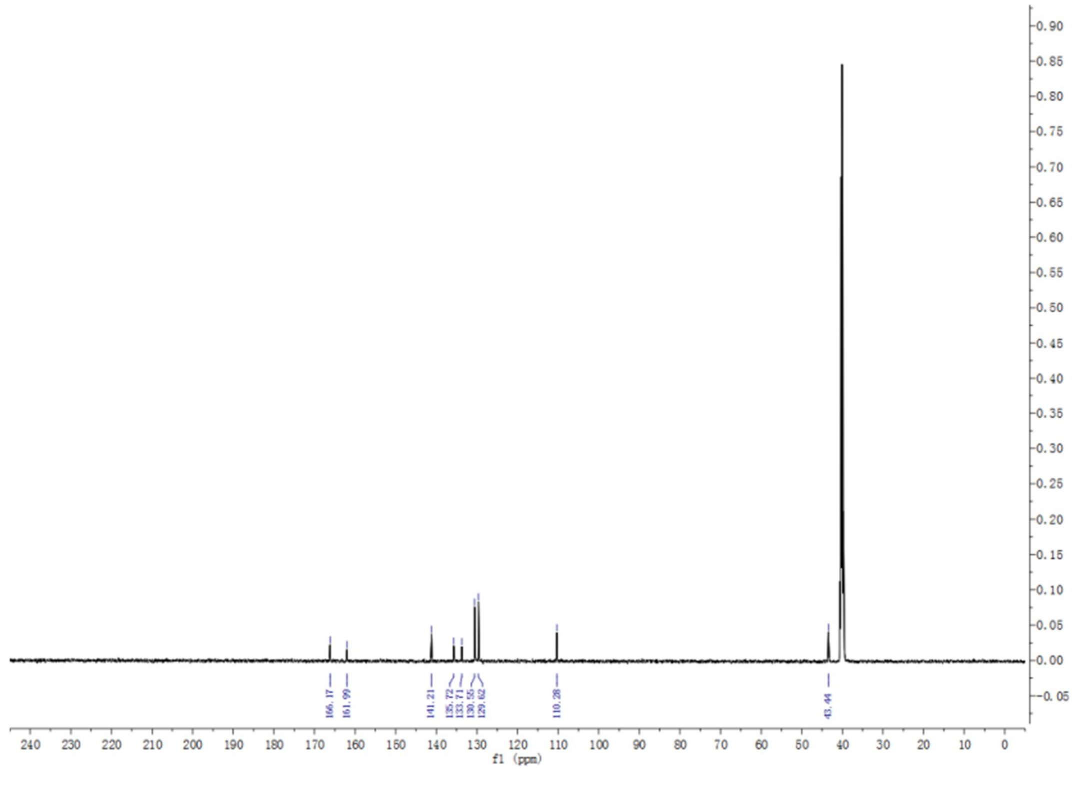

¹³C NMR of compound 5q

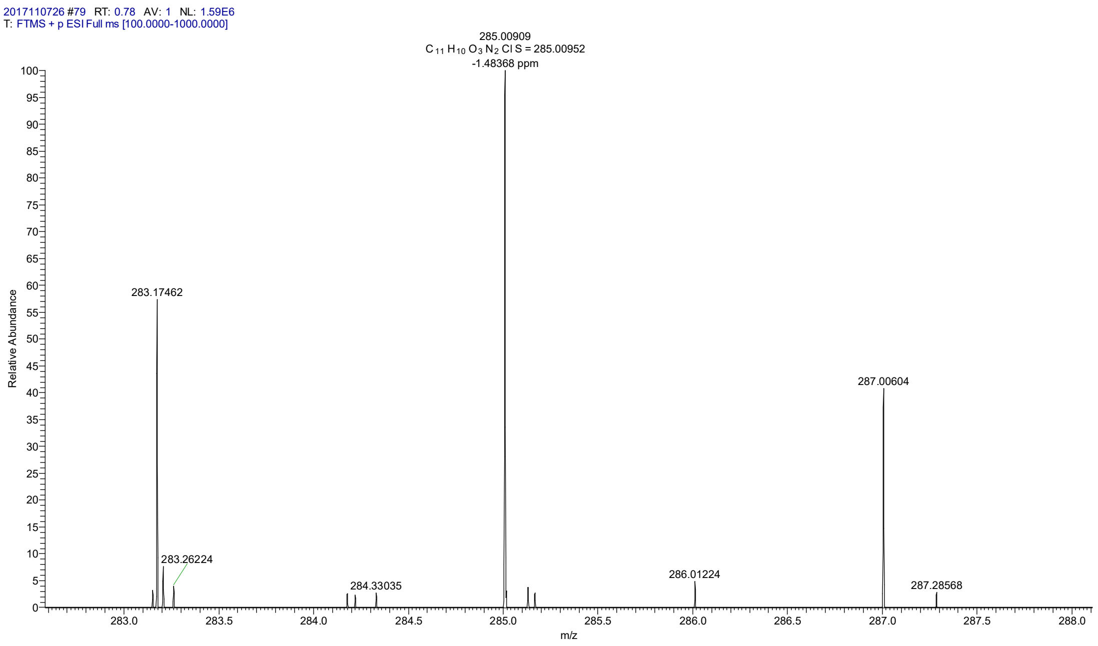


**HRMS (ESI) for compound 5q**

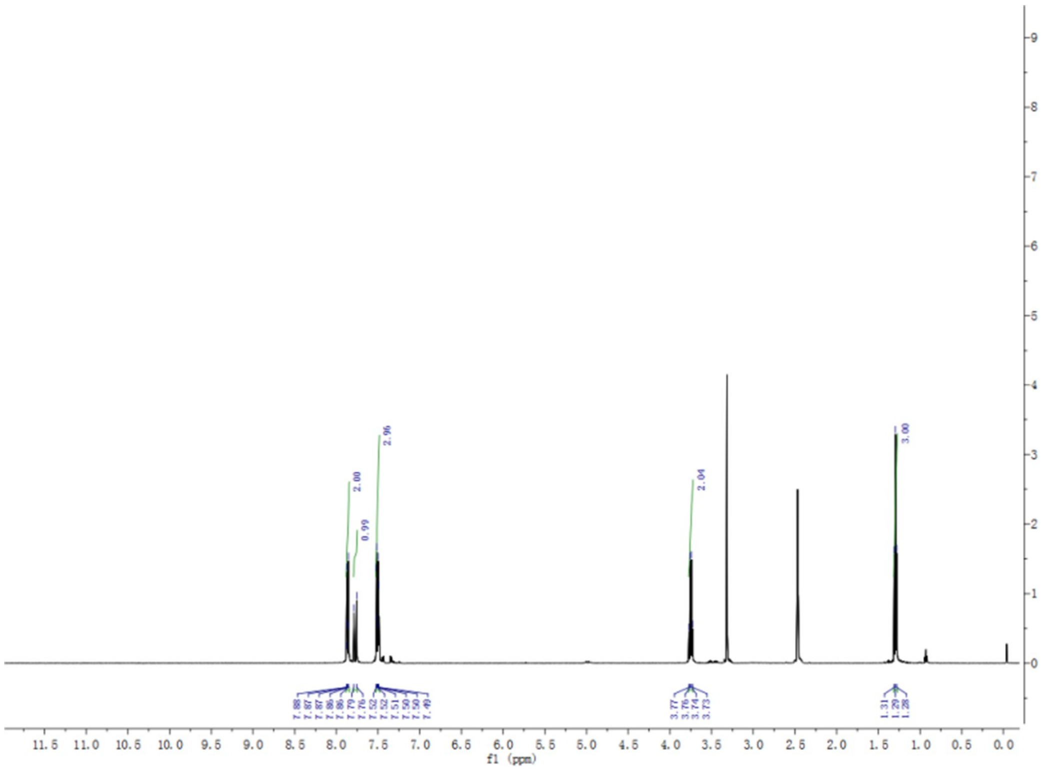


**$^{1}$H NMR of compound 5r**

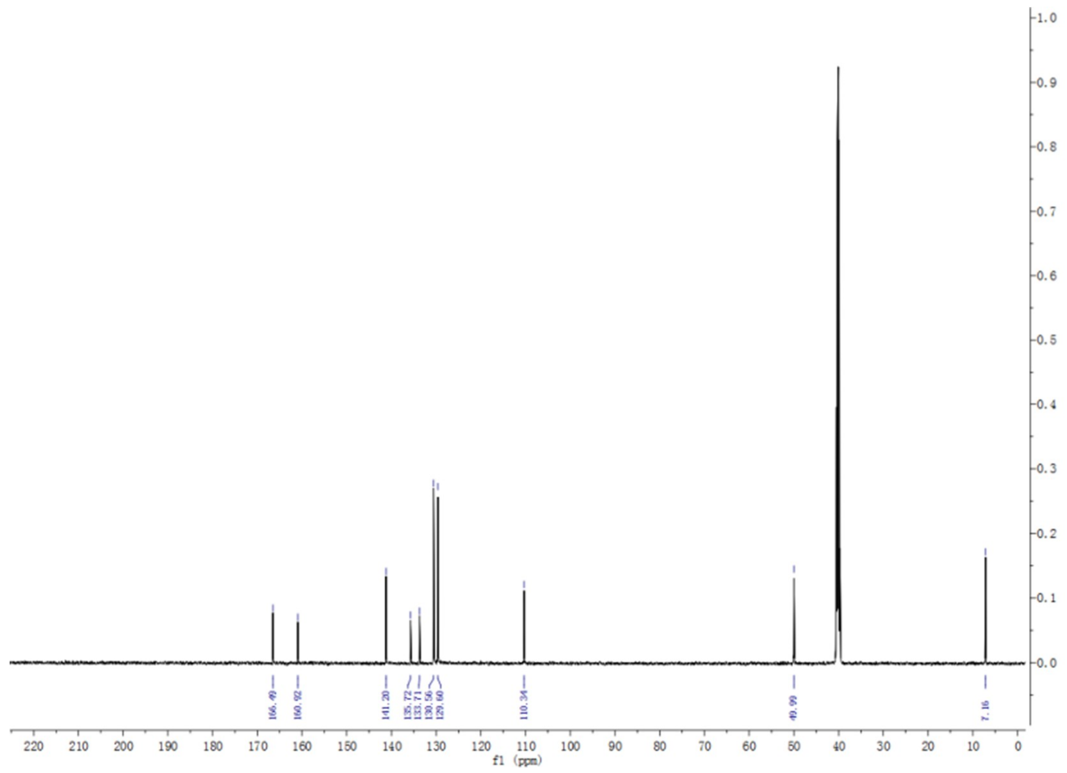


**$^{13}$C NMR of compound 5r**

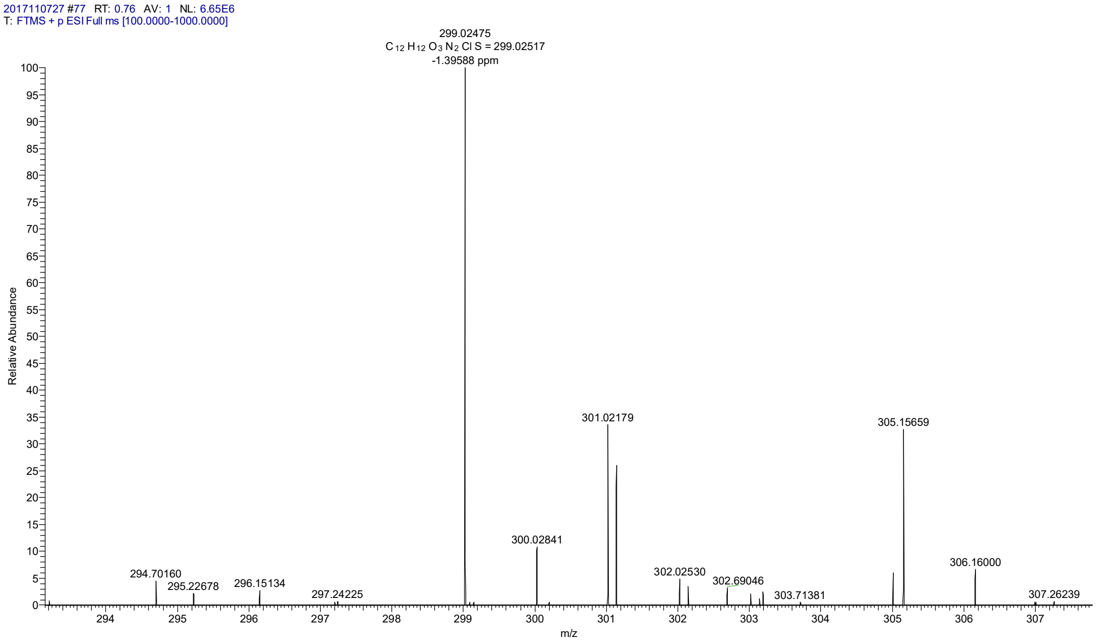


**HRMS (ESI) for compound 5r**

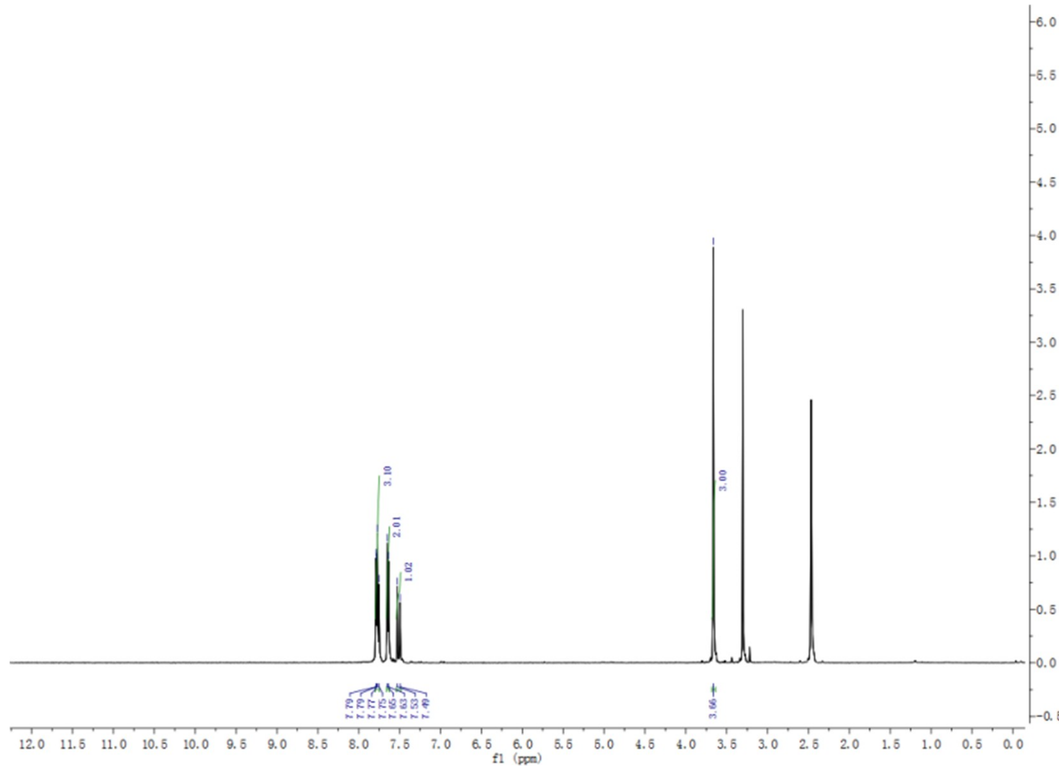

$^{1}$H NMR for compound 5s

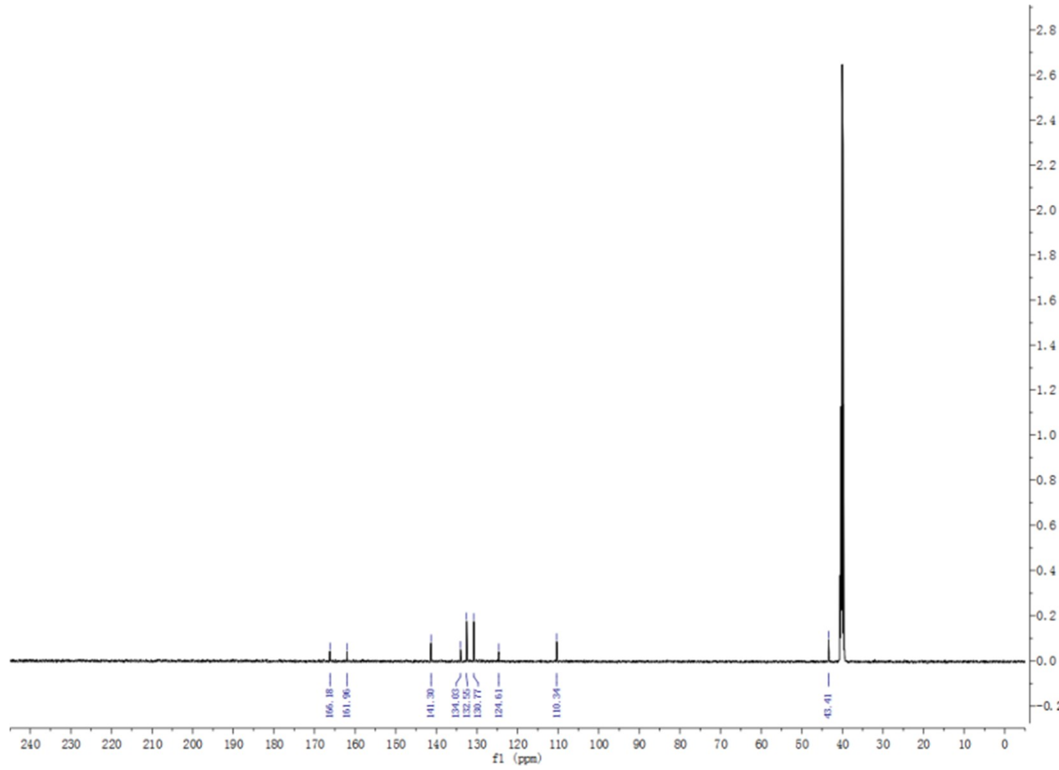

$^{13}$C NMR of compound 5s

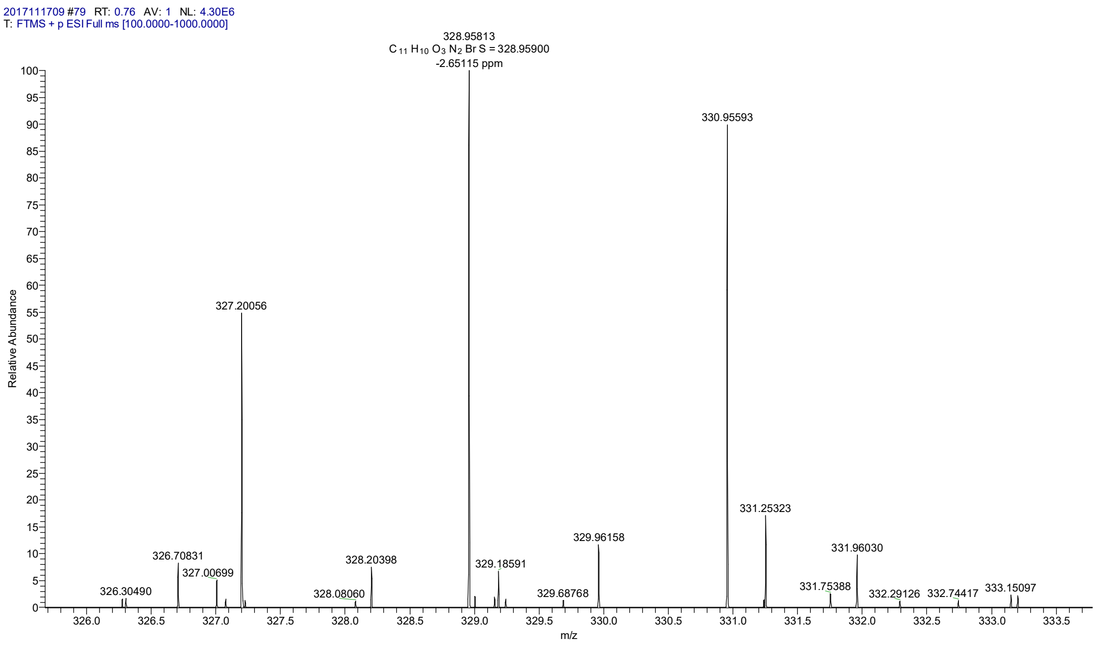


**HRMS (ESI) for compound 5s**

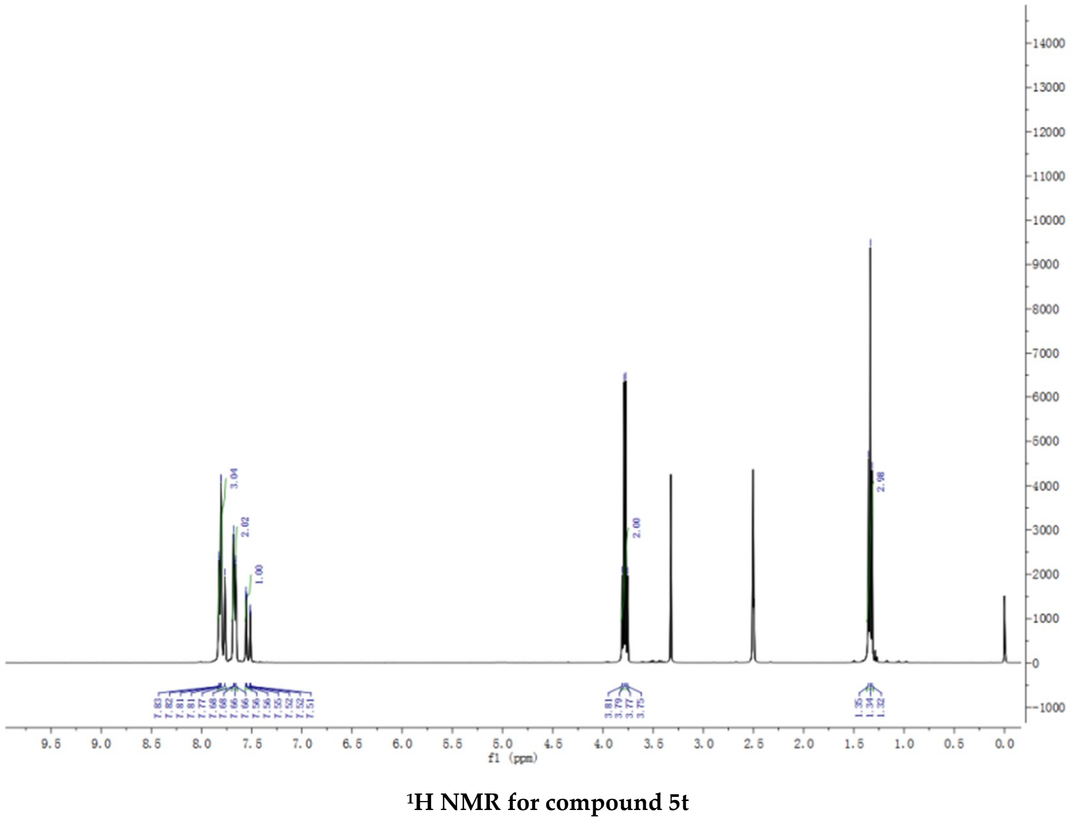


**$^{1}$H NMR for compound 5t**

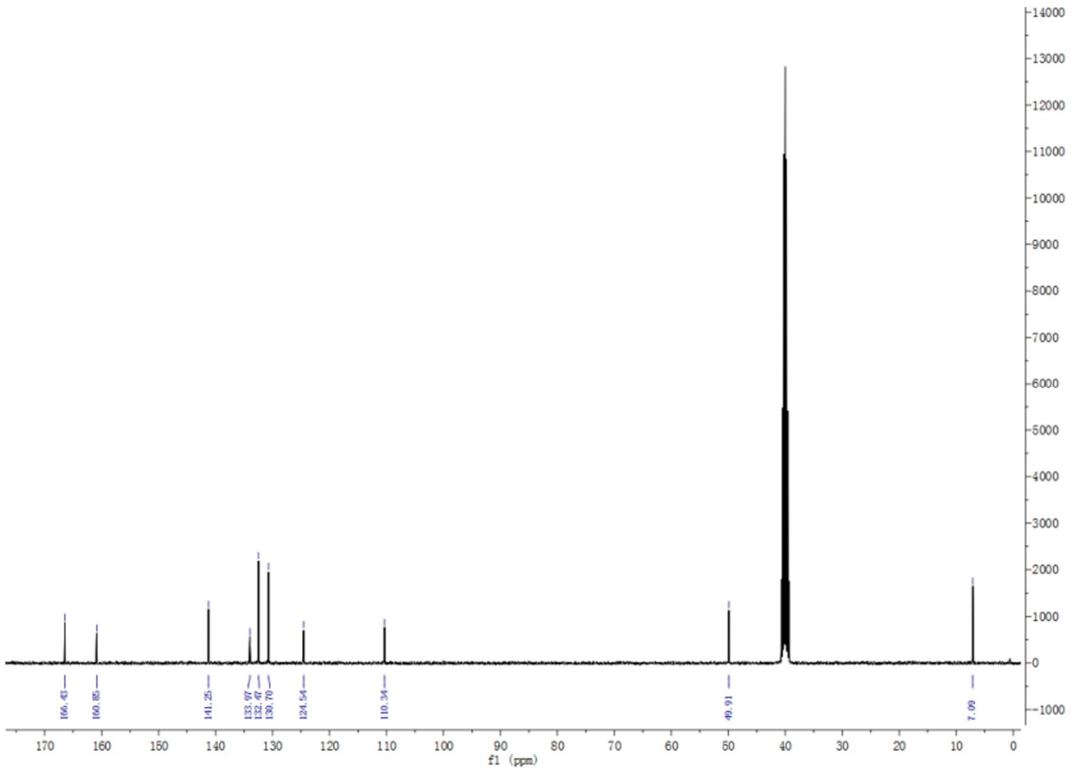


**¹³C NMR of compound 5t**

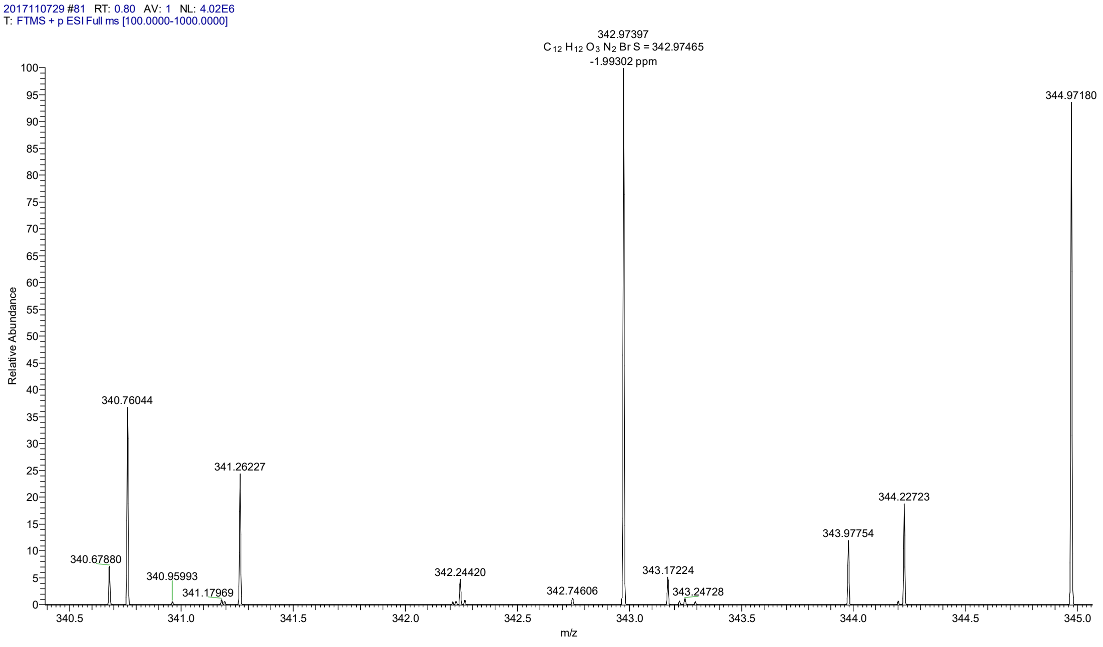


**HRMS (ESI) for compound 5t**

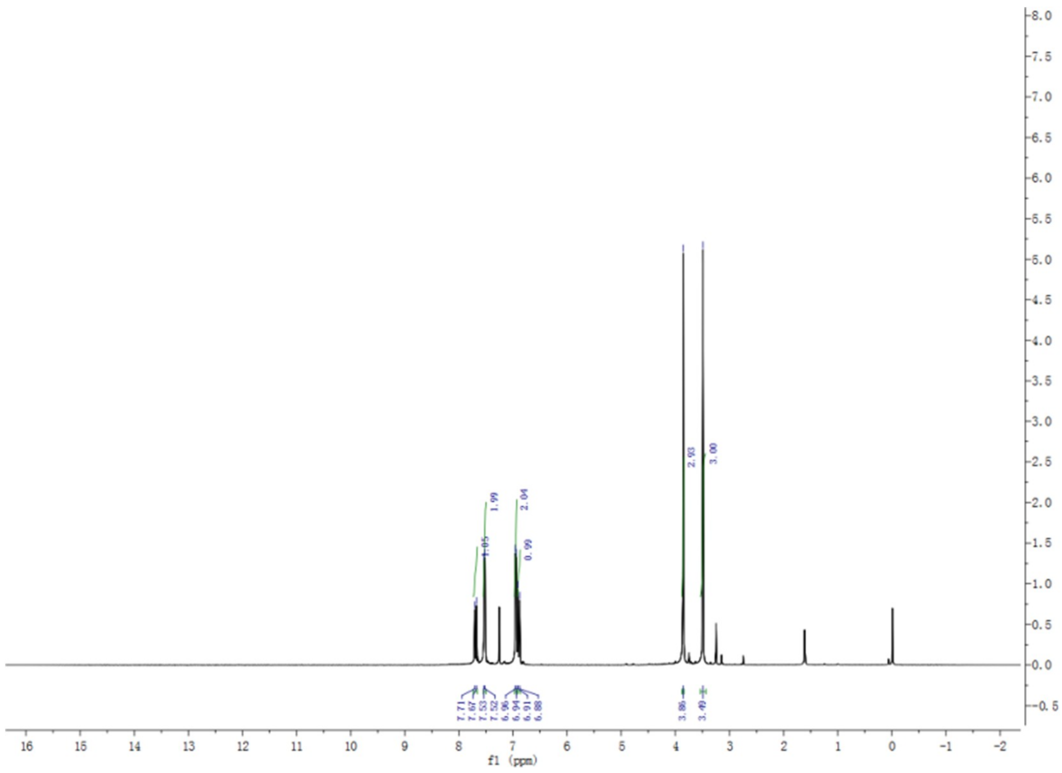

**¹H NMR for compound 5u**

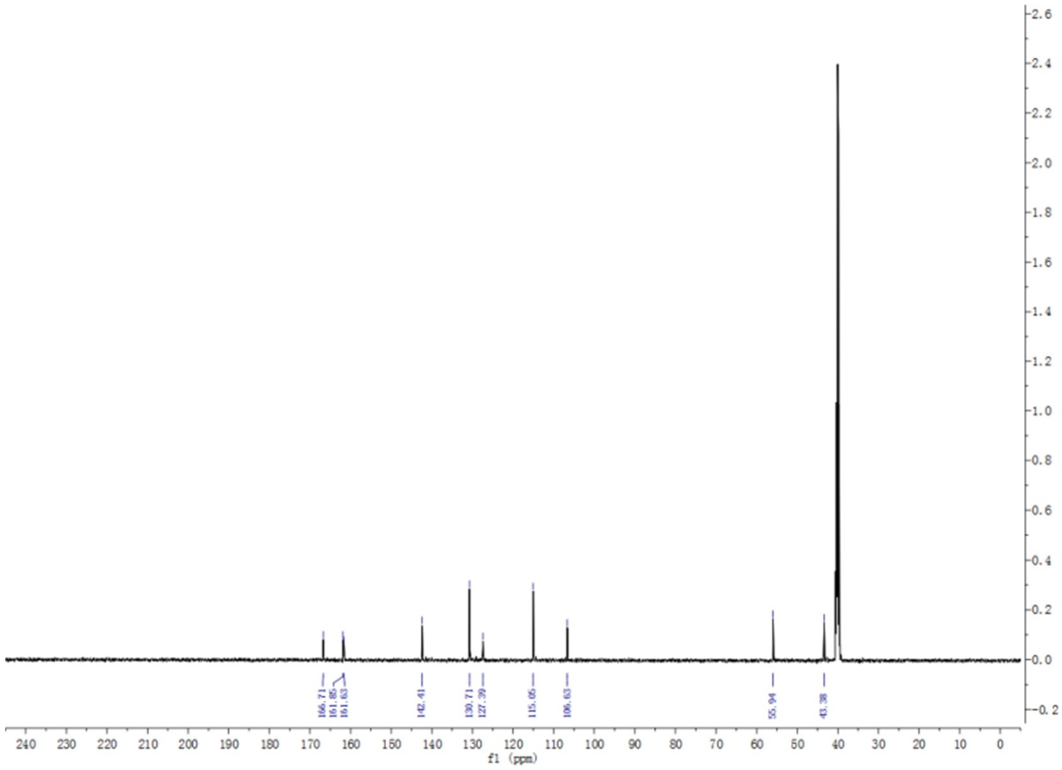

**¹³C NMR of compound 5u**

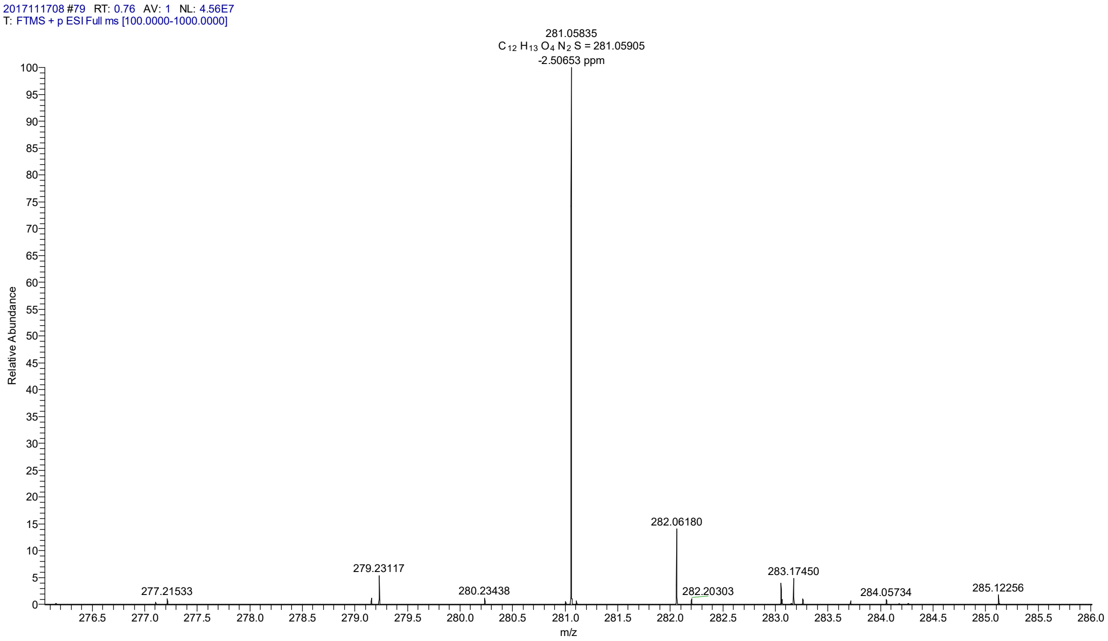


**HRMS (ESI) for compound 5u**

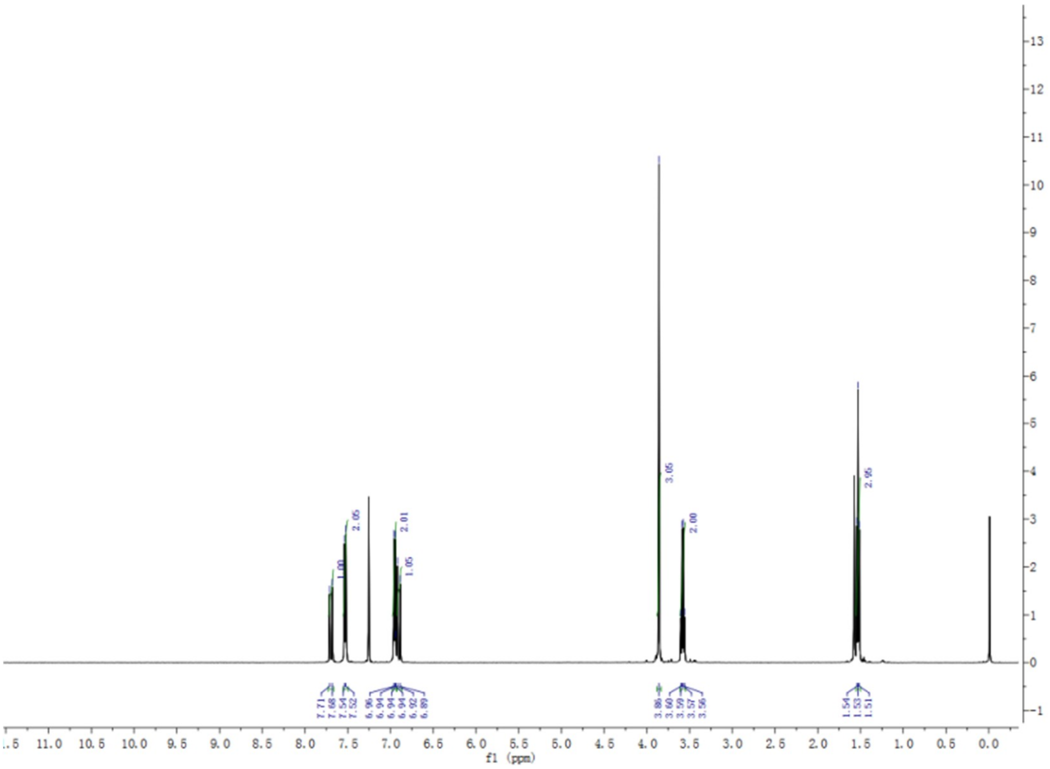


**¹H NMR for compound 5v**

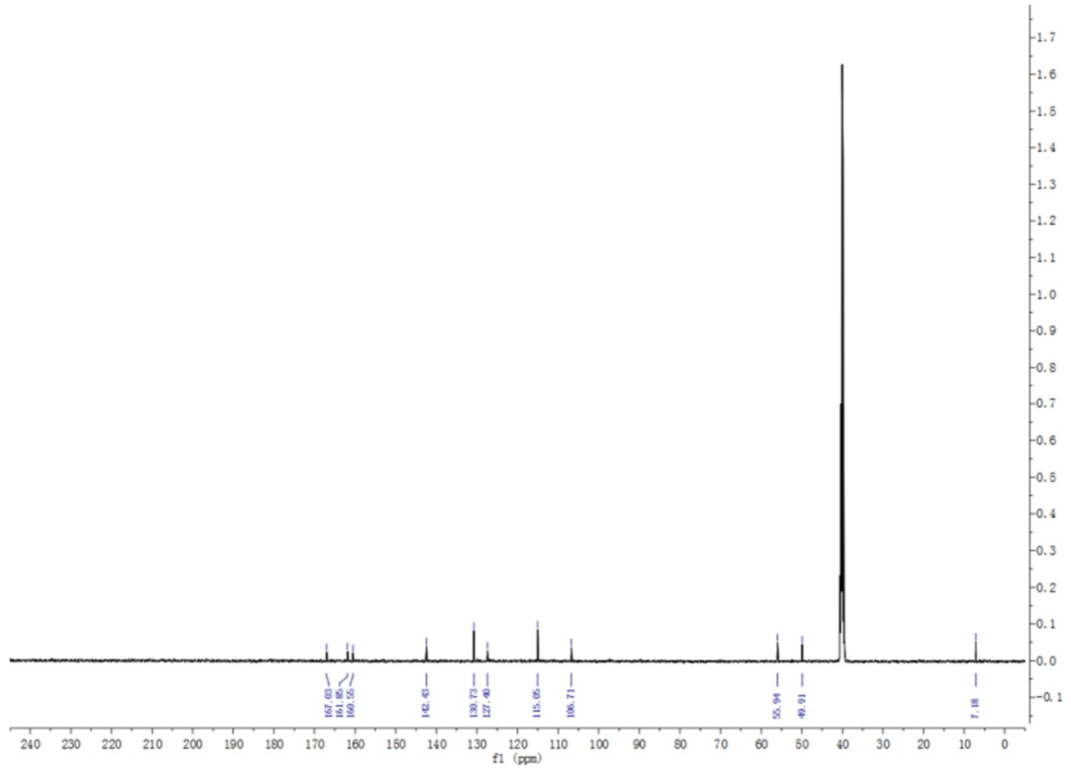


**$^{13}$C NMR of compound 5v**

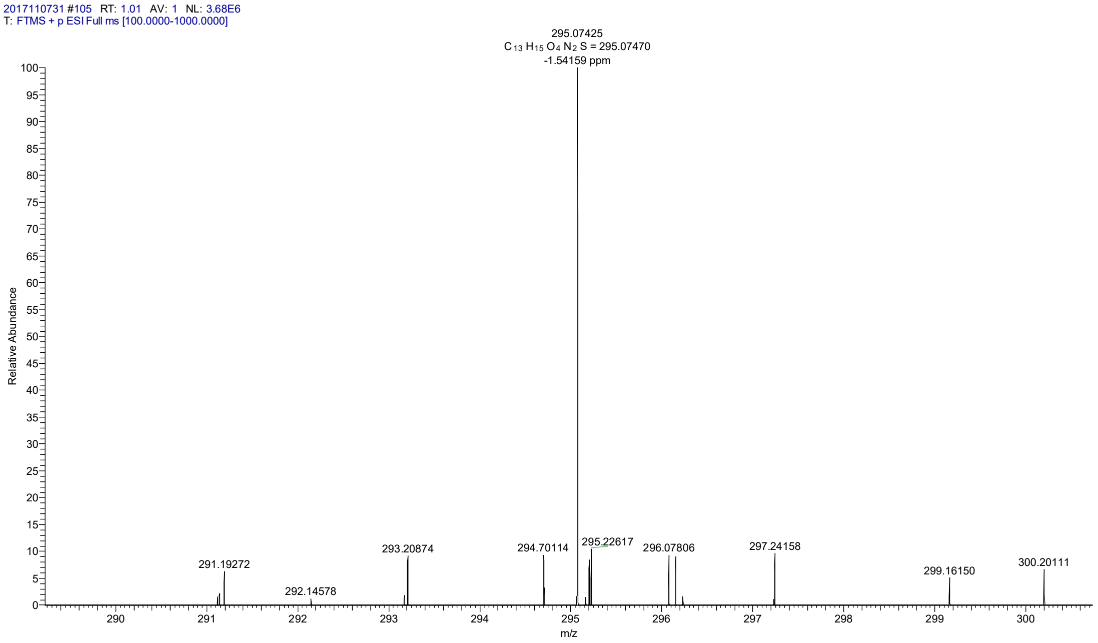


**HRMS (ESI) for compound 5v**

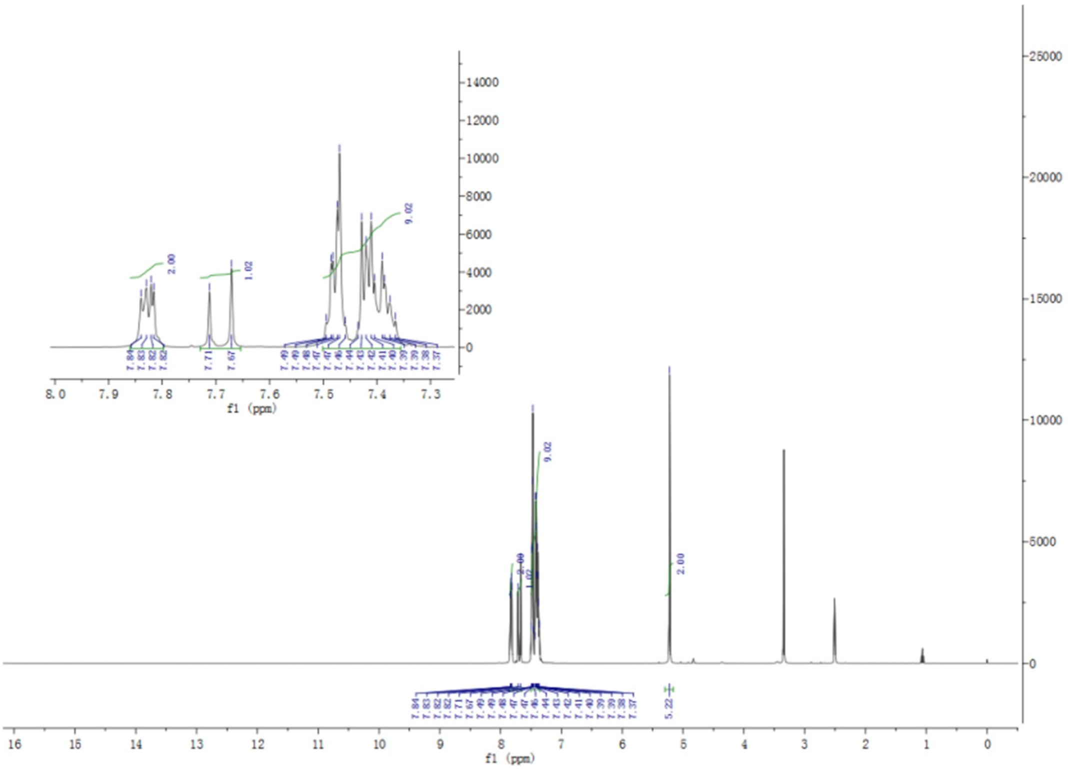


**¹H NMR for compound 5w**

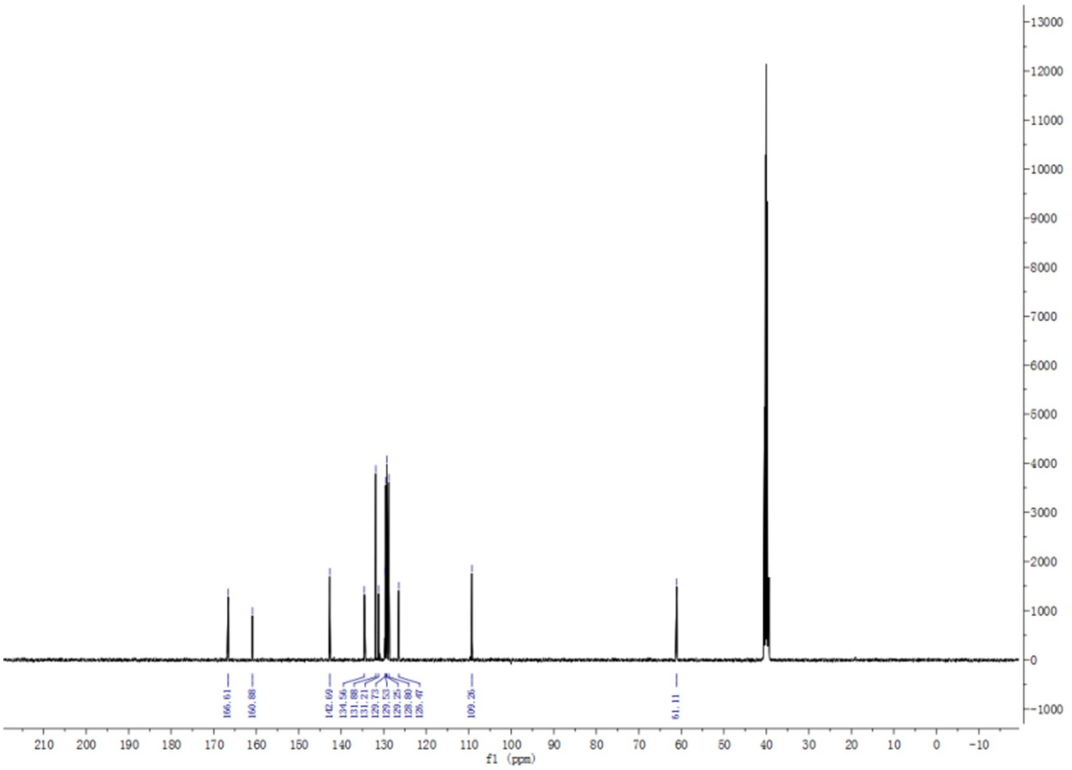


**¹³C NMR of compound 5w**

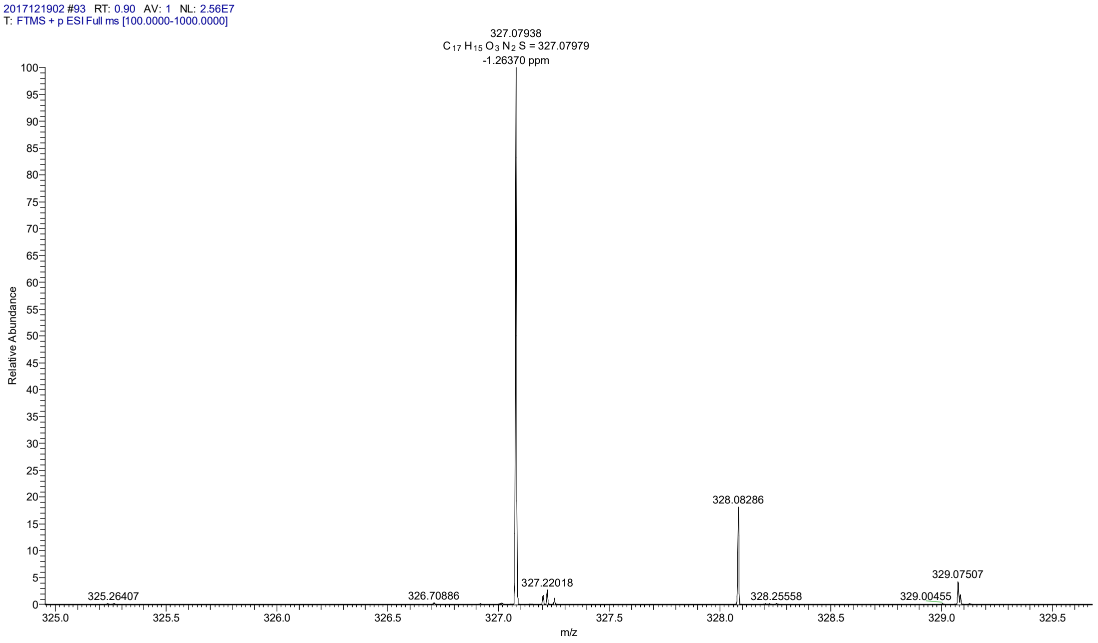


**HRMS (ESI) for compound 5w**

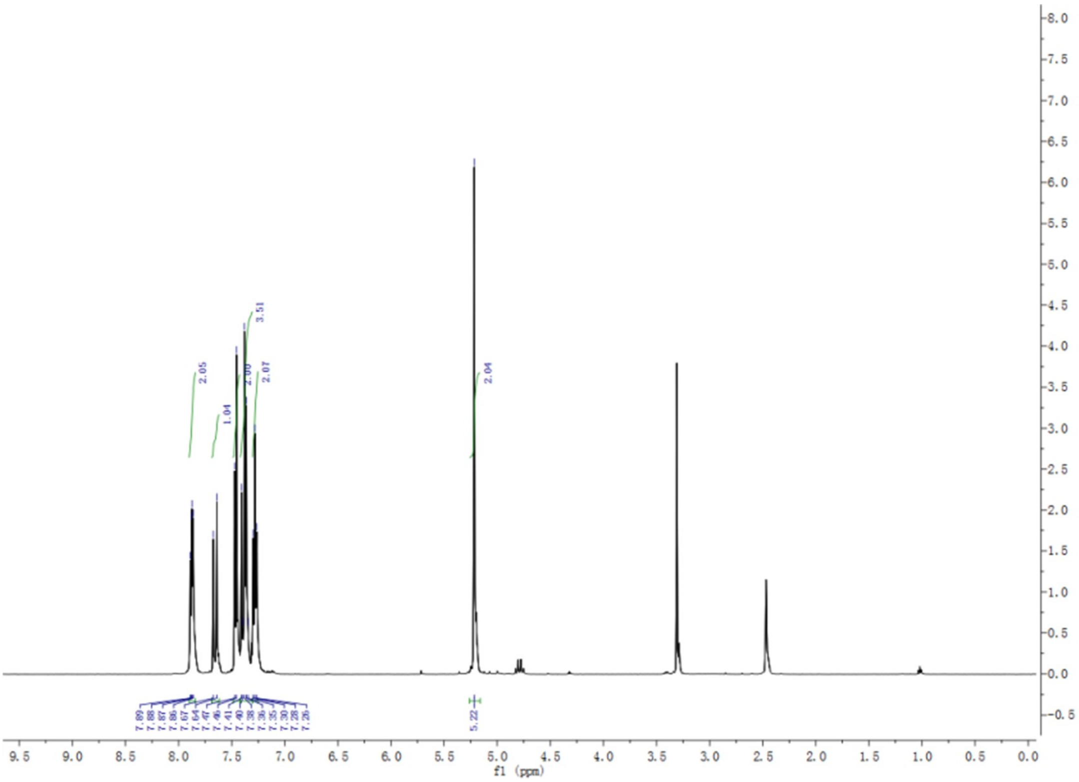


**$^1$H NMR for compound 5x**

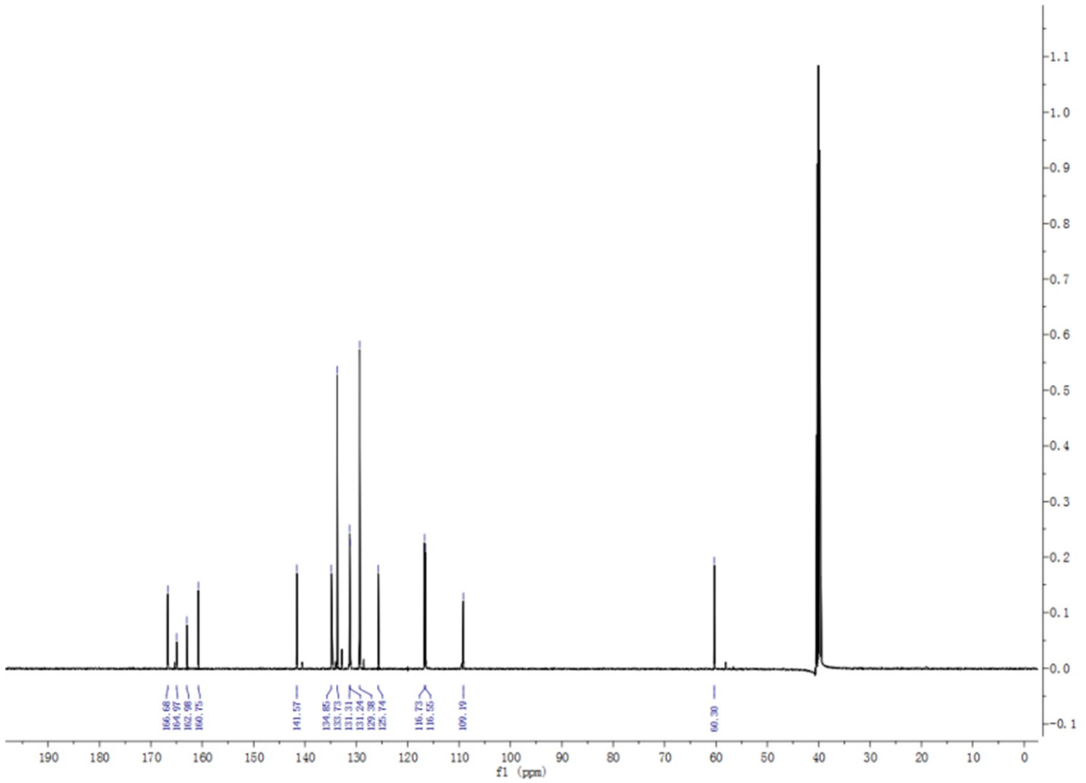


**$^{13}$C NMR of compound 5x**

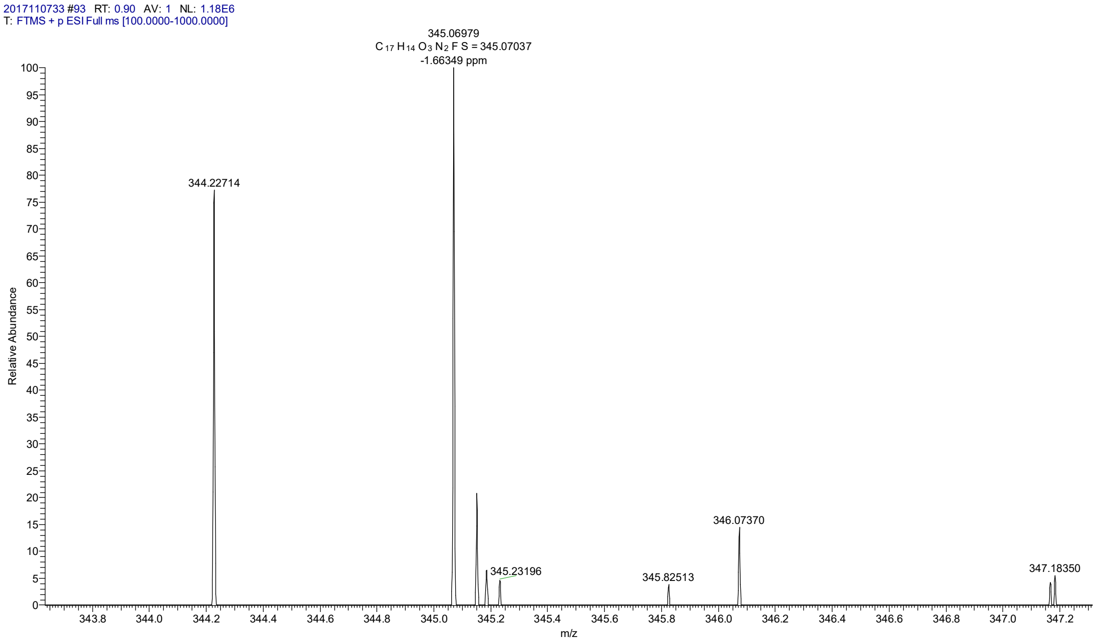


**HRMS (ESI) for compound 5x**

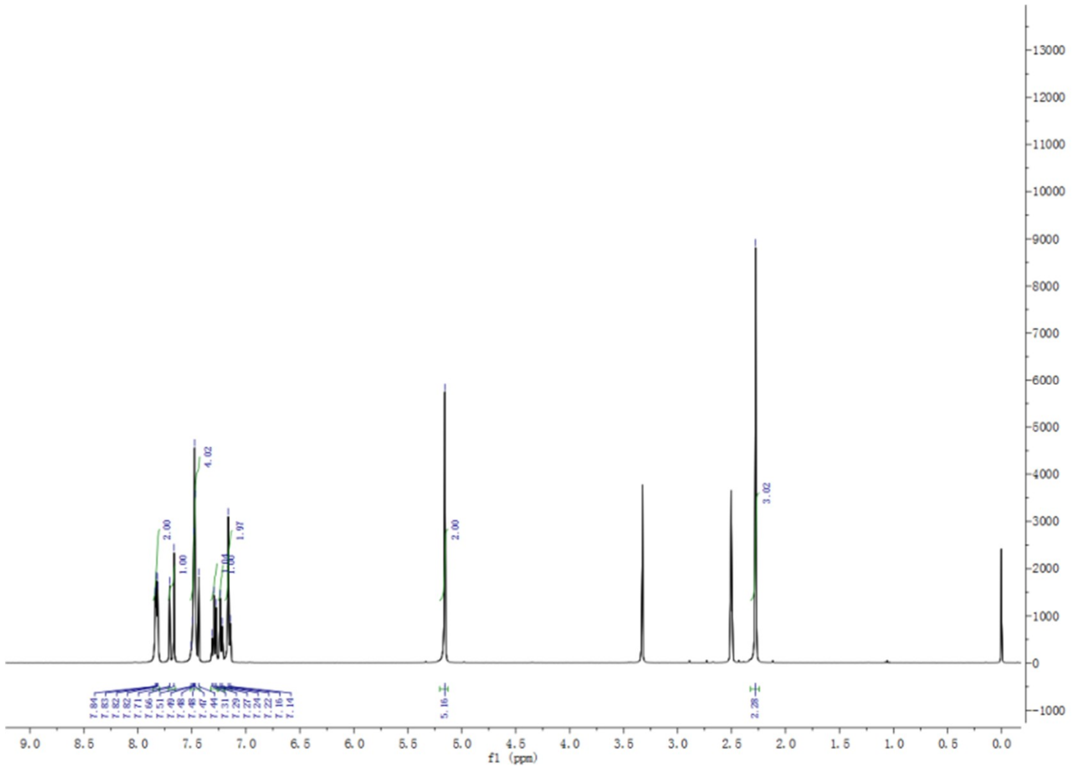

**¹H NMR for compound 5y**

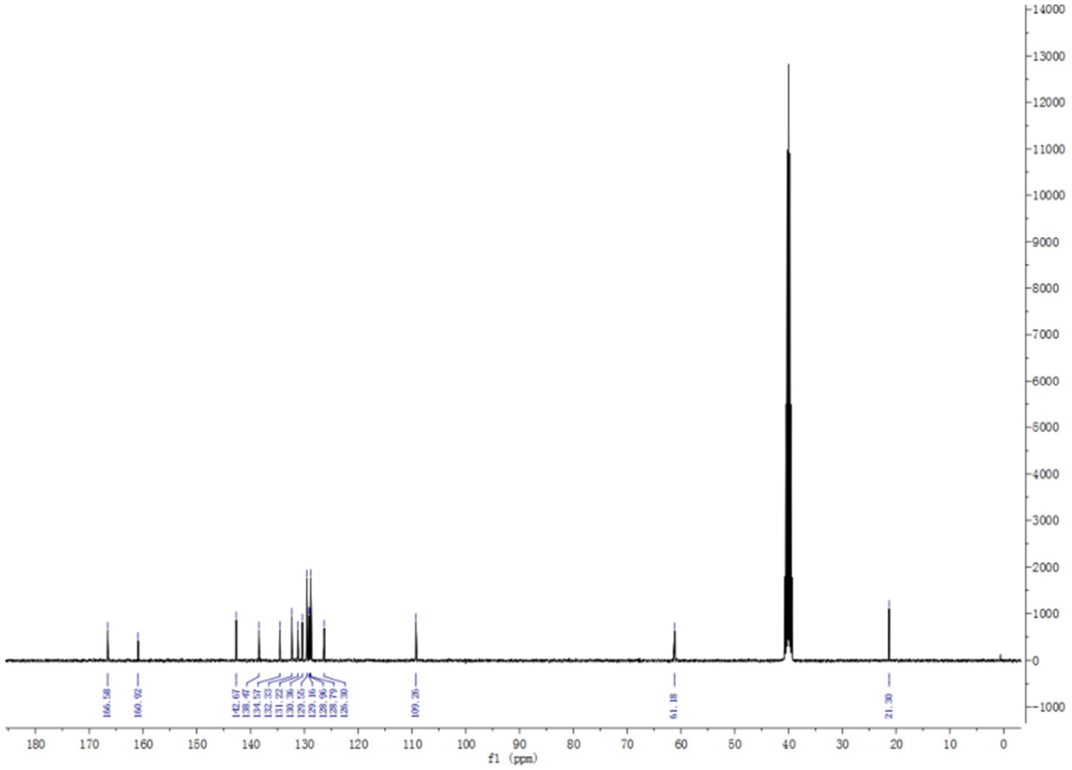

**¹³C NMR of compound 5y**

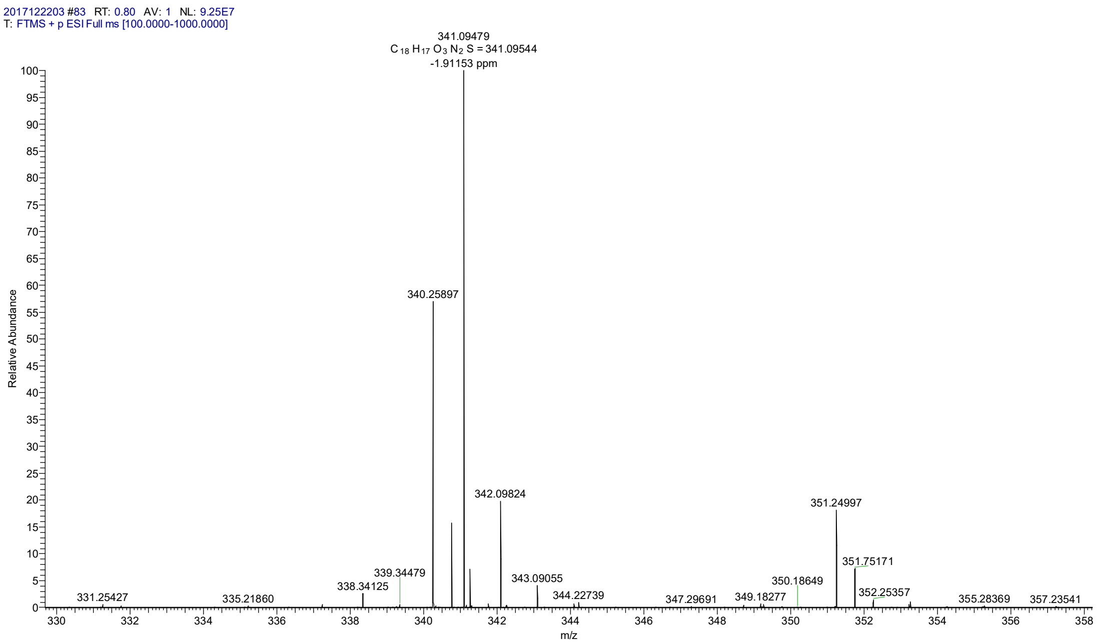


**HRMS (ESI) for compound 5y**

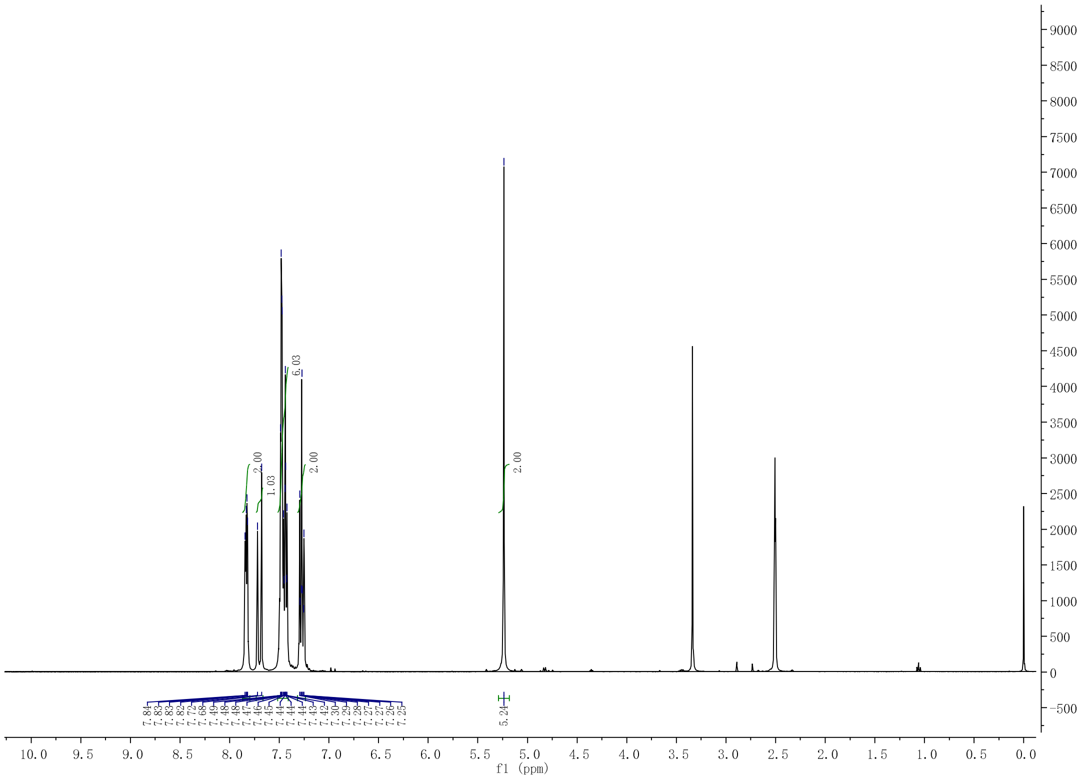


**¹H NMR for compound 5z**

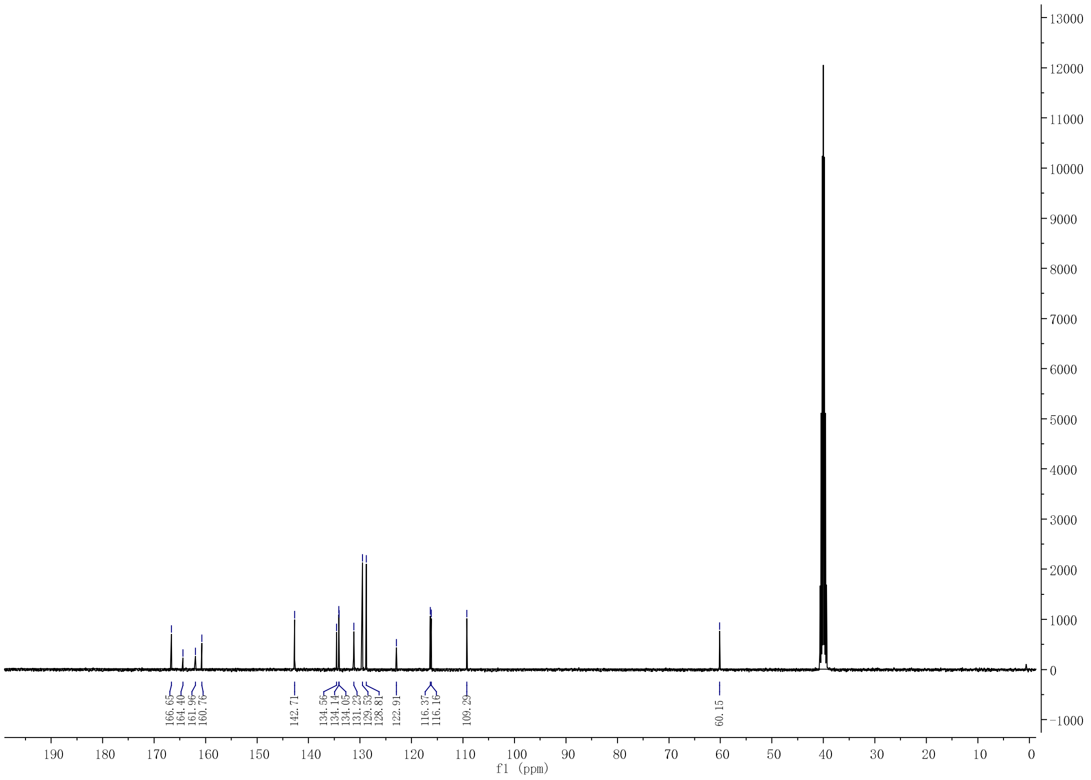


**$^{13}C$ NMR of compound 5z**

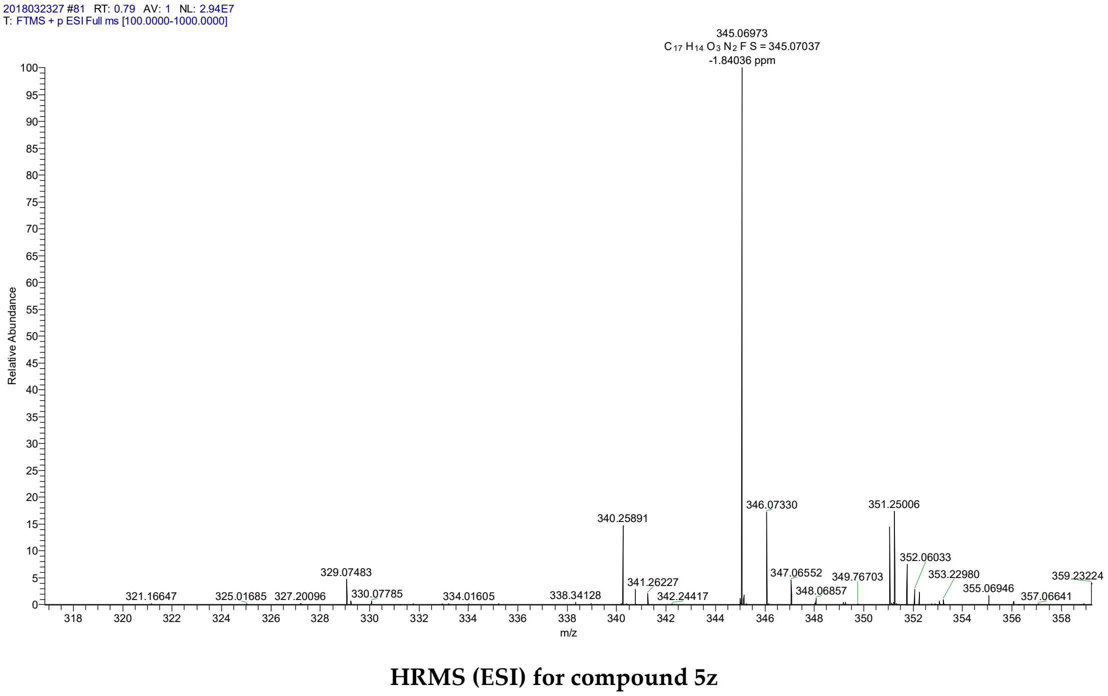


**HRMS (ESI) for compound 5z**

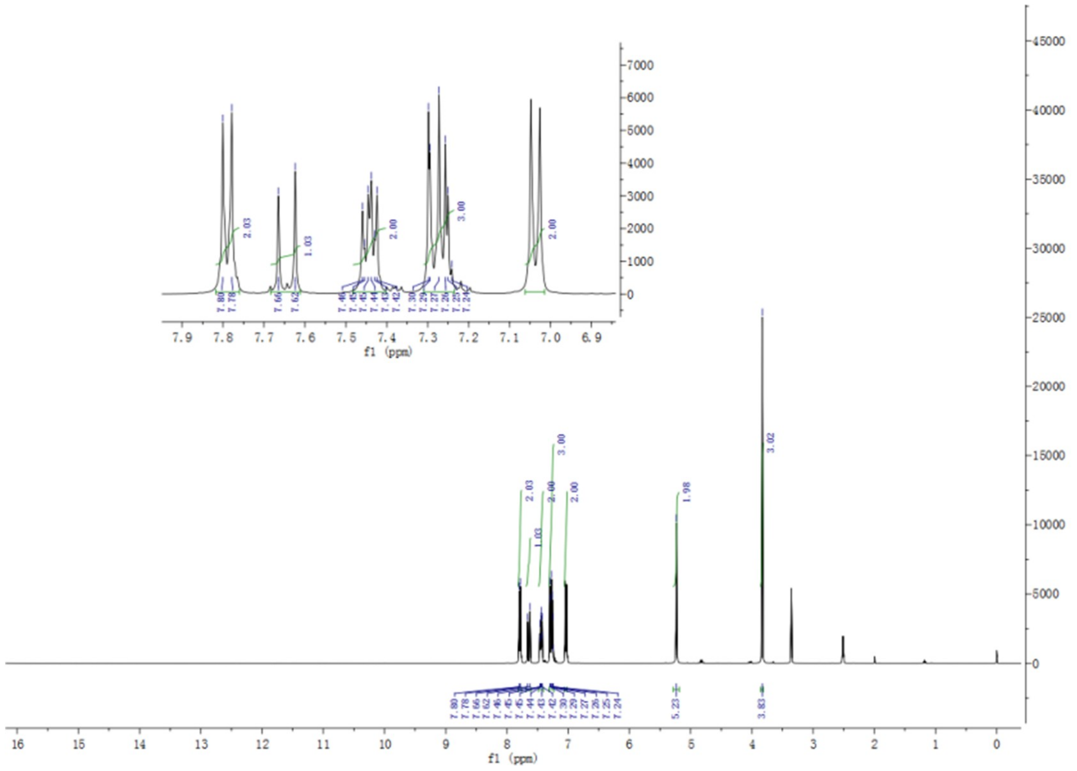

¹H NMR for compound 5aa

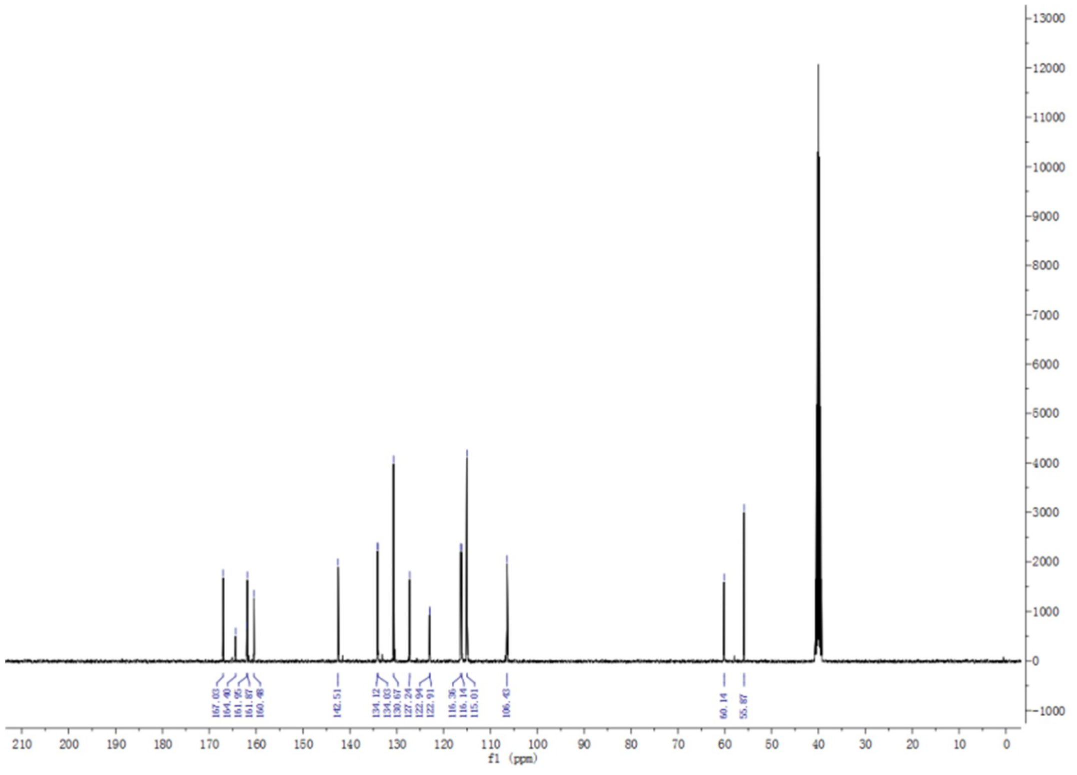

¹³C NMR of compound 5aa

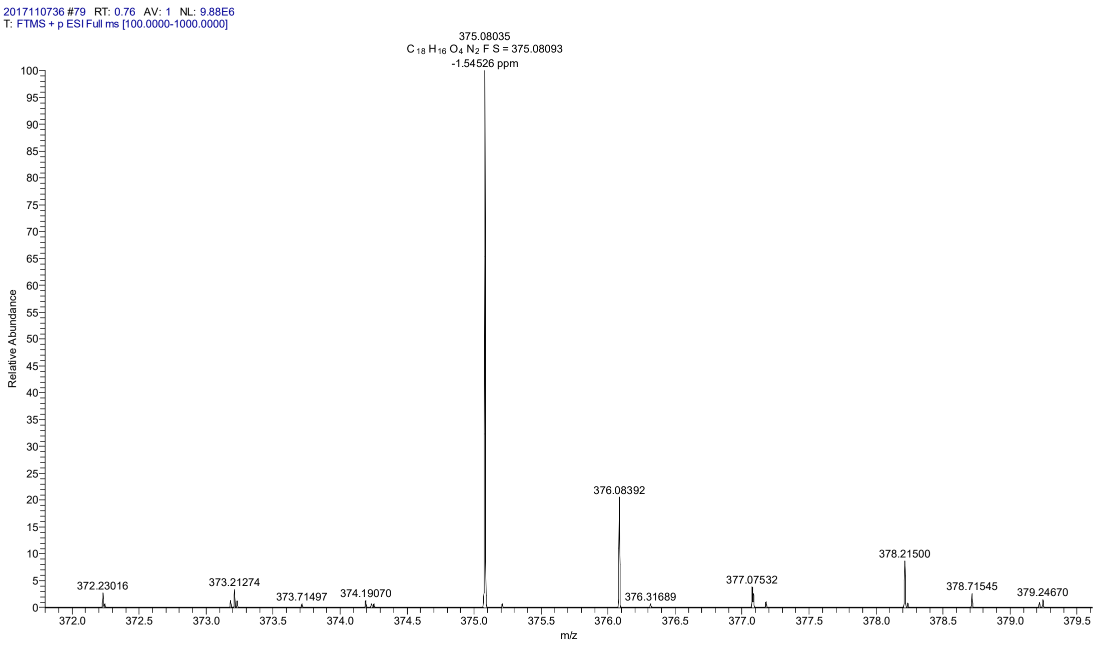


**HRMS (ESI) for compound 5aa**

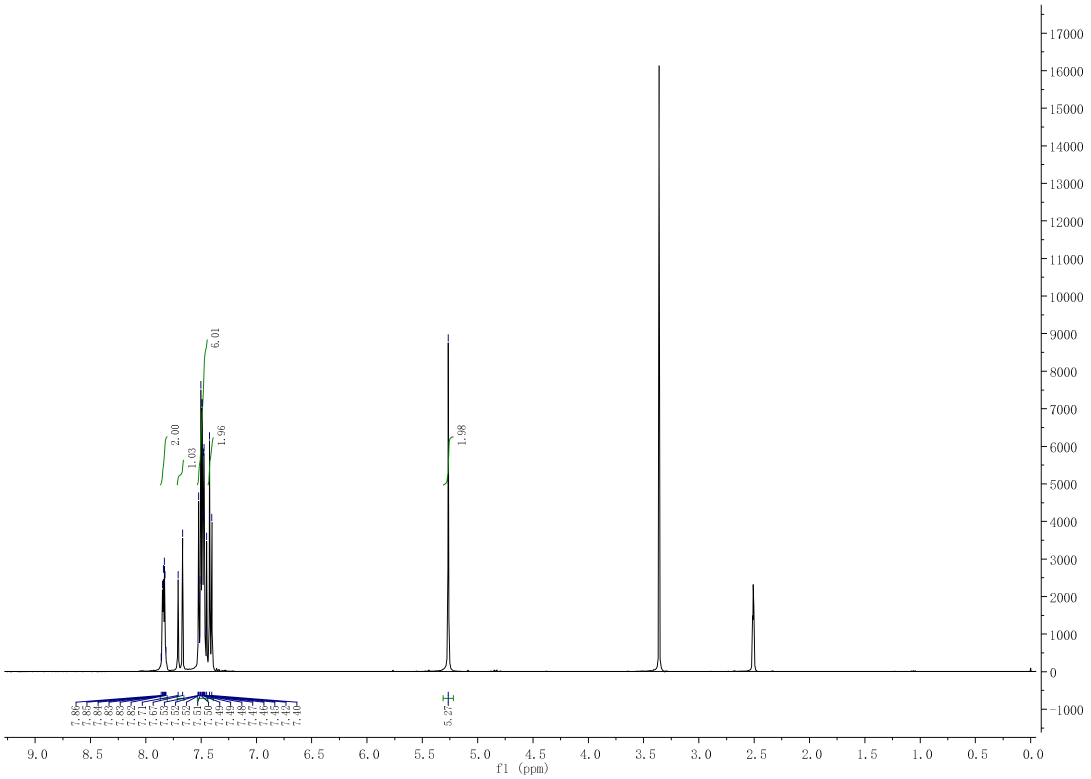


**$^{1}$H NMR for compound 5ab**

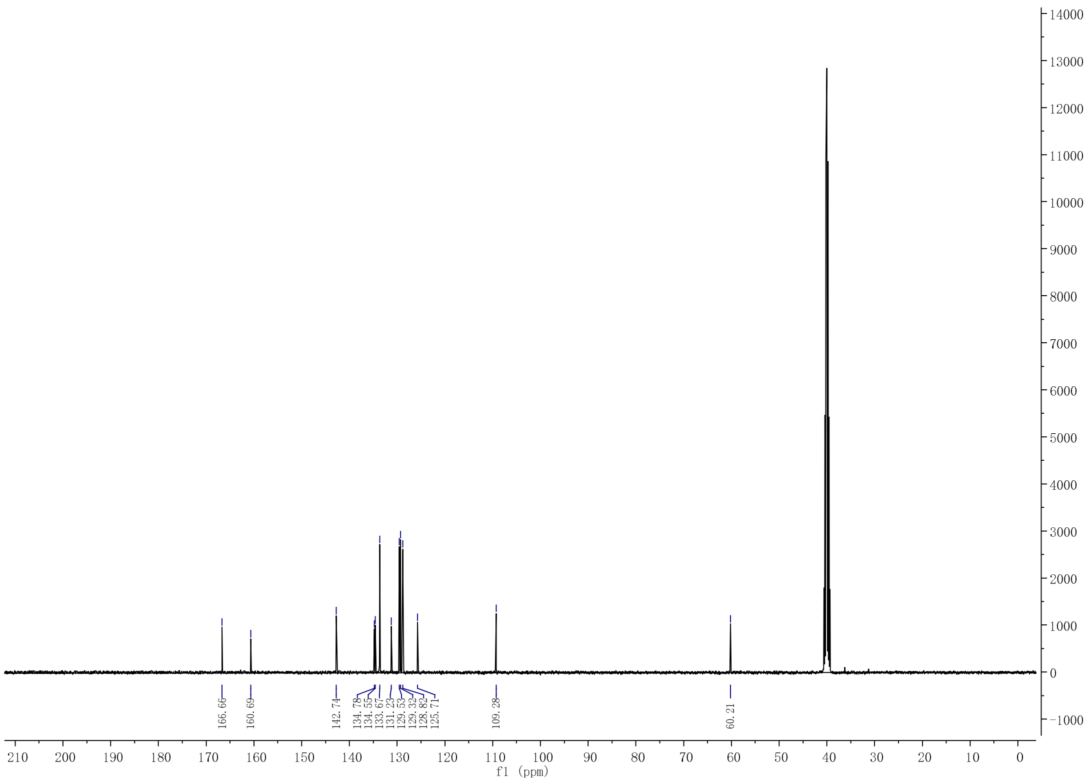


**$^{13}$C NMR of compound 5ab**

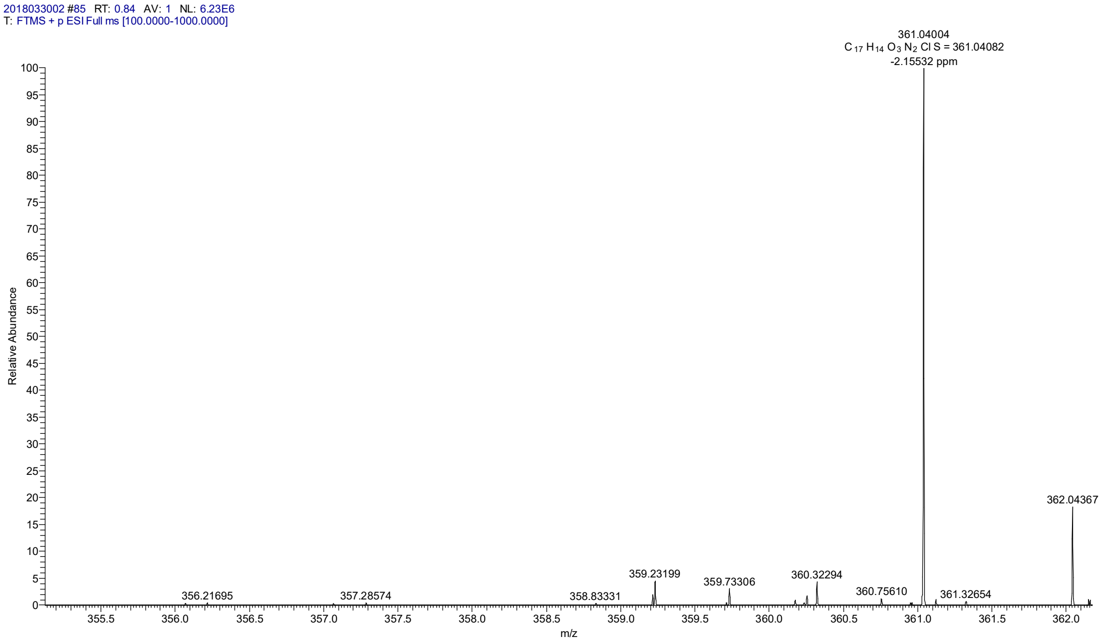


**HRMS (ESI) for compound 5ab**

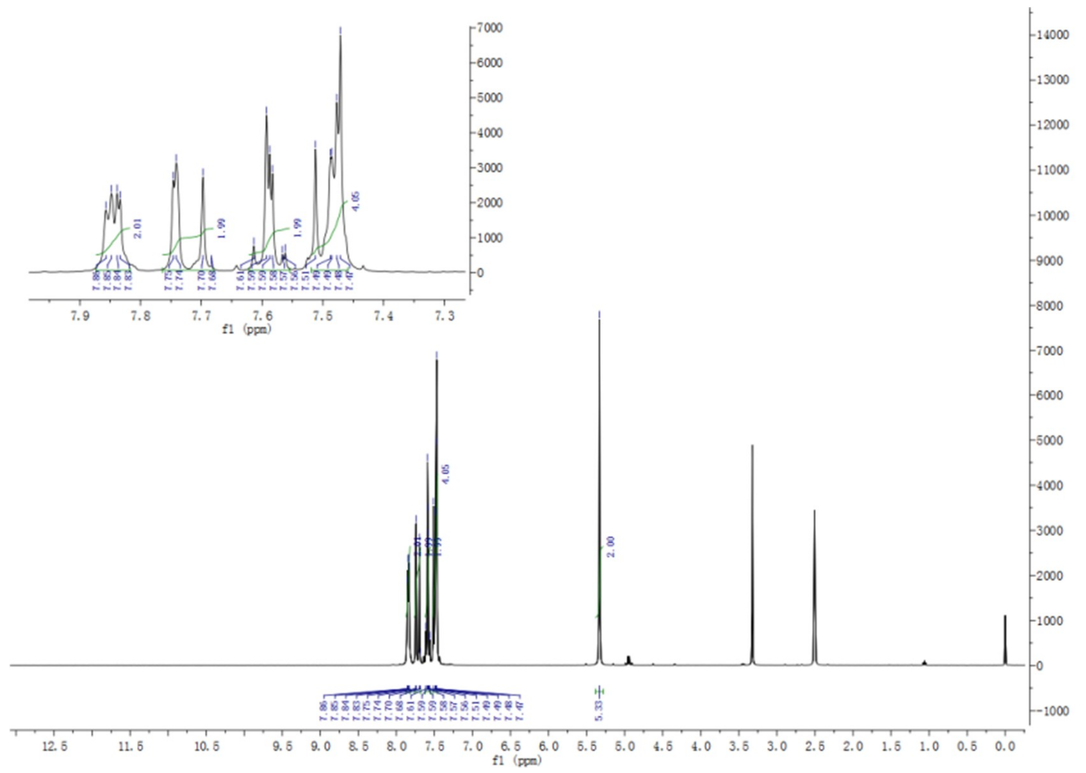

**¹H NMR for compound 5ac**

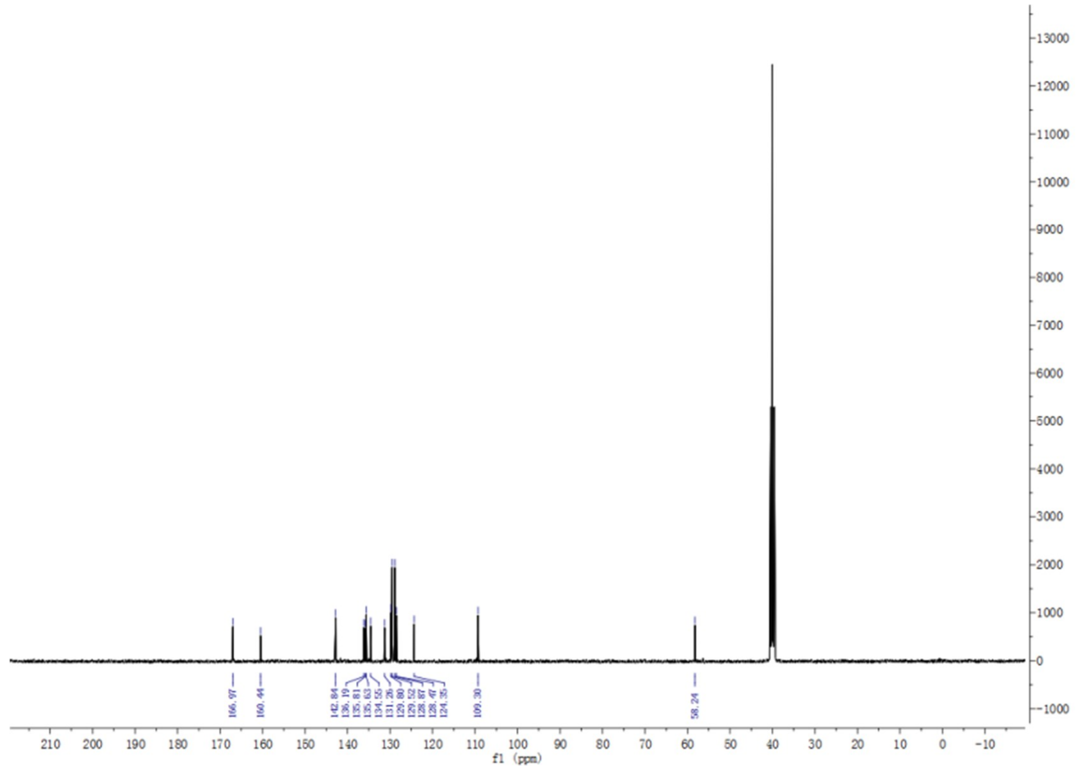

**¹³C NMR of compound 5ac**

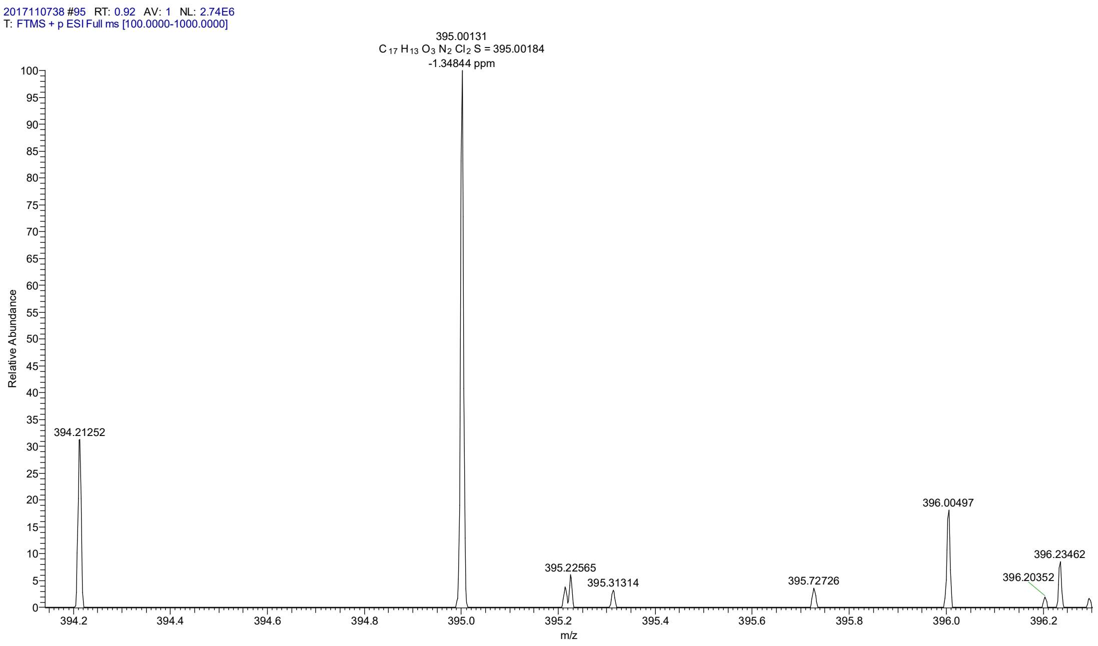


**HRMS (ESI) for compound 5ac**

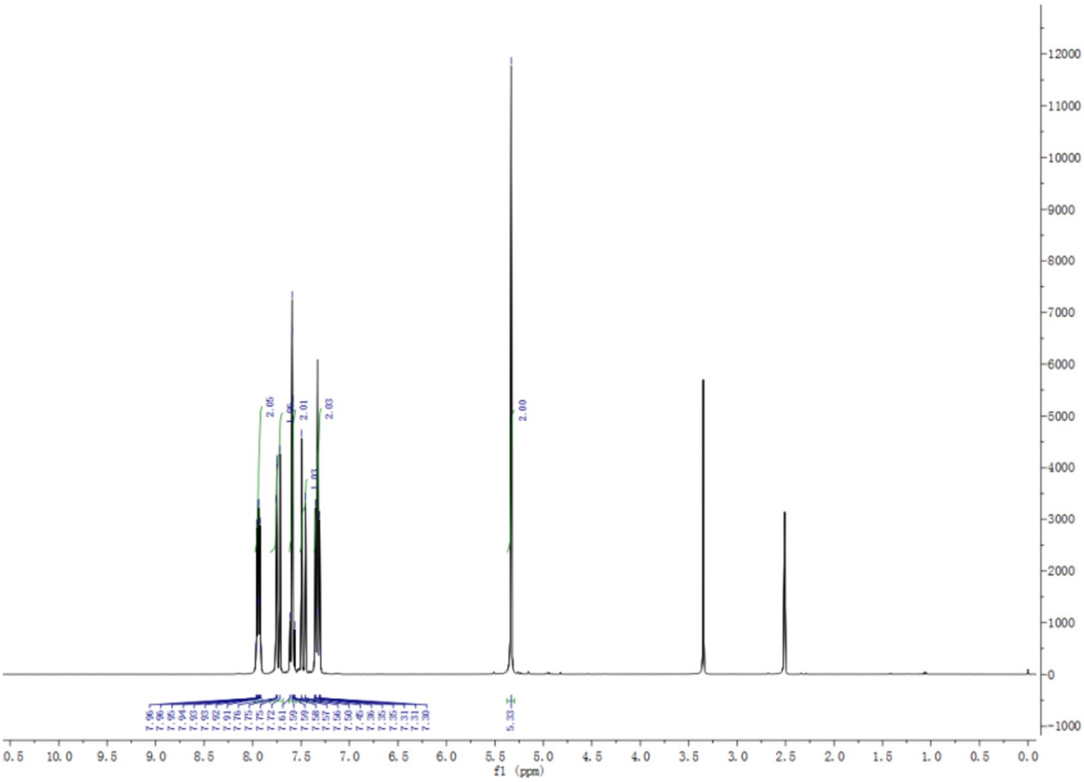


**$^1$H NMR for compound 5ad**

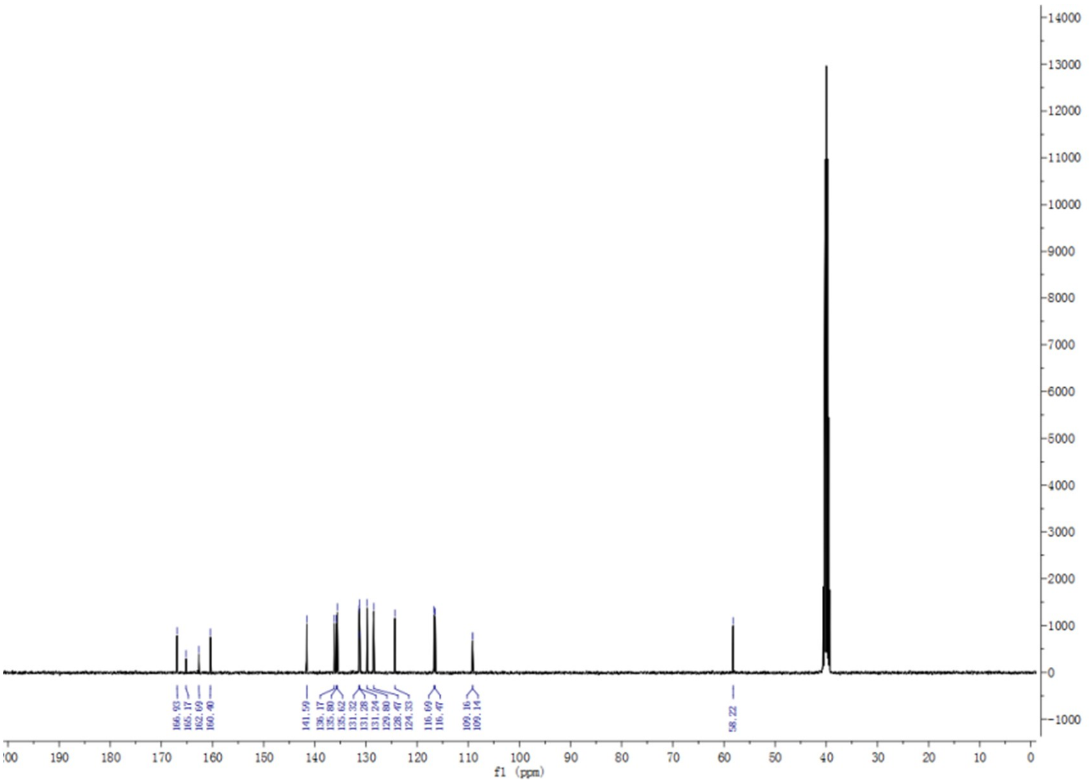

$^{13}C$ NMR of compound 5ad

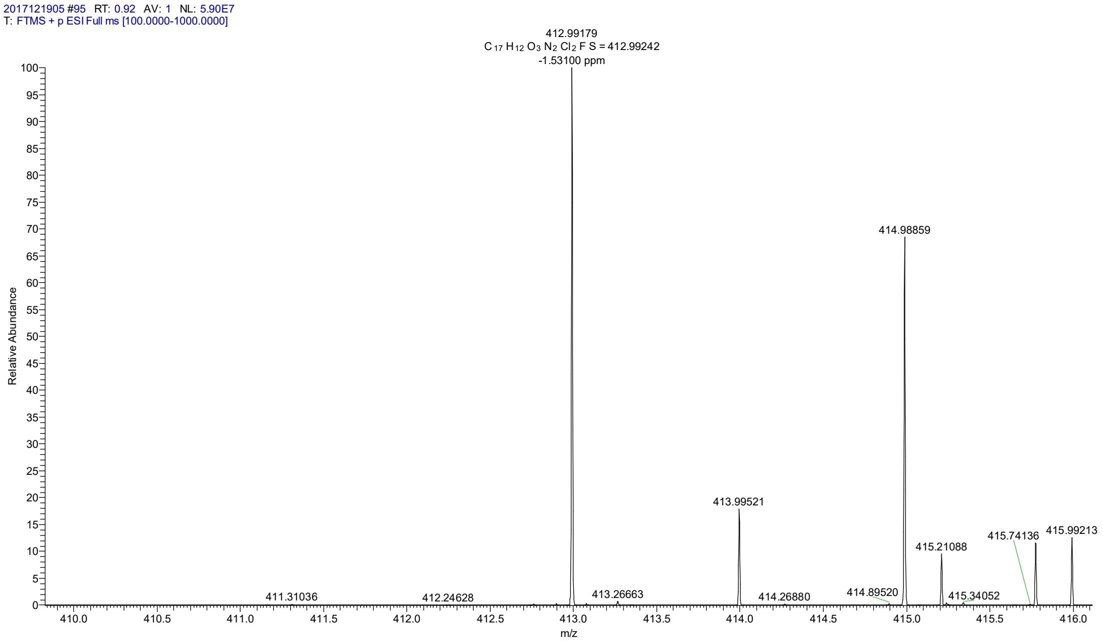


HRMS (ESI) for compound 5ad

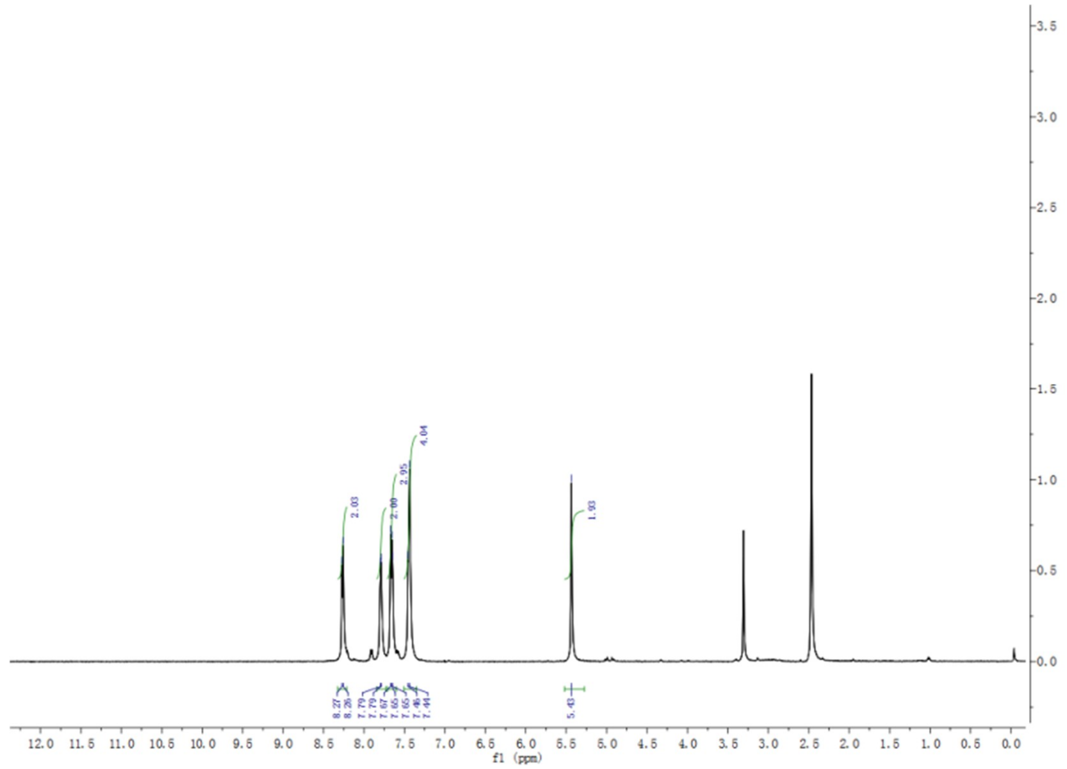


$^{1}$H NMR for compound 5ae

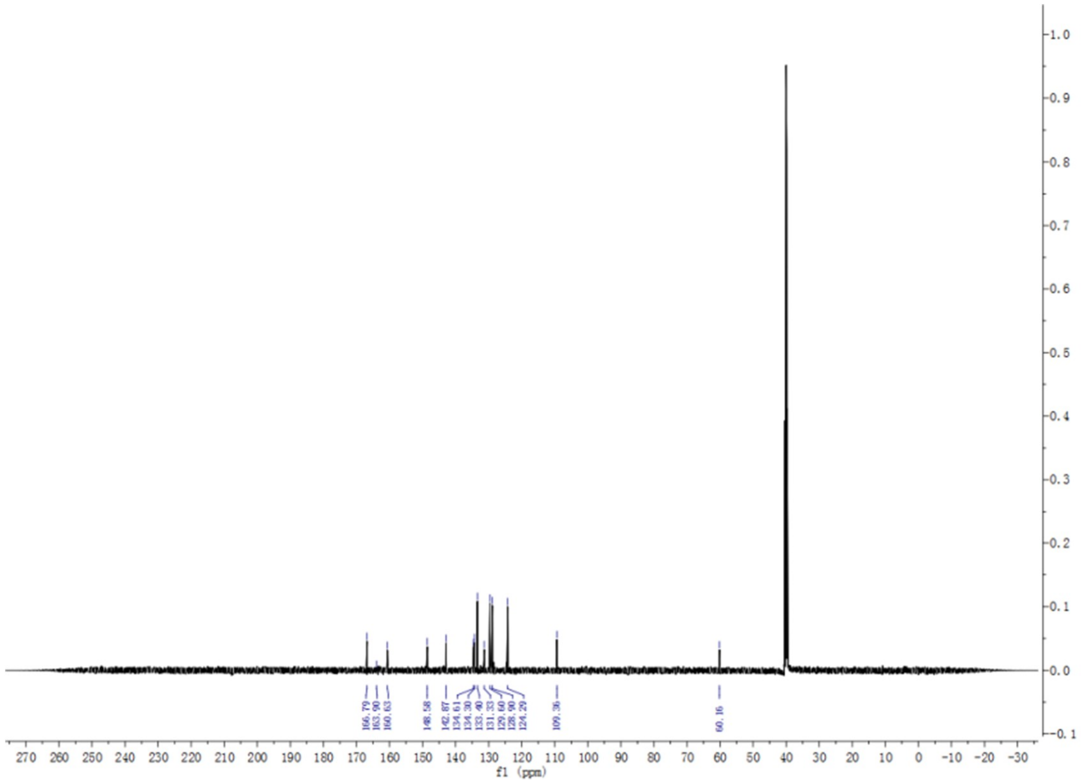


$^{13}$C NMR of compound 5ae

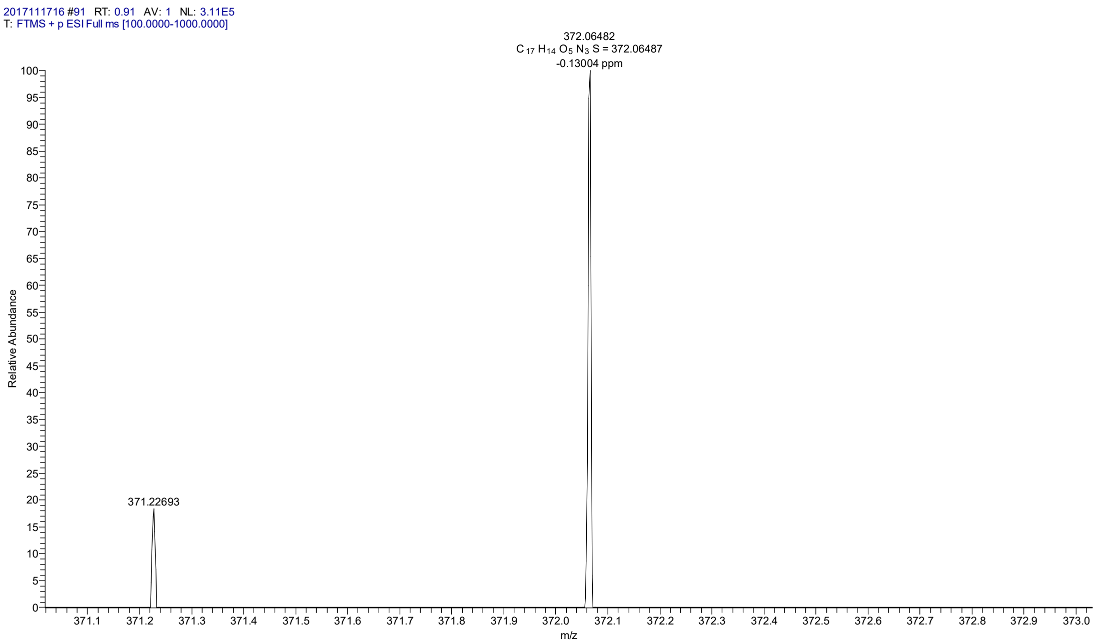


**HRMS (ESI) for compound 5ae**
